# Integrative multi-omics and machine learning reveals the spatial niche distribution and role of CYP27A1^+^TAMs in immunotherapy response in non-small cell lung cancer

**DOI:** 10.3389/fimmu.2026.1782545

**Published:** 2026-02-26

**Authors:** Qingsheng Liu, Xufeng Liu, Han Zhang, Yuhang Jiang, Ying Shi, Qiuqiao Mu, Yuhao Jing, Daqiang Sun

**Affiliations:** 1 Clinical School of Thoracic, Tianjin Medical University, Tianjin, China; 2 Tianjin Binhai New Area Haibin People’s Hospital, Tianjin, China; 3 Tianjin Chest Hospital, Tianjin University, Tianjin, China

**Keywords:** CD8+T cells, CYP27A1+TAMs, non-small cell lung cancer, spatial niche, tumor-associated macrophages

## Abstract

**Background:**

The response rate to immune checkpoint blockade (ICB) in non-small cell lung cancer (NSCLC) varies significantly among individuals. Cancer-associated macrophages (TAMs) are key components of the tumor immune microenvironment (TIME), influencing tumor proliferation, metastasis, immune cell recruitment, and activation through diverse mechanisms. Their high heterogeneity, particularly in the context of immunotherapy, warrants further investigation.

**Methods:**

We integrated single-cell and spatial transcriptomic data from the same patients using ISCHIA to construct nine spatial niches(local cellular communities). The composition of these niches was compared across different spatial regions and between samples with varying ICB treatment responses. CYP27A1^+^TAMs, identified as critical in ICB-responsive groups, were validated through external cohorts, immunohistochemistry, immunofluorescence, and *in vivo* experiments.

**Results:**

Spatial niche analysis revealed that niche 9, which was enriched with effector cells, was found exclusively in ICB responders. CYP27A1^+^TAMs were a key component of this niche, recruiting CD8^+^T cells via antigen presentation and chemokine secretion, thereby improving patient prognosis. Based on this, we developed an accurate prognostic model. Following ICB treatment, these macrophages exhibited further activation of LXR and enhanced anti-apoptotic capabilities. *In vivo* and morphological experiments demonstrated that CYP27A1^+^TAMs effectively suppressed tumor growth and increased CD8^+^T cells infiltration in the TIME.

**Conclusion:**

This study highlights the importance of spatial niches in understanding the TIME of NSCLC and predicting ICB responses. CYP27A1^+^TAMs and their downstream LXR pathway provide a novel research direction for exploring potential biomarkers for personalized NSCLC management.

## Introduction

Lung cancer is the most common cancer worldwide, with the highest incidence and mortality rates. Non-small cell lung cancer (NSCLC) accounts for 85% of all lung cancer cases (1). The treatment of NSCLC is becoming increasingly diversified, with neoadjuvant targeted therapy and neoadjuvant immunotherapy bringing new hope to patients (2). However, the overall response rate to Immune checkpoint blockade (ICB) treatments is relatively low, and resistance often occurs (3). While immunotherapy can remodel the Tumor Immune Microenvironment (TIME) towards a more anti-tumor state, the remodeled TIME often remains more immunosuppressive than that of normal tissues (4). The specific mechanisms of resistance to ICB remain largely unknown.

Single-cell sequencing has played a revolutionary role in exploring the tumor microenvironment, particularly in understanding cell functions (5). However, single-cell technology inevitably loses relative spatial information during the preparation of cell suspensions. Spatial transcriptomics technology effectively overcomes this limitation. By mapping spatial niches—functional units defined by specific cell types/states, their locations, molecular profiles, and interactions with neighboring cells—this approach helps us achieve a comprehensive and three-dimensional understanding of the TIME (6).

Tumor-associated macrophages (TAMs) are the most important myeloid cells in the tumor microenvironment. Unlike fibroblasts, which exhibit significant stromal tropism, TAMs are widely distributed around the tumor and within the stroma. Previous studies have shown that they generally play pro-inflammatory (M1) and anti-inflammatory (M2) roles. The former involves functions such as cytokine secretion, immune cell killing, and antigen presentation, while the latter is characterized by suppression of immune cells and promotion of immune escape (7). SPP1^+^TAMs are a well-characterized subset often associated with an M2-like, pro-tumorigenic phenotype that not only fosters an anti-inflammatory microenvironment but also interacts with tumor-associated fibroblasts (CAFs) to form a fibrous immune barrier at the tumor edge, preventing T-cell infiltration into the tumor core and playing a critical role in ICB resistance (8, 9). However, TAMs are distributed across the entire tumor microenvironment and even in normal tissues, exhibiting significant heterogeneity, making it difficult to distinguish them from a single perspective (10). Recently, new TAM subtypes such as TREM2^+^TAMs, lipid-laden macrophages and cancer-associated macrophage-like cells (CAMLs) have been identified (11–13), suggesting that macrophages should be carefully evaluated from a functional perspective. Additionally, research on TAMs that are also active in the stroma remains relatively superficial.

In our study, we integrated single-cell and spatial transcriptomics samples and used spatial niche methods to construct a global view of TIME. We analyzed the cellular composition and pro-fibrotic characteristics of the infiltrating tumor microenvironment from a new perspective. More importantly, we identified a unique TAM subpopulation, characterized by high expression of CYP27A1 (a key enzyme in cholesterol metabolism), hereafter referred to as CYP27A1^+^TAMs), which is widely distributed in the peritumoral and stromal regions of patients responding to immunotherapy. It primarily recruits T cells to exert anti-tumor effects and improve patient prognosis. Furthermore, immunotherapy enhances the expression of LXR ligands and receptors and strengthens their co-localization with T cells. Our study provides new insights into the spatial ecology of NSCLC and identifies CYP27A1 and LXR as potential biomarkers for predicting immunotherapy response, which may inform future clinical decision-making.

## Methods

### Single-cell RNA sequencing data processing, integration, and clustering

Single-cell RNA sequencing (scRNA-seq) data processing was performed using Seurat (v.5.2.1) (14) in R (v.4.4.0). The dataset comprised 19 samples (12 post-operative, 7 pre-operative biopsies). Low-quality cells were filtered based on the following criteria: nFeature_RNA > 300, nCount_RNA > 800, and percentage of mitochondrial genes (percent.mt) < 15. Gene expression counts were normalized using a scale factor of 10,000. Highly variable features were identified using the FindVariableFeatures function, selecting the top 5000 features. Data scaling was performed using default parameters. In the batch effect correction process, considering that the sequencing data were derived from two different platforms, we used the sequencing platform (BD/XGY) as the primary covariate for Harmony correction to eliminate systematic differences introduced by the varying technical platforms. We also recognized that other factors such as the source of patient samples and sample preparation dates might introduce variations. In this study, sample information was incorporated as additional correction covariates into the Harmony algorithm. This ensured that the corrected data not only removed technical batch effects but also minimized the potential confounding effects from differences in sample collection sites. Dimensionality reduction was carried out using Principal Component Analysis (PCA), with the first 30 principal components used for downstream analysis. Clustering was performed based on these Harmony-corrected embeddings at a resolution of 0.4. Major cell populations were annotated based on the expression of canonical marker genes: Epithelial cells (EPCAM, KRT19), Endothelial cells (VWF, CDH5), Mast cells (GATA2, KIT), Fibroblasts (PDGFRA, FAP), Pericytes (ACTA2, RGS5), Myeloid cells (CD14, FCGR3A), T cells (CD3D, CD3E), B cells (MS4A1, CD79B), and Plasma cells (MZB1, JCHAIN).

### Spatial transcriptomics data processing and deconvolution

VISIUM Spatial transcriptomics (ST) data were also processed using Seurat (v.5.2.1). Spots with total counts less than 200 and genes detected in fewer than 10 spots were filtered out. Data were read using Seurat.v5, specifying high-resolution images and their corresponding scale factors. Data layers were merged using JoinLayers, and normalization was performed using the SCTransform method. Spatial feature plots were generated to visualize gene expression and cell type distributions. For cell type annotation of spatial spots, deconvolution was performed using spacexr [Robust Cell Type Decomposition (RCTD)] (v.2.2.1) package (15). A reference was built using the raw count matrix from the annotated scRNA-seq data, focusing on the 17 most abundant and representative cell types. The total UMI count per cell (nUMI) was calculated for the reference. For each spatial sample, the raw count matrix was extracted, and nUMI per spot was calculated. Spatial coordinates for each spot were obtained using GetTissueCoordinates. A SpatialRNA object was created for each spatial sample by combining the count matrix, nUMI vector, and coordinate data frame. RCTD was run in “full” mode with default parameters. The resulting cell type weight matrices for each spot were added as an assay to the Seurat object. Cell type localization was visualized using SpatialFeaturePlot. Additionally, the vizAllTopics function from the STdeconvolve(1.3.2) package (16) was used to visualize RCTD results. Spatial co-localization analysis was performed using SpatialFeaturePlot with default parameters.

### Cell subpopulation identification

For subcluster analysis of major lineages, cells from each population were subset, re-normalized, and scaled. Harmony was re-run on each subset to mitigate residual batch effects, using a clustering resolution of 0.4. Myeloid cells were subclassified into Monocytes (FCN1, VCAN), Macrophages (CD68, CD163), Neutrophils (FCGR3B, CXCR2), and Dendritic cells (CD1C, CLEC10A) based on their respective canonical markers. Macrophages were further subdivided into five subsets: CCL4L2^+^TAMs, CYP27A1^+^TAMs, FGL2^+^TAMs, LPL^+^TAMs, and SPP1^+^TAMs. T cells were subclassified into CD8^+^T cells (CD8A, CD8B), CD4^+^ T cells (IL7R, CCR7), T regulatory cells (Treg; FOXP3, IL2RA), Cycling T cells (MKI67, TOP2A), and NK cells (FCGR3A, GNLY). Fibroblasts were subclassified into inflammatory Cancer-Associated Fibroblasts (iCAFs; IL6, CXCL12) and myofibroblastic Cancer-Associated Fibroblasts (myCAFs; ACTA2, POSTN). Clusters exhibiting mixed expression of markers from distinct lineages, potentially representing doublets despite prior doublet removal, were identified and excluded from downstream analysis due to the inability to precisely estimate the doublet rate post-filtering.

### CNV inference for malignant cell identification

To reliably distinguish malignant epithelial cells from normal epithelial cells, copy number variation (CNV) was inferred using the inferCNV package (v.1.22.0). To account for platform-specific batch effects, inferCNV was run individually for each sample. Endothelial cells were used as reference cells. Analysis was performed with default parameters. The output files infercnv.observations and infercnv.references were read, and CNV scores were calculated per cell. The top 5% of cells with the highest CNV aberration scores were selected. A similarity matrix was computed for all cells against this malignant reference set. A threshold of 0.20 was applied, and cells within the top 20% of similarity scores were classified as malignant for each sample.

### Spatial niche analysis

Spatial niches, termed Composition Clusters (CCs), were identified using Identifying Spatial Co­occurrence in Healthy and InflAmed tissues (ISCHIA) (V1.0.0.0) (17), which detects spatial co-occurrence patterns of cell types. The cell type weight matrix generated by RCTD and the integrated multi-sample Seurat object were used as input. The optimal number of niches was determined by evaluating the Calinski-Harabasz Index, Gap Statistic, and Elbow Method, collectively indicating 9 as the optimal number of clusters for subsequent analysis. For analyzing co-occurrence within specific niches (e.g., CC4 and CC6), a probability threshold (prob.th) of 0.05 was used. The distribution of niches across sample groups was visualized using the dittoSeq package (v.1.18.0).

### Pathway activity enrichment in niches using PROGENy

Pathway activity scores for different spatial niches were inferred using the PROGENy(v.1.28.0) (18) method on the integrated spatial transcriptomics data. Activity scores for hallmark pathways were calculated using the SCT assay, without additional scaling (scale = FALSE), using the top 1000 variable genes for model building. The resulting PROGENy score matrix was centered and scaled using the ScaleData function and visualized via heatmap.

### Cell type proportion analysis within niches

Cell type proportions within each niche were analyzed using two methods. Direct Proportion Calculation: RCTD-derived cell type annotations were aggregated by niche, and the proportion of each cell type relative to the total number of cells in each niche was calculated. Results were visualized using boxplots.Gene Set Enrichment-based Scoring. To mitigate issues with very low cell type proportions (e.g., B cells in CC4/CC6), cell type signature scores were calculated. The top 25 significant marker genes (by logFC) for each cell type, identified using FindAllMarkers, were compiled into gene sets. Enrichment scores for these gene sets per spot were calculated using the escape.matrix function from the ESCAPE package (v.2.2.4) (19). The distribution of these enrichment scores across niches was then visualized using boxplots.

### Single-cell pseudotemporal analysis of macrophages

To investigate macrophage state transitions and pseudotemporal ordering, macrophages from pathological complete response (pCR) patients were subset from the Seurat object. Pseudotime analysis was performed using Monocle (v.2.34.0). A CellDataSet object was constructed following the package vignette. Highly variable genes were identified with min_expr = 0.3. Genes were retained for dimensionality reduction if their mean expression was >= 0.5 and their empirical dispersion was at least 1.5 times that expected under a null model. Dimensionality reduction was performed using DDRTree, and cell ordering was established using orderCells with default parameters. For branch analysis, branch1 was selected as the branch point. Gene Ontology Biological Process (GO-BP) enrichment analysis was performed on genes associated with identified states/branches.

### Spatial trajectory construction and enrichment analysis with SPATA2

Spatial trajectory analysis was performed using SPATA2 (v.3.1.4) (20). The Seurat object was converted to a SPATA2 object using asSPATA2. A spatial trajectory was constructed spanning CC2, CC6, and CC4 using createSpatialTrajectories, with a width of 9.78 units and a length of 137.96 units. Gene expression data and trajectory coordinates for spots along the trajectory were extracted. Hallmark gene sets (obtained from msigdbr v.7.5.1) were used for Gene Set Variation Analysis (GSVA) along the trajectory. Trajectory coordinates were transformed into a one-dimensional pseudotime variable ordered along the trajectory direction. A trajectory heatmap was generated, adapting the plot_trajectory_heatmap function from the referenced methodology [Multimodal decoding of human liver regeneration (21)] and customizing aesthetics due to SPATA2 version differences. Spatially variable genes were identified using runSPARKX and filtered (threshold_pval = 0.05, fdr < 0.5) for visualization.

### GSVA for macrophages functional states in scRNA-seq

To characterize functional states of macrophages subpopulations, GSVA(v.1.52.3) (22) was performed. Gene sets related to macrophages function were selected from the Kyoto Encyclopedia of Genes and Genomes (KEGG) pathway collection. The average expression matrix for each macrophage subtype was calculated using AverageExpression, and the RNA assay matrix was extracted. Pathway enrichment scores were computed using the gsvaParam method and the gsva function with default parameters. To compare functional states before and after immunotherapy, the add.ident parameter was used to group cells by timepoint before calculating the average expression matrix, using the same gene sets and GSVA parameters.

### Cell-cell communication analysis

Cell-cell communication analysis was performed using CellChat(v.2.1.2) (23), which incorporates spatial distance constraints. Analyses were run separately for different niches and overall cell types. RCTD-derived cell type weights and the SCT normalized expression matrix were used. Spatial coordinates for each sample were provided separately. A spatial factors object was created for each sample by calculating the micron-per-pixel ratio (based on each spot has a diameter of 55 micrometers, with a spacing of 10 micrometers between adjacent spots). The interaction.range and contact.range parameters were set to 250 and 100, respectively. The databases “Secreted Signaling”, “ECM-Receptor”, and “Cell-Cell Contact” were used. Communication probabilities were computed, filtering out interactions with fewer than 10 potential links (filterCommunication). Pathway-level communication (computeCommunProbPathway) and aggregated networks (aggregateNet) were calculated. Co-expression of ligand-receptor pairs (e.g., CXCL9-CXCR3) was visualized using SpatialFeaturePlot with a minimum cutoff min.cutoff = 0.05.

### Spatial co-localization analysis of cell types based on Ripley’s K-function

A quantitative assessment of the spatial association between CYP27A1^+^tumor-associated macrophages (TAMs) and CD8^+^T cells was conducted. First, based on the deconvolution analysis results, positive spots were defined using a thresholding method (spots with CYP27A1^+^TAMs/CD8^+^T cell expression exceeding the 25th percentile of the corresponding expression in all spots were considered positive spots). The spatial coordinates of positive spots were extracted, and samples with fewer than 40 spots for any cell type were filtered out. Multi-type point pattern data were constructed using the spatstat package (v.3.3-1). Cross-K-function (Kcross) and L-function (Lcross) analyses were employed to examine the association patterns between the two cell types at different spatial distances. To assess statistical significance, Monte Carlo simulations were used to calculate 95% confidence intervals, with the null hypothesis being spatial independence between the two cell types. Effect sizes were quantified by calculating the standardized mean deviation (Cohen’s d) of L(r)-r from 0 and the aggregation index (Aggregation Index, proportion of distances where L(r)-r > 0). Spatial distances were converted to actual micrometer units for analysis based on the original image scale.

### Spatial cell-type co-localization analysis with MISTY

Spatial co-localization between cell types was analyzed using MISTY (v.1.14.0) (24). Based on the image scale factor, the view distances were set as follows: juxta view = 200 µm, para view = 3000 µm. The zoi parameter for the para view was set to 200 µm to mask the areas already covered by the intra and juxta views. Prior to analysis, an offset of 10,000 units was added to the X and Y coordinates of each sample to prevent coordinate overlap. Analyses were run with default parameters otherwise. Results were summarized across samples by calculating the median standardized importance for each interaction. Results were visualized via heatmap and community interaction plots (plot_interaction_communities), using a cutoff of 1.5 for importance values in all views for community detection.

### Survival analysis in TCGA cohort

RNA-seq data (IlluminaHiSeq, n=576 samples) and corresponding clinical/survival data for the TCGA-LUAD cohort were downloaded from UCSC Xena (http://xena.ucsc.edu/).For CYP27A1 expression analysis, samples were dichotomized into high and low expression groups using an optimal cutoff determined by the survminer package (v.0.5.0). Overall survival (OS) was compared between groups using Kaplan-Meier curves generated with the survival package (v.3.8-3), and the log-rank test was used to calculate p-values. For CYP27A1^+^TAMs signature analysis, the marker genes (by logFC) for the CYP27A1^+^TAMs subset were defined as a gene signature. A single-sample Gene Set Enrichment Analysis (ssGSEA) score was calculated for each sample in the TCGA cohort based on this signature. Samples were split into high and low signature score groups based on the median score. Kaplan-Meier survival analysis was performed as described above.

### Correlation analysis in TCGA and scRNA-seq cohorts

Signature scores for TAMs-LXRhi, TAMs-LXRlow, and CD8^+^T cells (defined by the top marker genes from scRNA-seq, ranked by logFC) were calculated in the TCGA-LUAD cohort using ssGSEA. Pairwise Pearson correlations and associated p-values between these signature scores were computed using cor.test. scRNA-seq Cohort: The proportions of TAMs-LXRhi and TAMs-LXRlow cells among all macrophages, and the proportion of CD8^+^T cells among all T cells, were calculated for the scRNA-seq cohort. Pairwise Pearson correlations between these proportions were computed using cor.test.

### Validation cohort processing and analysis

External validation datasets were processed with standardized pipelines: scRNA-seq Cohort GSE131907: Cells were filtered (nFeature_RNA > 300, nCount_RNA > 1000, percent.mt < 15), resulting in 201,003 cells. scRNA-seq Cohort GSE223203: Cells were filtered (nFeature_RNA > 750, nCount_RNA > 1500, percent.mt < 15), resulting in 33,443 cells. Spatial Transcriptomics Cohorts (E-MTAB-13530, GSE267960): Spots were filtered (genes with < 10 counts removed, spots with < 200 total counts removed). E-MTAB-13530 yielded 57,238 spots; GSE267960 yielded 9,403 spots. Cell type annotation for the spatial validation cohorts was performed using RCTD (“full” mode), with the annotated GSE131907 scRNA-seq data serving as the reference. MISTY analysis was performed using the same parameters as for the primary spatial cohorts.

### Construction of the CMRS prognostic model

During the development of the prognostic model, differential gene expression analysis was first performed. Differentially expressed genes (DEGs) between tumor and normal tissues in the TCGA-LUAD dataset were identified using the criteria of LogFC > 1.5 and Padj < 0.05. Concurrently, marker genes for CYP27A1^+^TAMs were selected based on thresholds of LogFC > 0.25 and Min.pct = 0.25. The intersection of these two gene sets yielded 108 candidate genes.

Univariate Cox regression analysis was then applied to these 108 genes within the TCGA-LUAD cohort to identify those with a significant impact on prognosis. We selected the 24 genes demonstrating the strongest prognostic associations. However, due to the absence of CD302 and C4orf48 expression data in several validation cohorts, a final set of 22 genes was utilized for model construction.

For the model building phase, we systematically evaluated 101 distinct combinations of machine learning algorithms, including Random Forest (25), CoxBoost (26), Elastic Net (27), Gradient Boosting Machine (GBM) (28), Lasso (29), plsRcox (30), Ridge (31), StepCox, SuperPC (32), and Survival-SVM (33). The optimal algorithm combination was selected based on the highest average C-index across cohorts, forming the final CMRS (CYP27A1^+^Macrophage Risk Score). To assess the robustness of the CMRS model and address potential overfitting concerns, we performed comprehensive stability and calibration analyses. Bootstrap resampling with 1000 iterations was conducted on the TCGA-LUAD training cohort to evaluate the consistency of C-index values. Additionally, we examined C-index distributions across 500 different random seeds (80:20 training-validation splits) to assess model performance variability. Calibration curves were generated for 1-, 3-, and 5-year survival predictions using the bootstrap method across all validation cohorts. The Hosmer-Lemeshow test was applied to statistically evaluate the agreement between predicted and observed survival probabilities at each time point. The robustness and superior performance of CMRS were subsequently validated through multiple approaches: comparative analysis of C-indices against previously published models, assessment of AUC values across different validation cohorts, and demonstration of significant prognostic differences between high-risk and low-risk patient groups stratified by the median CMRS. The coefficients (COEF) of the genes in the CMRS have been provided in **Supplementary Material 1**.

### RAW 264.7 cell line processing

We achieved overexpression of CYP27A1 in RAW by lentiviral transduction. The treatment group was infected with CYP27A1-overexpressing lentivirus at an MOI of 10 (RAW-OE), the negative control group was treated with NC lentivirus at an MOI of 10(RAW-NC), and the blank control group received no additional treatment (RAW). All cells were maintained in a humidified incubator at 37 °C with 5% CO_2_.

### Animal model

All animal procedures in this study were approved by the Animal Ethics Committee of Tianjin Chest Hospital. The C57BL/6 female mice were obtained from Si Bei Fu Biotechnology (Beijing, China) at 6–8 weeks of age were subcutaneously injected in the right inguinal region with Lewis lung carcinoma (LLC) cells resuspended in ice-cold PBS, at a density of 1×10^6^ cells per mouse. Tumor growth was monitored weekly by measuring the longest diameter with a vernier caliper. Starting from the second week after LLC injection, 1×10^6^ RAW were separately administered to mice in the experimental group, vector control group, and blank control group. On day 28 after LLC cell injection, mice were euthanized by inhalation of excess CO2, with the CO2 flow rate maintained at 30–50% of the chamber volume per minute. Death was confirmed by cessation of respiration, dilated pupils, and no response to toe pinch. Subsequently, tumors were immediately dissected and removed in full.

### RNA extraction and RT-PCR analysis

Total RNA was extracted from cells using TRIzol reagent (Thermo, #15596018) following the manufacturer’s instructions. cDNA was synthesized using the PrimeScript RT Master Mix kit (Vazyme, #R232-01). Quantitative real-time PCR was performed with SYBR Green Master Mix (Vazyme, #Q111-02). GAPDH mRNA was used as an internal control for normalization. Relative expression levels were calculated using the 2−ΔΔCt method. The primer used in the paper have been provided in **Supplementary Material 2**.

### Immunohistochemistry

Tumor tissues were fixed, paraffin-embedded, and sectioned. Deparaffinization was performed using xylene, followed by rehydration through a graded ethanol series. Antigen retrieval was carried out by incubating the sections in citrate buffer at 65 °C for 2 hours. Endogenous peroxidase activity was quenched with 3% hydrogen peroxide. The sections were then blocked with 5% bovine serum albumin (BSA) and incubated with specific primary antibodies (1:500). The remaining procedures were performed using an immunohistochemistry kit (ZSBIO, PV-9000).

### Immunofluorescence

Tumor tissues were fixed, paraffin-embedded, and sectioned. Deparaffinization was performed using xylene, followed by rehydration through a graded ethanol series. The sections were then blocked with 5% BSA. Immunofluorescence staining was carried out using an Immunofluorescence System Kit (Yeasen,60410), with primary antibodies diluted at 1:200. Three antibodies were incubated using the TSA method. Cell nuclei were stained with DAPI. After staining, the sections were imaged using a 3DHISTECH fluorescence slide scanner and analyzed with SlideViewer software.

### Western blot

Western blotting was performed according to standard protocols. Proteins were extracted using RIPA lysis buffer, and concentrations were determined via the BCA assay. Primary antibodies used included those against CYP27A1 (1:2000) and GAPDH (1:5000). A goat anti-rabbit IgG HRP-conjugated antibody (1:5000) was used as the secondary antibody.

### Statistical analysis

Bioinformatics analyses were performed using R (v4.4.0). Basic experimental data were processed with GraphPad Prism and ImageJ. For comparisons between groups, Student’s t-test or one-way ANOVA was applied for normally distributed data, while the Wilcoxon rank-sum test or Kruskal–Wallis test was used for non-normally distributed data. Survival analysis was conducted using the Kaplan–Meier method, and differences were assessed with the log-rank test. Correlations were evaluated using Spearman’s rank correlation coefficient. A p-value < 0.05 was considered statistically significant, with levels of significance denoted as *P < 0.05, **P < 0.01, and ***P < 0.001.

## Result

### Single-cell and spatial transcriptomics sample preparation

**Figure 1:**
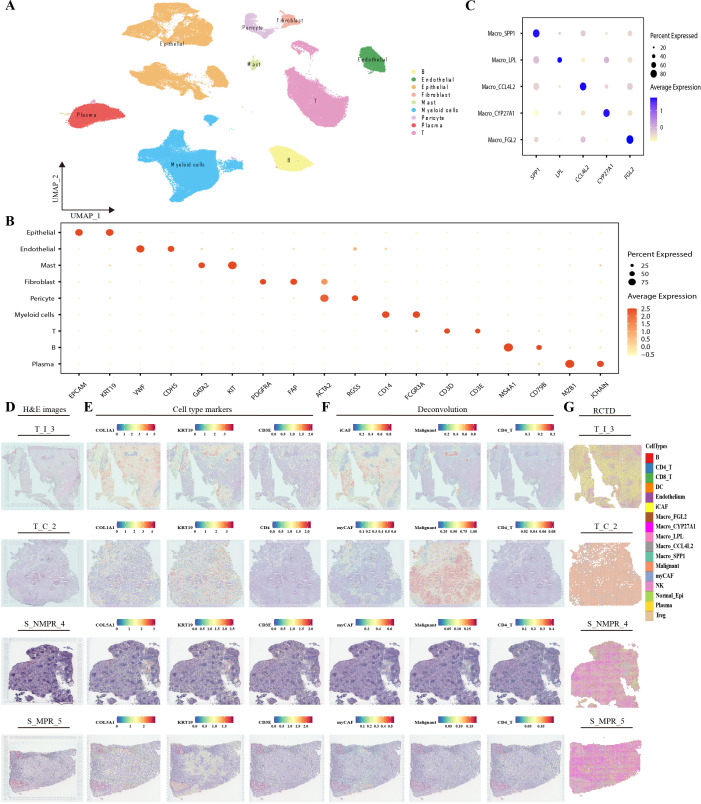
RCTD integrates results of SC and ST **(A)**. UMAP plot of 127943 cells from single-cell samples. **(B, C)** Dotplot showing the marker genes for clusters. The color gradient represents the scaled expression level, and the diameter represents the percentage of cells. **(B)** markers of major clusters, **(C)** markers of macrophages. **(D)** H&E micrographs of infiltrative tumor (T_I), tumor core (T_C), Stroma of non-major pathologic response (S_NMPR) and Stroma of major pathologic response (MPR). **(E)** Cell marker gene expression levels in T_I, T_C, S_NMPR and S_MPR samples. **(F, G)** RCTD deconvolution results of T_I, T_C, S_NMPR and S_MPR samples. RCTD, Robust Cell Type Decomposition; SC, single-cell; ST, spatial transcriptomics.

Single-cell and spatial transcriptomics data were obtained from Yan et al. and downloaded from Zenodo (9). The single-cell dataset comprised 19 samples, all from tumor tissues of NSCLC patients, including 7 from pre-operative biopsies and 12 from surgical resections. All patients had received chemotherapy combined with ICB therapy. Based on pathological response, they were categorized into 6 pathological responders (5 with pathological complete response, pCR, and 1 with major pathological response, MPR, collectively referred to as the pathological responders, PR group) and 13 non-major pathological responders (NMPR group). We performed re-quality control on the data, resulting in 148,812 high-quality cells. Batch effects were removed, followed by re-dimensionality reduction, clustering, and cell population identification (**Supplementary Figure 1A**). Based on classical markers, cells from the 19 samples were categorized into 10 clusters (**Figures 1A, B**). Epithelial cells were further classified into normal epithelial and malignant epithelial cells using inferCNV based on copy number variations (**Supplementary Figures 1B, C**). T cells and myeloid cells were subjected to separate re-dimensionality reduction, clustering, and subpopulation analysis (**Supplementary Figures 1D, E**). Macrophages were specifically named according to their corresponding markers as: CCL4L2^+^TAMs, CYP27A1^+^TAMs, FGL2^+^TAMs, LPL^+^TAMs, SPP1^+^TAMs (**Figure 1C**).

Fifteen corresponding VISIUM spatial transcriptomics samples were all obtained post-operatively, including 6 tumor samples and 9 stromal samples. Among the 6 tumor samples, 2 were from the tumor core (T_C), 3 were isolated tumor masses, and 1 was invasive tumor (all collectively referred to as T_I). The 9 stromal samples comprised 5 from MPR and 4 from NMPR patients. After filtering out low-quality spots and genes, 24,810 genes and 59,387 spots remained. To characterize the TIME by integrating the gene expression matrices and spatial distribution information from both datasets, we applied RCTD. Using the single-cell data corresponding to the spatial samples, we selected 16 representative clusters to annotate the spatial transcriptomics samples. The annotation results were satisfactory: tumor samples were predominantly populated by tumor cells, with clear distinction between normal and malignant epithelium, while stromal samples were highly enriched with fibroblasts (**Figures 1D–G**). Notably, the deconvolution results were equally robust in identifying tertiary lymphoid structures (TLS), accurately pinpointing their immune cell composition, which was primarily comprised of B cells and T cells, consistent with literature reports (34–36).

By integrating and deconvoluting matched single-cell and spatial transcriptomics data, we reconstructed a detailed spatial cellular atlas. This comprehensive approach enables us to explore the tumor immune microenvironment holistically, from the complex genetic sequences to the intricate spatial architecture.

### Spatial niche analysis reveals the landscape of lung cancer following immunotherapy

**Figure 2:**
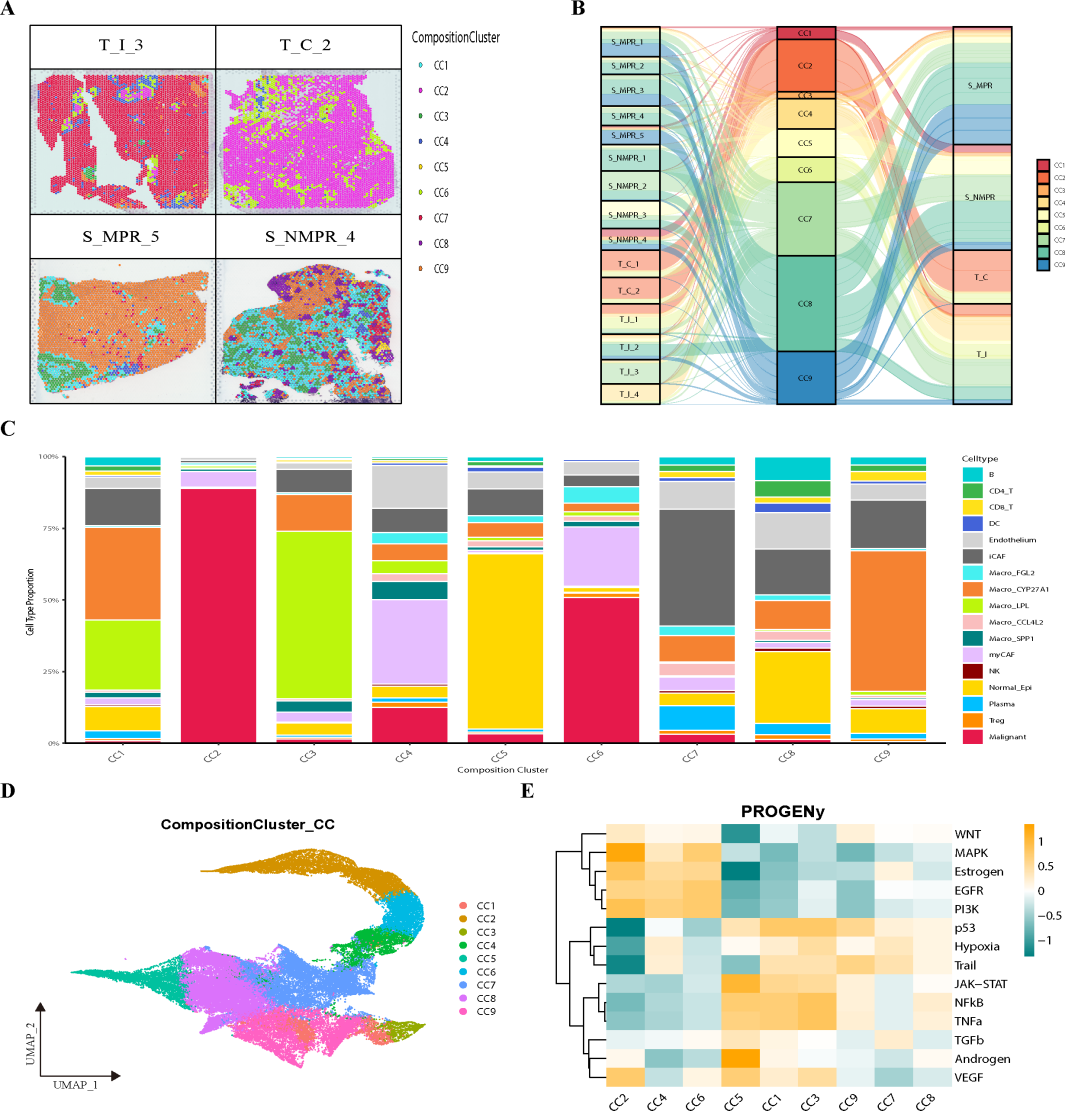
The construction and comparative analysis of spatial niches. **(A)** Visualization of spatial ecotypes in *TI, TC, SNMPR* and SMPR samples. CC, Composition Cluster. **(B)** Sankey diagram displaying the distribution of 9 CCs across different groups and samples. **(C)** Bar plots showing cell type composition of each spatial CC. **(D)** UMAP plot of ST spots clustered by cell type composition based k-means clustering colored by the nine spatial ecotypes. **(E)** Heatmap of PROGENy for scaled GSVA across ecotypes. CC, Composition Cluster; UMAP, Uniform Manifold Approximation and Projection; GSVA, Gene Set Variation Analysis.

To better understand the immune microenvironment of spatial samples at VISIUM resolution, we employed ISCHIA niche analysis to investigate the TIME from the perspective of cell communities. Using a co-occurrence analysis method to identify spatial neighborhoods, this clustering approach can recapitulate spatial aggregation and interaction tendencies, clustering a total of 59,387 spots into 9 distinct niches. Niches are local microenvironments within the tumor formed by specific cell populations (e.g., cancer cells, immune cells, stromal cells) and their interactions, termed Composition Cluster 1-9 (CC1-9) (**Figures 2A–C**; **Supplementary Figures 2A, B**). Based on the predominant cell types and spatial distribution tendencies, we categorized the 9 CCs into 4 classes. PROGENy pathway enrichment analysis and Uniform Manifold Approximation and Projection (UMAP) plots helped us quickly and intuitively understand the different niches (**Figures 2D, E**).

CC2, CC4, and CC6 belong to the first class, designated as tumor-enriched niches. Among these, CC2 has the highest proportion of malignant cells and is located at the furthest edge in the UMAP distribution. Tumor-enriched niches highly express pathways such as MAPK, EGFR, PI3K, and estrogen signaling, reflecting the active growth, proliferation, and invasive state of tumor cells (37–40). Furthermore, CC2 exhibits the lowest expression of tumor suppressor pathways like P53 and Trail, and the highest expression of VEGF (41–43), providing further evidence that it possesses the highest degree of tissue instability and metastatic potential.

CC7 and CC8 constitute the second class, termed tumor-stroma niches. They contain abundant iCAFs, plasma cells, and B cells (**Figure 2C**; **Supplementary Figures 2C, D**) and are primarily located in the stroma of T_I samples. Compared to tumor niches, they upregulate relatively more pro-apoptotic pathways, as well as inflammatory pathways like NFκB and TNFα (44–46). They foster complex inflammatory components within the stroma that regulate the tumor microenvironment.

CC5 is the third class, identified as the normal epithelium-enriched niche. It contains relatively few immune cells, resembling a normal lung epithelial environment. Its functional state also exhibits complexity: on one hand, it highly expresses the P53 pathway to maintain genomic stability; on the other hand, it highly expresses JAK-STAT signaling to regulate the immune system and cell proliferation. Additionally, it shows the highest expression of androgen signaling, which is opposite to the tumor-enriched niches. This reflects the ability of the CC algorithm to distinguish the tendencies of different cell types at the functional level.

CC1, CC3, and CC9 form the fourth class, termed immune-stromal niches. They are heavily clustered in stromal samples and enriched with various immune cells, particularly macrophages. It can be said that the functional differences of macrophages primarily constitute their main distinctions (**Figure 2D**). Notably, these niches showed minimal expression of invasiveness markers. Conversely, they also highly express P53. Their points of difference lie in the fact that CC9 has the highest expression of the Trail pathway, suggesting that immune cells within CC9 may tend to exert anti-tumor effects by promoting apoptosis. In contrast, CC1 and CC3 show highly activated inflammatory pathways, including NFκB and TNFα, indicating they might perform anti-tumor functions by activating more inflammatory responses. It is important to note that while CCs of the same type share functional similarities, different CCs possess their own unique heterogeneity, particularly in terms of cell functional states, which warrants further exploration.

Although both CC1 and CC9 are distributed within the tumor stroma, their distribution tendencies differ across samples with different pathological responses (**Figure 2B**). This quantifiable, clinically correlated distributional difference demonstrates that spatial niches can not only help us quickly understand the composition of the tumor microenvironment from a holistic perspective but can also serve as a comparable basic unit for deconstructing the heterogeneity of the tumor microenvironment. In summary, using 15 spatially transcriptomic samples annotated via deconvolution, we applied the ISCHIA algorithm to construct 9 spatial niches. By integrating transcriptomic functional changes, cellular composition, and spatial tendencies, we reconstructed the spatial architecture of the TIME from a new perspective and preliminarily compared their differences.

### Spatial trajectory and interaction analysis reveals the biological behavior of infiltrating tumors

To decipher the tumor ecosystem, we first focused on analyzing the tumor-enriched spatial niches. In the tumor ecosystem, CC2 is the most accumulated with tumor cells, predominantly composed of tumor cells and myCAFs, with very few immune cells, exhibiting a typical immune desert landscape. CC2 strictly exists in the tumor core and a small portion of infiltrating tumors. It is not present in stromal samples, consistent with pathological annotations, reflecting the reliability of annotations and ecological niche analysis. Similarly, CC6 is only enriched in the tumor core and infiltrating tumors, but compared to CC2, it is more distributed within infiltrating tumors and less in the tumor core. CC4 is more complex; its distribution in the tumor is similar to CC6, mainly present in infiltrating tumors. Interestingly, CC4 is the only one among the tumor-enriched ecosystems that is relatively distributed in the stroma, especially in MPR samples (**Figure 2B**). Since CC4 and CC6 are located at the tumor margins, often at the interface between tumor and normal tissue, understanding their roles is crucial for deepening our exploration of the tumor microenvironment.

**Figure 3:**
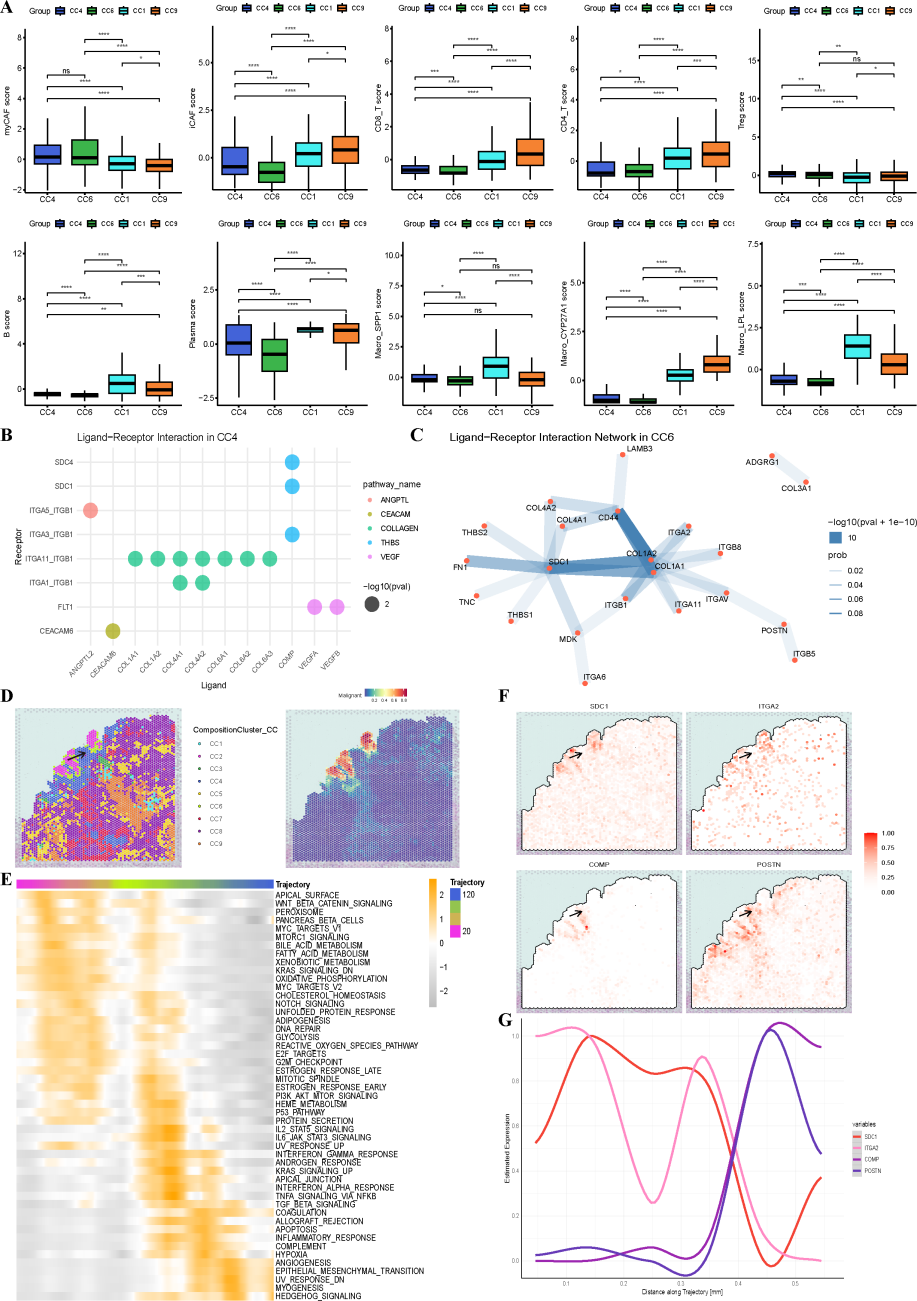
The tumor niche uncovers key cells and genes involved in tumor progression **(A)**. Box line plots comparing scaled ssGSEA scores for gene sets between CC1, CC2, CC3 and CC5 ecotypes. Gene set construction from single-cell samples. Use the Wilcoxon rank-sum test to compare component differences across the four groups. *P < 0.05; **P < 0.01; ***P < 0.001; ****P < 0.0001 **(B)** Dotplot shows cell communication in CC4. **(C)** Cnetplot shows cell communication in CC4. **(D)**Spatial feature plot shows spatial ecotypes and malignant in T_I_2, Arrow indicates spatial trajectories. **(E)** GSVA scores of the HALLMARK gene sets along the spatial trajectory in T_I_2. Different colors of the trajectories indicate the different ecological niches they occupy. **(F)** The expression levels of key genes involved in CC6 communication in ST samples, with arrows representing the spatial trajectories. **(G)** Trends in gene expression of the key genes involved in CC6 communication.

In terms of cellular composition, both are lacking T/B cells and are enriched with immunosuppressive cells such as SPP1^+^TAMs, Tregs, and myCAFs (**Figures 3A**, **Supplementary Figure 3A**). Notably, myCAFs tends to associate with tumor infiltration, whether in the tumor core or infiltrating tumor, consistent with existing studies (47). Comparing the spatial distributions, CC6 and CC2 are more directly related, often encapsulating the tumor core, whereas CC4 is relatively more marginal and often located at the periphery, surrounding CC4 and CC2. Analyzing ecosystem co-occurrence internally, we found that Tregs and other cell types, especially macrophages, are closely associated. Additionally, in CC4, co-occurrence of SPP1^+^TAMs and tumor cells was observed, These spatial patterns align with the known pro-tumor functions of these immunosuppressive cells. Specifically, SPP1^+^TAMs are reported to collaborate with myCAFs in remodeling the extracellular matrix and establishing an immunosuppressive microenvironment (48). Tregs, by suppressing effector T cell activity, create an immune-permissive environment for tumor growth and infiltration (49). Thus, the spatial co-occurrence of SPP1^+^TAMs, Tregs, and myCAFs likely creates a synergistic immunosuppressive circuit that enhances tumor cell survival and invasive potential within the TME. (**Supplementary Figure 3B**). To further confirm cell interactions within ecosystems, we used CellChat for analysis of nine different CCs. To avoid errors and overinterpretation, we set interaction.range = 250 and shielded signals at longer distances, focusing only on intra-ecosystem interactions (**Supplementary Figure 3C**). Results for CC4, which has fewer tumor cells, indicated that cell interactions mainly promote tumor migration and Extracellular matrix (ECM) remodeling (**Figure 3B**). Tumor invasion is mainly mediated by ligands such as VEGF, COMP, ANGPTL2, and CEACAM6, which promote angiogenesis, inhibit immune responses, and reduce tumor cell adhesion, facilitating infiltration (50, 51). Specifically, VEGF activating FLT1 (VEGFR1) reduces T cell infiltration and cytotoxic immune responses (52); COMP expression aids the infiltration of M2 macrophages and fibroblasts; CEACAM6 and ANGPTL2 promote tumor infiltration by decreasing cell adhesion and downregulating MHC-I expression. Some studies suggest that stromal fibrosis promotes tumor infiltration (53), and ECM remodeling in CC4 mainly operates through collagen cross-linking and integrin pathways, which facilitate fibrosis (54, 55). Notably, COL4A1 promotes tumor migration via FAK-Src signaling, and its expression is associated with poor prognosis (56, 57).

Another infiltrating ecosystem, CC6, also primarily involves immunosuppression and ECM remodeling, but with a different regulatory network (**Figure 3C**). Besides collagen-integrin signaling, CD44 plays an important role in matrix reprogramming; activation of CD44 via Nanog signaling maintains stem cell properties (58). SDC1 serves as a central node in CC6 metabolic network; as a heparan sulfate proteoglycan, its high expression is associated with poor tumor prognosis (59). Its activation increases tumor migration, directly aiding infiltration (60, 61); it can also be activated by COL1A1, exacerbating fibrosis (62). Many previous studies have linked increased extratumoral matrix rigidity with worse prognosis (63). From the perspective of ecological niche integration with cellular interactions, we re-examine this process, especially emphasizing the crucial functions of SDC1 at the edges of tumor infiltration. It may act as a bridge in tumor invasion and ECM formation. Overall, the internal interactions of CC4 and CC6 provide new insights into mechanisms of immune evasion and metastasis in infiltrating tumors, as well as the unique pro-fibrotic role of myCAFs in ECM.

To investigate the potential progression from the tumor core to the invasive margin, we constructed a spatial trajectory from CC2 through CC6 to CC4 based on SPATA2, originating from CC2, passing through CC6, and ending at CC4, to compare spatial changes in tumor ecosystem development (**Figure 3D**). Genes along this trajectory were subjected to HALLMARK pathway enrichment; results showed that pathway enrichment perfectly followed the trajectory changes, with CC6 exhibiting overexpression of gene sets characteristic of CC2 and CC4 (**Figure 3E**). Compared to CC6, CC2 upregulated pathways mainly involved MYC_TARGETS and oxidative phosphorylation, indicating vigorous proliferative capacity. Conversely, anti-tumor pathways such as inflammation, interferon, antigen presentation, P53, complement, and apoptosis were almost uniformly under-expressed, consistent with the immune desert landscape characterized by sparse immune cells. CC4 specifically upregulated pathways related to angiogenesis, epithelial-mesenchymal transition (EMT), and myofiber formation, aligning with typical infiltrating tumor features (64), consistent with cell interaction results. We further examined the spatial expression patterns of ligands and receptors critical in cell communication within CellChat (**Figures 3F, G**). The results were intriguing: SDC1 and ITGA2 are highly expressed specifically in the tumor microenvironment; COMP is specifically distributed at the tumor margins. COMP is considered related to immune suppression, and some studies suggest that activation of COMP promotes EMT (65). Interestingly, in CC4, COMP acts as a ligand interacting with SDC1, indicating it may play a crucial role in tumor infiltration and progression (66). Given SDC1’s central role in communication within CC4, inhibiting their interaction might hinder tumor infiltration.

In summary, our analysis delineates a spatial continuum from the proliferative, immune-desert tumor core (CC2) to the invasive margin (CC4), which is characterized by EMT, angiogenesis, and specific ligand-receptor interactions such as COMP-SDC1. However, immune cells are impeded by fibroblast-formed fibrous barriers and a series of immunosuppressive mechanisms at the tumor margin. These immune cells cannot enter the tumor center to exert anti-tumor effects, while infiltrating tumors can generate new blood vessels and continue expanding via mechanisms like EMT. Therefore, finding therapeutic strategies targeting the tumor margin microenvironment might greatly enhance anti-tumor efficacy. We propose that inhibiting SDC1 and COMP could be potential therapeutic approaches. In conclusion, by dissecting different tumor components and integrating spatial interactions and trajectories, we offer a new perspective on the infiltration characteristics of the tumor microenvironment.

### CYP27A1^+^TAMs and CD8^+^T cells co-localization as a key factor in immunotherapy success

The stroma in the TIME also plays a critical role in determining therapeutic efficacy (67). To investigate the key cellular communities (CCs) influencing immunotherapy outcomes, we compared the composition of CCs between different treatment response groups (**Figure 2B**). To evaluate the independent predictive value of CC9, we performed multivariate logistic regression analysis in the IMvigor210 cohort. The CC9 signature score for each sample, quantified using ssGSEA, was incorporated into the model along with tumor stage and Immune Phenotype. The analysis confirmed that a higher CC9 signature score was significantly associated with increased likelihood of treatment response, identifying it as an independent favorable factor(**Supplementary Figure 3D**). These results suggest that deconstructing CC9 may help uncover the mechanisms underlying differential immunotherapy responses.

In the UMAP visualization, CC9 and CC1 were closely distributed, and both were predominantly located in the stromal region. Additionally, the proportions of cell types within these two communities were generally similar. However, CC9 exhibited a significantly higher proportion of CD8^+^T cells than CC1 (**Figure 3A**). To dissect the cellular basis for this differential T cell abundance and explore the potential multi-cellular cooperation within CC9, we performed GSVA to score the activity of cytotoxic T lymphocytes (CTLs), as well as the presence of TLS and other key cell types within this community (**Supplementary Figure 4**). Subsequent correlation analysis revealed a strong positive association between CYP27A1^+^TAMs and CTL activity. In contrast, SPP1^+^TAMs and myCAFs showed the expected negative correlations. Notably, the TLS signature displayed the strongest positive correlation with CTL activity. Significant positive correlations were also observed between CTL activity and signatures for B cells and iCAFs. These findings collectively suggest that within CC9, multiple stromal and immune components—including TLS, B cells, iCAFs, and CYP27A1^+^TAMs—likely act in concert to foster an anti-tumor microenvironment, constituting a multi-cellular cooperative immune niche. Given its strongest correlation with CTLs among individual cells (**Supplementary Figure 4**) and its marked enrichment in CC9 versus CC1 (**Figure 2C**), we focused onCYP27A1^+^TAMs.

**Figure 4:**
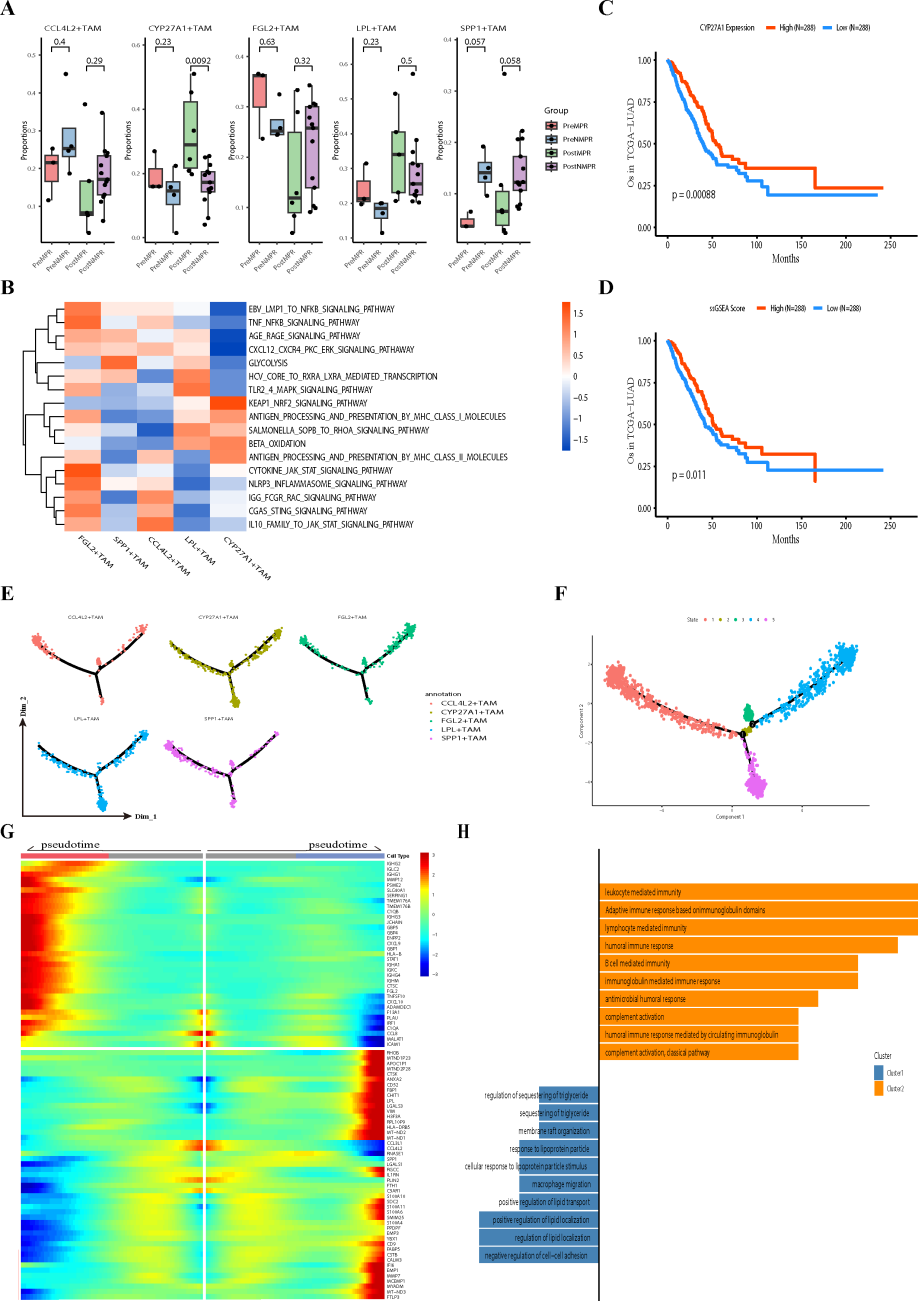
The tumor niche uncovers key cells and genes involved in tumor progression **(A)**. Box plots illustrating the proportions of various macrophage subtypes in single-cell samples before and after immunochemotherapy, as well as under different pathological response conditions. (Wilcoxon test) **(B)** Heatmap of genesets from KEGG for scaled GSVA across macrophages. **(C)**. In the TCGA_LUAD dataset, patients were divided into high and low CYP27A1 expression groups based on the median expression level. The OS of patients in these two groups was then compared. OS, overall survival **(D)** The difference in the overall survival rates between patients with high and low CYP27A1^+^TAMs scores (log-rank test). **(E)** Display the distribution of different macrophage cell states among pCR patients using Monocle 2. **(F)** In pCR patients, macrophage state calculated by monocle2. **(G)** Heatmap showing gene expression changes across two clusters during pseudotime progression, with clusters annotated on the left. **(H)** Gene Ontology (GO) enrichment analysis of differentially expressed genes across clusters.

Strikingly, the Post-MPR group showed a significantly higher number of CYP27A1^+^TAMs than the Post-NMPR group (**Figures 4A**), implying that CYP27A1^+^TAMs may play a crucial role in immunotherapy. To further investigate their functional significance, we performed GSVA on macrophages using key KEGG gene sets (**Figure 4B**). As expected, FGL2^+^TAMs exhibited M1-like properties, demonstrating strong pro-inflammatory and cytotoxic capabilities, along with antigen presentation. In contrast, SPP1^+^TAMs showed minimal pro-inflammatory and antigen-presenting functions, consistent with previous findings (68). However, the tumor microenvironment is more complex than previously thought, and understanding TAMs solely from a pro-/anti-inflammatory perspective is insufficient (7, 69). Interestingly, although CYP27A1^+^TAMs did not express traditional M1 macrophages markers, they exhibited the highest expression of MHC pathway genes. Moreover, in the TCGA-LUAD cohort, patients with high expression of CYP27A1 and CYP27A1^+^TAMs had significantly better prognosis than those with low expression (**Figures 4C, D**).

**Figure 5:**
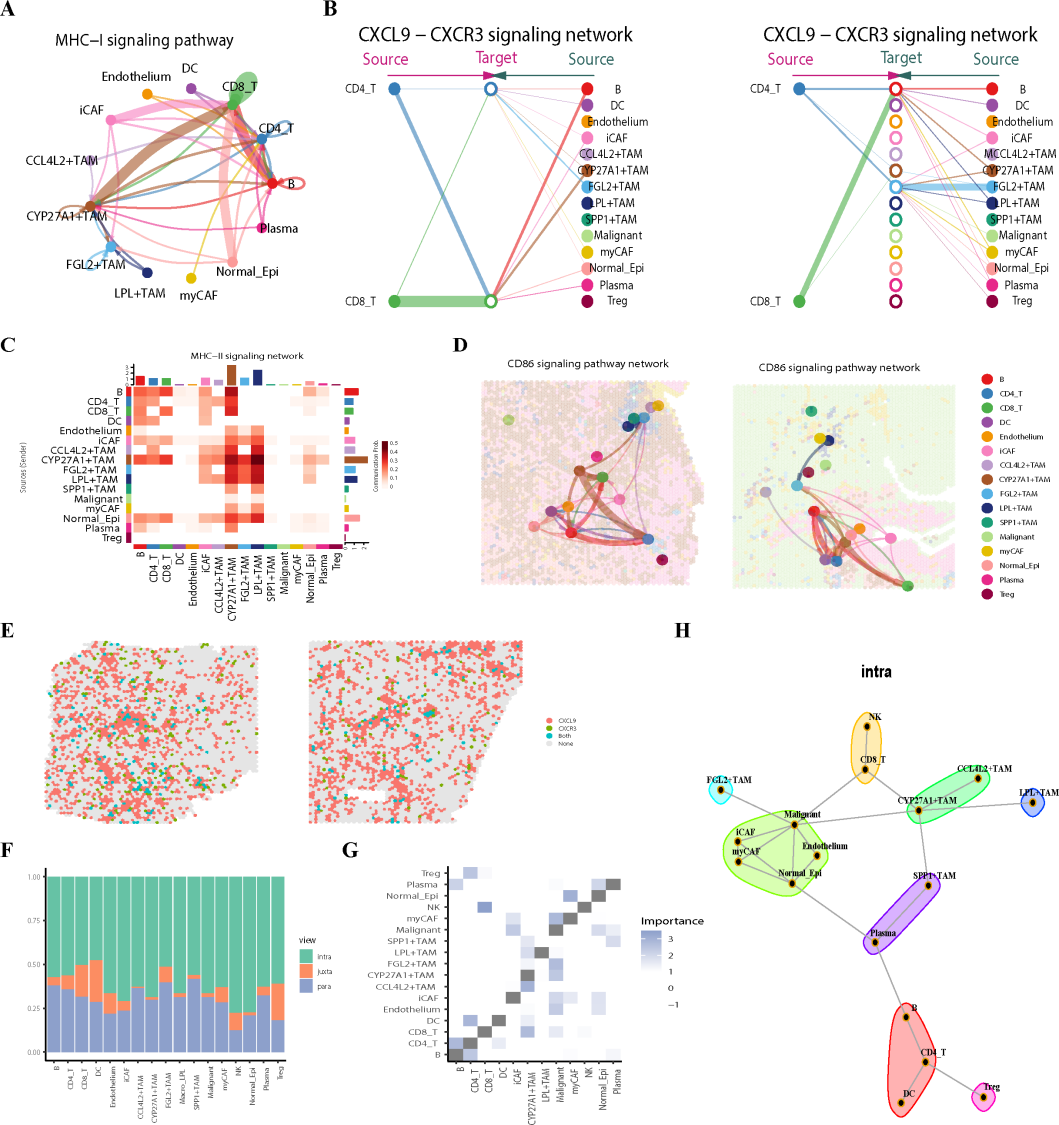
Cell communication and spatial localization reveal that CYP27A1^+^TAMs function through T cell recruitment **(A)**. Circle plot displaying cell-cell interactions via the MHC class I signaling pathway in ST samples. **(B)** Hierarchy plot illustrating cell-cell interactions of CXCL9-CXCL3 receptors and ligands in ST samples. **(C)** Heatmap showing cell-cell interactions of the MHC class II signaling pathway in ST samples. **(D)** Spatial interaction plot demonstrating CD86 signaling pathway interactions in S_MPR_1 and T_I_1 samples. **(E)** Spatial feature plot depicting the localization and co-localization of CXCL9-CXCL3 receptors and ligands within spots. **(F)** Stacked barplot showing the average view contribution fraction per target across all samples from the Misty cell type co-localization analysis, stratified by perspective (intra, juxta,para). **(G)** Co-localization strength heatmap of Misty cell types in the intra perspective, with deeper colors indicating stronger co-localization signals. **(H)** Co-localization strength schematic for Misty cell types in the intra perspective, with a cutoff threshold set at 1.5.

We then utilized Monocle2 to analyze macrophages functional dynamics from a developmental perspective, focusing on patients with pCR (**Figure 4E**). The results showed that CYP27A1^+^TAMs and LPL^+^TAMs had similar state distributions and were almost mutually exclusive with SPP1^+^TAMs, suggesting that CYP27A1^+^TAMs may represent a macrophage subset with dual roles in lipid metabolism and tumor suppression within the TIME. Next, we examined macrophages developmental trajectories by analyzing gene expression differences at branching point 1, which revealed two distinct clusters (**Figures 4F, G**). Functional enrichment analysis of these clusters showed that BP terms were primarily associated with two major categories: cluster 1 was enriched in lipid metabolism-related processes, while cluster 2 was associated with adaptive immune responses (**Figure 4H**). Based on the state distributions of CYP27A1^+^TAMs and FGL2^+^TAMs, we propose that CYP27A1^+^TAMs are a macrophage subset with dual functions in immune response and lipid metabolism regulation. Here, we first rigorously assessed the specificity of CYP27A1 to ensure it serves as a unique marker for CYP27A1^+^TAMs (**Supplementary Figure 5A**). Since traditional alveolar macrophages are known to possess both immune and lipid metabolic functions, we further compared the expression of established alveolar macrophage markers across several macrophage subtypes. This analysis confirmed that CYP27A1^+^TAMs are distinct from alveolar macrophages (**Supplementary Figure 5B**). Having established this distinction, we proceeded to analyze the characteristics of CYP27A1^+^TAMs. Given that CYP27A1^+^TAMs exhibited the strongest antigen-presenting capacity, we hypothesized that they may primarily exert their immune functions through antigen presentation and T cell activation. To validate whether CYP27A1^+^TAMs interact with T cells, we performed cell communication analysis. Since spatial-level CellChat accounts for actual spatial distances and provides more realistic interaction insights, we applied it to spatial transcriptomics samples. The results were consistent with single-cell data: CYP27A1^+^TAMs played a critical role in antigen presentation to T cells, particularly CD8^+^T cells, via both MHC-I and MHC-II pathways (**Figures 5A, C**).Previous studies have suggested that after PD-1 immunotherapy, macrophages interact with CD8^+^T cells via CXCL9/CXCR3 to enhance T cell recruitment and improve immunotherapy efficacy (70). We therefore examined whether CXCL9/CXCR3 were highly expressed in CYP27A1^+^TAMs and validated their co-localization in spatial samples. The results confirmed that CYP27A1^+^TAMs specifically highly expressed CXCL9/CXCR3 (**Figure 5B**). More importantly, CYP27A1^+^TAMs were spatially co-localized with CD8^+^T cells (**Supplementary Figures 5C, D**), and the spatial patterns of CXCL9/CXCR3 significantly overlapped with this co-localization (**Figure 5E**). Given the important role of CD86 in T cell activation and functional differentiation, we analyzed CD86 interaction patterns. At a significance threshold of p = 0.01, CYP27A1^+^TAMs dominated CD86 interactions, extensively activating CD8^+^T and CD4^+^T cells but not Tregs (**Figure 5D**; **Supplementary Figures 5E, F**). These CellChat results indicate that CYP27A1^+^TAMs recruit and activate T cells, especially CD8^+^T cells, through multiple pathways, which may be the primary mechanism underlying their anti-tumor effects.

To investigate whether the interaction between CYP27A1^+^TAMs and CD8^+^T cells is widespread, we used the spatstat package and calculated their co-localization in each sample based on Ripley’s K function method. The results indicated that in every sample, the tendency for co-localization between the two far exceeded random distribution, and both the Aggregation Index and Cohen’s d were highly significant(**Supplementary Figure 6**). Then we performed multi-view spatial co-localization analysis using MISTY, a machine learning-based framework (24). MISTY employs interpretable machine learning algorithms to quantify the co-localization patterns of cells or genes across multiple spatial transcriptomics samples within defined spatial contexts. The definitions of the three distance views were adapted from the method described by Ateeq M. Khaliq et al. (47): the intra-view (55 µm, within a spot), the juxta-view (200 µm, capturing direct and short−range interactions), and the para-view (3000 µm, assessing long−range or tissue−level organization). The para−view excluded co−localization within 200 µm to avoid overlap with the juxta−view (**Figure 5F**). Using a cutoff value of 1.5, tumor cells, iCAFs, and CYP27A1^+^TAMs emerged as core players in cellular co-localization, playing crucial roles (**Figures 5G, H**). These results further support the co-localization between CYP27A1^+^TAMs and CD8^+^T cells, and their joint co-localization with tumor cells highlights the indispensable role of CYP27A1^+^TAMs in the anti-tumor microenvironment. Notably, although the number and pattern of spatial co-localizations changed under juxta-view and para-view, CYP27A1^+^TAMs consistently maintained a relationship with CD8^+^T cells, demonstrating robust spatial dependency (**Supplementary Figures 5G–J**). Additionally, cells of the same type tended to co-localize, reflecting harmonious spatial organization.

Integrating these findings, we compared the reasons for differences in immunotherapy efficacy and identified CYP27A1^+^TAMs as a key factor. These TAMs exhibit characteristics distinct from traditional M1 macrophages and play a critical anti-tumor role in the TIME by recruiting CD8^+^T cells.

### Immunotherapy enhances the function of CYP27A1^+^TAMs through upregulation of LXR

**Figure 6:**
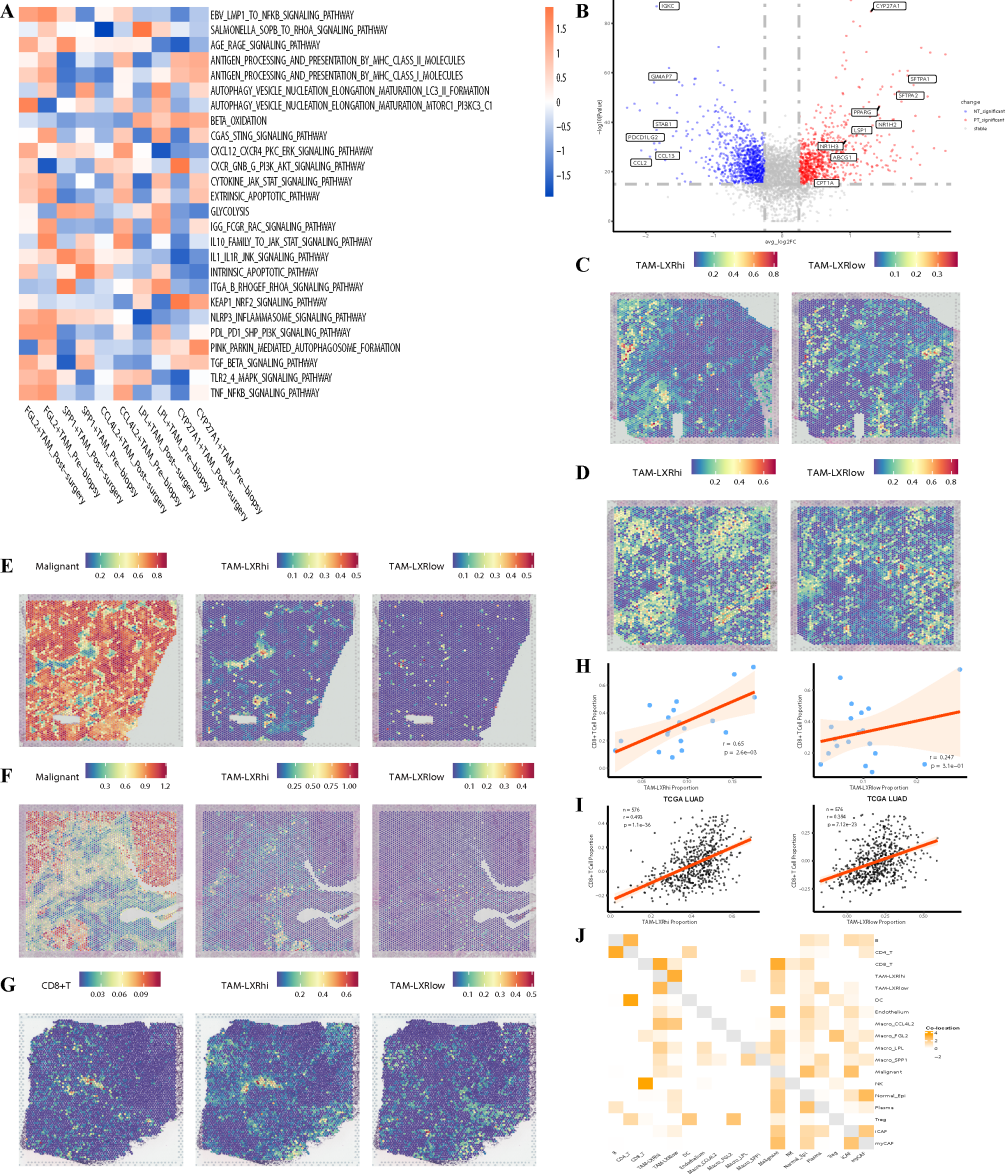
Immunotherapy enhances the function of CYP27A1^+^TAMs through LXR upregulation **(A)**. Heatmap of genesets from KEGG for scaled GSVA across macrophages, with samples grouped into pre-treatment and post-treatment conditions. **(B)** Volcano plot displaying differentially expressed genes in CYP27A1^+^TAMs between non-treatment (NT) and post-treatment (PT) conditions. **(C, D)** Spatial distribution of CYP27A1^+^TAMs with high vs. low LXR expression in stromal samples. **(E, F)** Distribution of tumor cells and CYP27A1^+^TAMs with high vs. low LXR expression in T_C and T_I samples. **(G)** Distribution of CD8^+^T cells and CYP27A1^+^TAMs with high vs. low LXR expression in stromal samples. **(H)** Correlation between CD8^+^T cells and CYP27A1^+^TAMs with high vs. low LXR expression in single-cell samples (Pearson’s correlation test). **(I)** Correlation between CD8^+^T cells and CYP27A1^+^TAMs with high vs. low LXR expression in TCGA LUAD cohort (Pearson’s correlation test). **(J)** Heatmap showing co-localization intensity of different cell types within spots (intra view); darker colors indicate higher co-localization intensity.

To further investigate the impact of immunotherapy on the regulation of CYP27A1 expression, we performed GSVA on CYP27A1^+^TAMs in different states. Surprisingly, the antigen presentation capacity did not increase after immunotherapy (**Figure 6A**). To explore this discrepancy, we compared the differentially expressed genes in CYP27A1^+^TAMs before and after immunotherapy. The results showed significant upregulation of CYP27A1, LXR, and its target genes (71) post-treatment (**Figure 6B**). Additionally, the expression of SFTPA1 and SFTPA2 was markedly increased. Previous studies suggest that high expression of SFTPA1 and SFTPA2 in macrophages enhances their survival and resistance to apoptosis (72). In line with these findings, following combined chemoimmunotherapy, various apoptosis pathways were downregulated in CYP27A1^+^TAMs, while pathways such as CXCR GNB G PI3K AKT SIGNALING PATHWAY and KEAP1 NRF2 SIGNALING PATHWAY (73, 74) were significantly upregulated. These pathways promote cell survival and enhance resistance to oxidative stress, which may be key to the sustained anti-tumor role of CYP27A1^+^TAMs in patients receiving combination therapy. LXR (Liver X Receptor α and β), nuclear hormone receptors encoded by NR1H2 and NR1H3, are ligand-activated transcription factors specifically expressed in the liver and macrophages (75). Recently, the team of Srustidhar Das demonstrated that LXR has anti-tumor effects mediated through the recruitment of B cells, T cells, and the formation of tertiary lymphoid structures. They also identified CYP27A1 as the most important agonist of LXR (76). Interestingly, in our study, both ligands and receptors of LXR were significantly upregulated in CYP27A1^+^TAMs after immunotherapy. This led us to investigate whether high LXR expression enhances the ability of CYP27A1^+^TAMs to recruit T cells.

We divided CYP27A1^+^TAMs into two groups based on LXR expression levels: TAMs-LXRhi and TAMs-LXRlow. Using RCTD for re-deconvolution and annotation of spatial transcriptomics samples, we found that the distribution of TAMs-LXRhi and TAMs-LXRlow in the stroma showed no significant difference (**Figures 6C, D**). However, within tumor samples, TAMs-LXRhi was significantly more abundant than TAMs-LXRlow (**Figures 6E, F**). Moreover, we observed that TAMs-LXRhi exhibited stronger co-localization with CD8^+^T cells (**Figure 6G**). Compared to TAMs-LXRlow, TAMs-LXRhi displayed a more activated phenotype, suggesting that LXR activation may enhance the function of CYP27A1^+^TAMs. To broadly validate this observation across all samples, we used MISTY to compare the strength of co-localization between CYP27A1^+^TAMs and T cells under different LXR expression levels (**Figure 6J**). Furthermore, we validated the correlation between LXR expression in CYP27A1^+^TAMs and CD8^+^T cells in both single-cell and TCGA cohorts (**Figures 6H, I**). The results confirmed that TAMs-LXRhi had significantly stronger co-localization with T cells than TAMs-LXRlow, indicating that high LXR expression is associated with enhanced T cell recruitment ability. To identify the downstream mediators responsible for LXR-driven T cell recruitment, we analyzed the correlation between canonical LXR target genes (including APOE, ABCG1, and ABCA1) (77) and CD8^+^T cell markers (CD8A and CD8B) in the TCGA-LUAD dataset. This analysis revealed significant positive correlations between these LXR targets and CD8^+^T cell abundance (**Supplementary Figure 7**), with APOE showing the most pronounced association. This suggests APOE may be a pivotal LXR-induced factor enhancing the T cell recruitment capacity of these macrophages. Therefore, our data indicate that APOE, among other LXR-induced factors, may play a pivotal role in enhancing the CD8^+^T cell recruitment capacity of CYP27A1^+^TAMs following LXR activation.

In summary, by comparing the genetic and pathway changes in CYP27A1^+^TAMs before and after treatment, we found that anti-apoptotic capacity and LXR expression were significantly increased post-treatment. TAMs-LXRhi represents an activated state of CYP27A1^+^TAMs with stronger survival ability and enhanced CD8^+^T cell recruitment capacity.

### Validation with external cohorts

**Figure 7:**
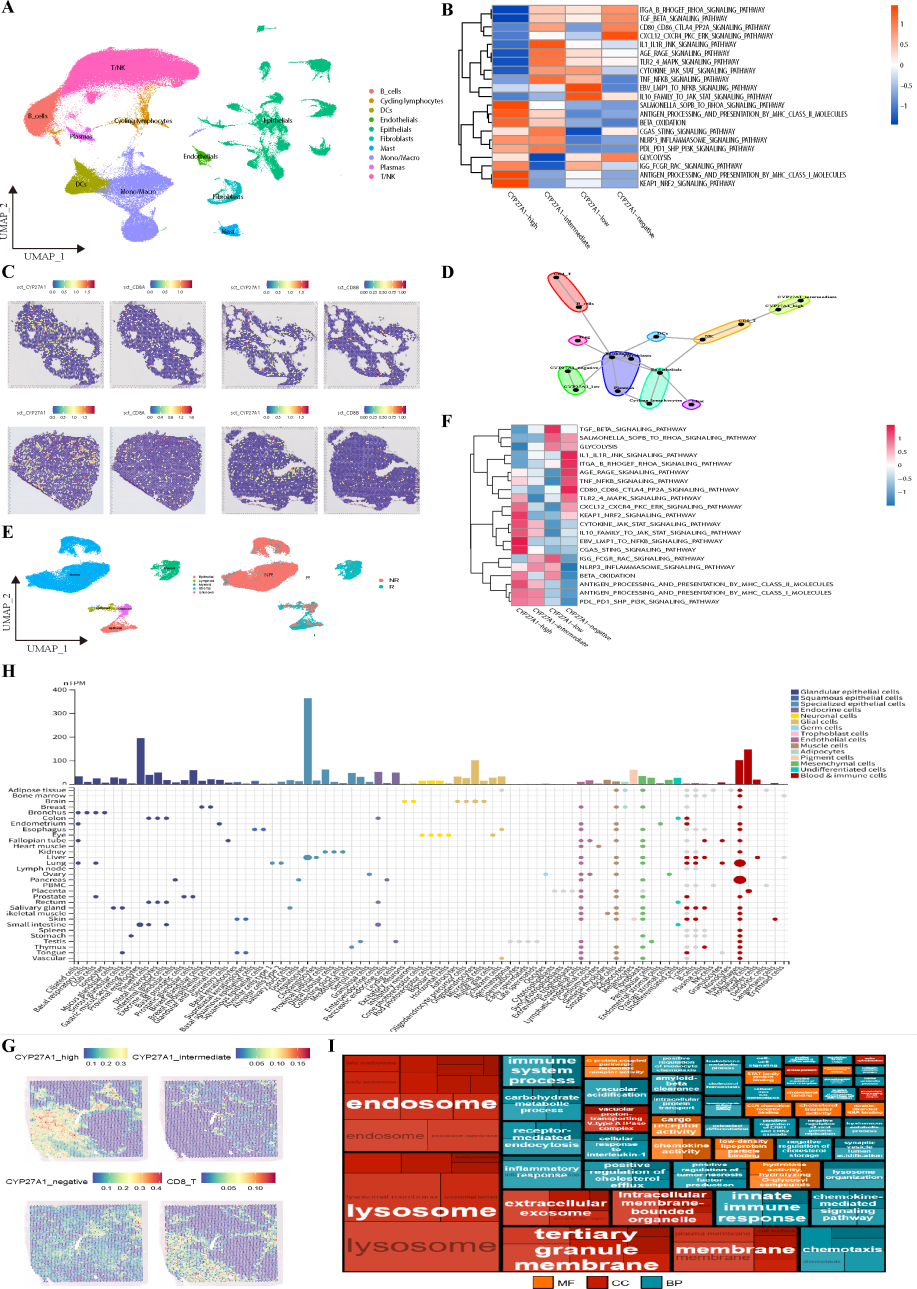
External Cohort Validation of CYP27A1^+^TAMs Function **(A)**. UMAP plot of 201,003 cells from the single-cell cohort GSE131907. **(B)** Heatmap of genesets from KEGG for scaled GSVA across macrophages ranked by CYP27A1 expression in GSE131907. **(C)** Spatial feature plot showing the spatial localization of CD8A, CD8B, and CYP27A1 genes in an E-MTAB-13530 sample. Gene expression values were normalized using SCTransform. **(D)** Schematic of co-localization strength for Misty cell types from an intra-perspective in E-MTAB-13530, with a cutoff threshold set at 1.5. **(E)** UMAP plot displaying major cell types and treatment response for 33,443 cells from GSE233203. R: responder, NR: non-responder. **(F)** Heatmap of genesets from KEGG for scaled GSVA across macrophages ranked by CYP27A1 expression in GSE223203. **(G)** Spatial feature plot illustrating the spatial distribution of different cell types in a sample from GSE267960. **(H)** Bubble plot showing the distribution of CYP27A1 gene expression across various tissues and cell types based on data from the Human Protein Atlas. **(I)** Dendrogram displaying GO pathways enriched with specific genes from cluster 58.

To assess the generalizability of our findings, we performed validation across multiple independent external datasets. The first validation dataset was GSE131907. After quality control (**Supplementary Figure 5A**), dimensionality reduction, clustering, and batch effect removal (**Supplementary Figure 5B**), we identified 10 major clusters (**Figure 7A**; **Supplementary Figure 5C**). Consistent with our own data, CYP27A1 was specifically highly expressed in macrophages, and NR1H2 and NR1H3 showed strong correlation with CYP27A1 expression levels (**Supplementary Figure 5D**). To assess the impact of CYP27A1 expression levels on macrophage functional states, we divided mononuclear phagocytes into quartiles based on CYP27A1 expression (**Supplementary Figures 5E, F**). Notably, macrophages in lung tissue exhibited higher overall CYP27A1 expression compared to those in pleural effusion, brain tissue, and lymph nodes. The groups were named, from highest to lowest expression: CYP27A1-high, CYP27A1-intermediate, CYP27A1-low, and CYP27A1-negative. We then scored their functional states using KEGG pathways (**Figure 7B**). Mirroring our internal results, the CYP27A1-high group demonstrated the strongest antigen presentation capacity for both MHC-I and MHC-II pathways and a greater reliance on β-oxidation rather than glycolysis at the metabolic level. Subsequently, using the annotations from GSE131907, we deconvoluted and annotated 57,238 spots from a spatial transcriptomics dataset (E-MTAB-13530), comprising lung adenocarcinoma and normal tissue. The results revealed a high spatial correlation between CYP27A1 and CD8A/CD8B (**Figure 7C**; **Supplementary Figures 5G, H**). We further employed MISTY to quantify co-localization differences between cell types across all samples, which again confirmed that macrophages with high CYP27A1 expression are more likely to co-localize with CD8^+^T cells in space (**Figure 7D**).

The second validation cohort’s single-cell data came from GSE223203. After quality control and batch effect removal (**Supplementary Figures 6A, B**), we applied the same clustering method as the original publication (**Figure 7E**; **Supplementary Figure 6C**). CYP27A1 expression was remarkably consistent across datasets, specifically appearing in myeloid cells and even serving as a potential marker for this lineage. Cluster 8, lacking specific markers, was ultimately defined as ‘Unknown’. For macrophages within the myeloid cell population, we applied the same quartile-based grouping (**Supplementary Figure 6D**). Notably, non-responder (NR) patients had a relatively higher proportion of macrophages in the CYP27A1-negative group, suggesting that low CYP27A1 expression in macrophages may be associated with resistance to immunotherapy. Subsequent KEGG pathway analysis of the quartile-grouped macrophages (**Figure 7F**) showed that the CYP27A1-high group remained the primary contributor to antigen presentation and preferred β-oxidation for energy production. Considering the presence of ‘Unknown’ cells in GSE223203, we used GSE131907 to deconvolute and annotate another spatial dataset, GSE267960 (two samples, 9401 spots). Both at the gene and cell type level, CYP27A1 and CD8^+^T cells were in close spatial proximity (**Figure 7G**; **Supplementary Figure 6E**).

Most importantly, our findings were strongly corroborated by the Human Protein Atlas. First, we examined the organ and cellular distribution of CYP27A1 (**Figure 7H**), which confirmed prior reports of its high expression in hepatocytes (78). In the lung, CYP27A1 was specifically highly expressed in macrophages. Furthermore, only macrophages in the lung and pancreas highly expressed CYP27A1; this dual specificity makes CYP27A1^+^TAMs easier to identify and the results more reliable. In the Human Protein Atlas single-cell clustering, CYP27A1 is part of cluster 58 (Macrophages - Innate immune response) with a confidence score of 1 (**Supplementary Figure 6F**). (Confidence is the fraction of times a gene was assigned to the cluster in repeated clustering, and therefore reflects how strongly associated it is to the cluster. A confidence of 1 indicates that the gene was assigned to this cluster in all repeated clusters.) Cluster 58, Macrophages - Innate immune response, specifically highly expresses 420 genes (**Supplementary Materials 3**). Using these 420 genes, GSVA and Jaccard similarity scores were calculated for each cluster in the single-cell dataset. Both analyses showed that the highest scores were observed in the CYP27A1^+^macrophage cluster. (**Supplementary Figures 6G, H**), suggesting very similar functional states despite different microenvironments.

Examining the GO enrichment results for Cluster 58 (**Figure 7I**), its biological processes are primarily focused on innate immune response—namely, non-specific immune functions like phagocytosis and antigen presentation. Cellular component analysis reveals exceptionally well-developed lysosomes and endosomes, consistent with active phagocytic activity. Given that lysosomes are crucial for antigen processing and presentation in antigen-presenting cells, their proper function is essential for activating anti-tumor T cell immunity. Furthermore, its molecular functions are enriched in pattern recognition receptors and lipid metabolism, mirroring the characteristics of CYP27A1^+^TAMs in our study. This underscores the stability of CYP27A1 as a marker for a specific macrophage subset within the TIME that exerts anti-tumor effects by recruiting T cells.

In summary, through two external single-cell datasets, two spatial transcriptomics datasets, and integration with the extensive single-cell atlas from the Human Protein Atlas, we firmly establish that CYP27A1 is specifically highly expressed in lung macrophages and demonstrates a robust correlation with strong antigen presentation capacity.

### Construction and independent validation of a prognostic model based on the CYP27A1^+^ macrophage risk score

To translate our findings into a clinically applicable tool, we developed a prognostic model based on the CYP27A1^+^TAMs gene signature. Given that high expression of genes associated with CYP27A1^+^TAMs correlates with improved patient survival (**Figures 4C, D**), we sought to develop a gene expression-weighted prognostic model. This model, designated the CYP27A1^+^Macrophage Risk Score (CMRS), stratifies patients into distinct risk groups.

**Figure 8:**
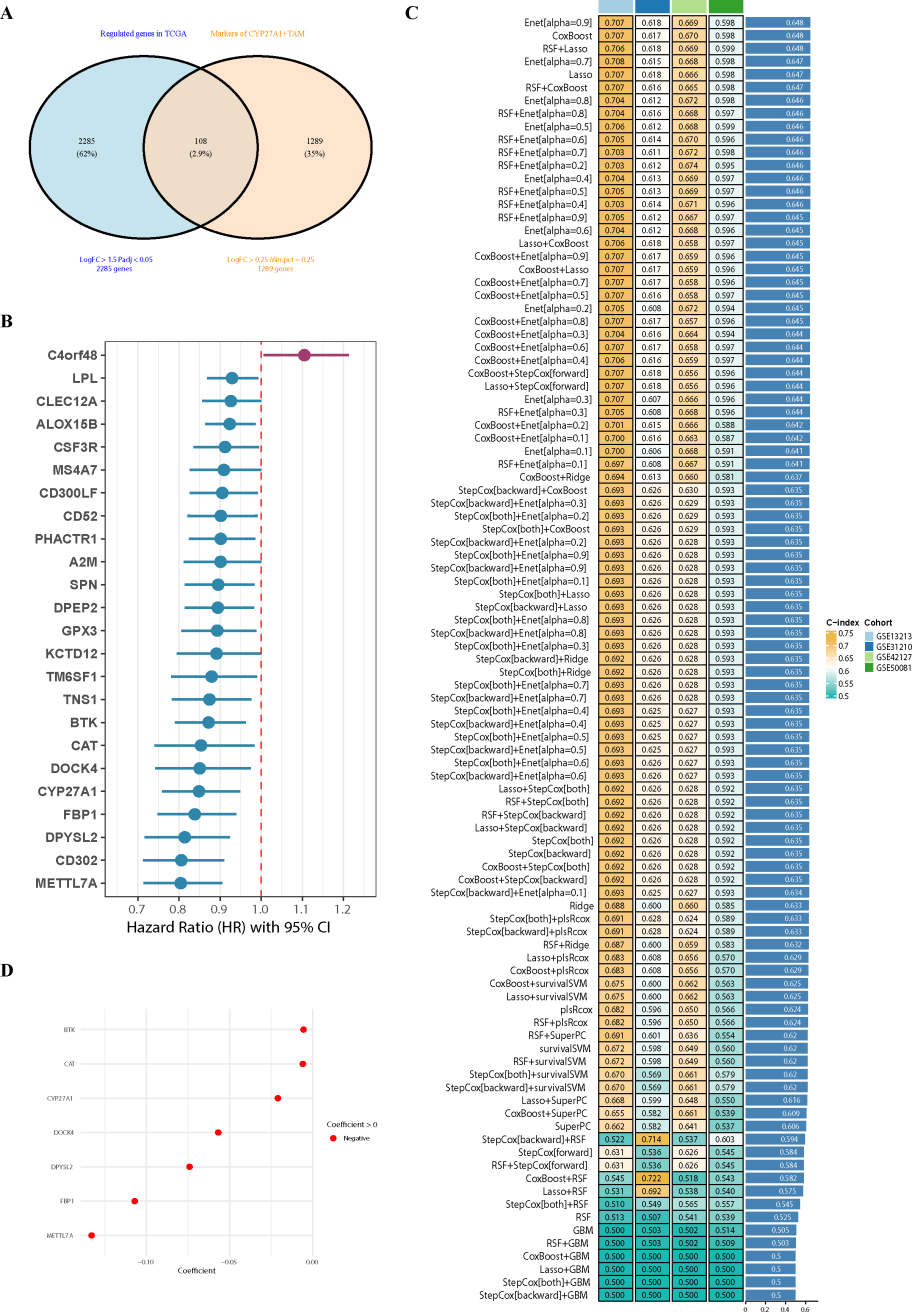
Construction of CMRS model through 101 machine learning approaches **(A)**. Venn diagram showing the intersection of differentially expressed genes in TCGA-LUAD and markers of CYP27A1^+^TAMs. **(B)** Forest plot of 24 genes significantly associated with prognosis, identified by univariate Cox regression analysis. **(C)** Heatmap comparing the predictive performance (C-index) of 101 machine learning model combinations—including RSF, GBM, LASSO, CoxBoost, and others—across multiple datasets. **(D)** Bubble plot showing the COEF values of different genes in CMRS.

Our analysis identified 2,285 and 1,289 differentially expressed genes from the TCGA-LUAD dataset and CYP27A1^+^TAMs, respectively. The intersection of these gene sets yielded 108 candidate genes (**Figure 8A**). Subsequent univariate Cox regression analysis of these genes within the TCGA-LUAD cohort identified the top 24 genes most significantly associated with prognosis (**Figure 8B**), which were utilized for model construction. Notably, among these 24 prognostically significant genes, only one was identified as a hazard factor, underscoring the predominant protective role and robust prognostic predictive value of CYP27A1^+^TAMs.

For model development, the TCGA-LUAD cohort served as the training set, while the GSE13213, GSE31210, GSE42127, and GSE50081 datasets were employed as independent validation cohorts. By systematically evaluating 101 distinct machine learning algorithm combinations, we developed a robust predictive model. The predictive performance of each model was assessed by calculating the average C-index across all cohorts (**Figure 8C**). Our analysis identified the Elastic Net algorithm with an alpha of 0.9 as the optimal model, demonstrating superior integrated performance across the TCGA-LUAD, GSE13213, GSE31210, GSE42127, and GSE50081 cohorts, with a mean C-index of 0.648. Consequently, this algorithm was selected to construct the final CMRS scoring system, which incorporates 7 protective genes. The relative feature importance within the model is depicted in **Figure 8D**.

**Figure 9:**
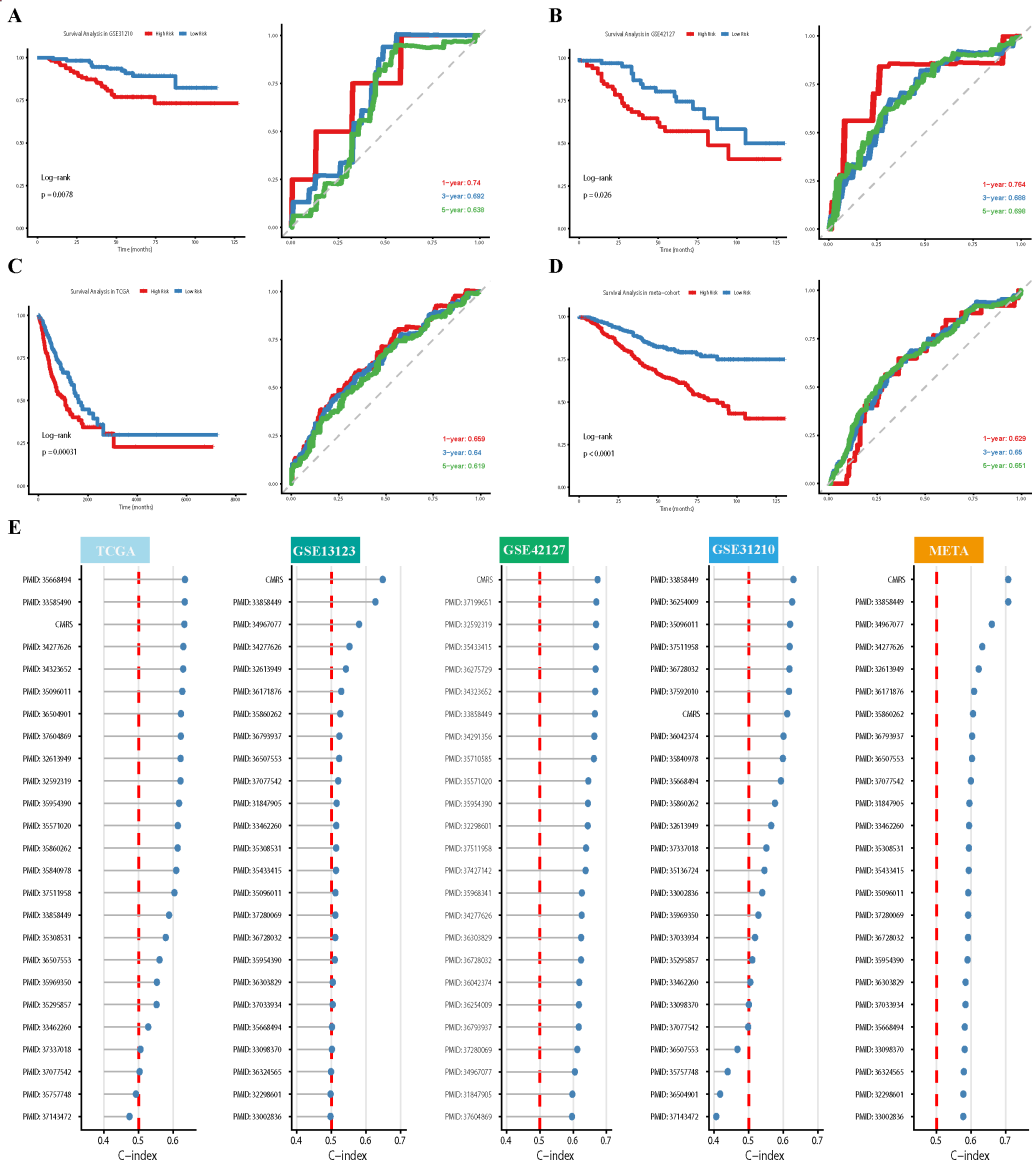
Prognostic evaluation and external validation of the CMRS model **(A–D)**. Kaplan-Meier survival analysis and receiver operating characteristic (ROC) curve analysis were performed to evaluate the association between the CMRS and overall survival (OS), as well as the model’s prognostic predictive performance. These analyses were conducted in the TCGA-LUAD training cohort **(C)**, two external GEO validation cohorts **(A, B)**, and a meta-cohort **(D)** comprised of GSE13213, GSE41217, GSE50081, and GSE31210. **(E)** Comparison of the C-index between the CMRS and previously published models across the training and testing datasets.CMRS, CYP27A1^+^Macrophage Risk Score; ROC, receiver operating characteristic; OS, overall survival.

We first compared the performance of CMRS against previously published models by evaluating their C-indices across different cohorts. The results demonstrated that CMRS consistently achieved superior C-index values in both the training and all validation sets (**Figure 9E**). To further evaluate the prognostic significance of the CMRS, Kaplan-Meier survival analysis was performed. Patients in each cohort (including a meta-cohort combining GSE13213, GSE31210, GSE42127, and GSE50081) were stratified into high-risk and low-risk groups based on the median CMRS score. The analysis revealed that the low-risk group exhibited significantly more favorable overall survival compared to the high-risk group in every cohort. Furthermore, time-dependent receiver operating characteristic (ROC) analysis for 1-, 3-, and 5-year survival confirmed the model’s predictive accuracy against actual observed outcomes (**Figures 9A–D**). These consistent findings across multiple independent datasets support the robustness, generalizability, and reproducibility of the CMRS across diverse clinical populations.

To further evaluate the robustness of the CMRS and address potential concerns regarding overfitting, we conducted comprehensive stability and calibration analyses. First, bootstrap resampling (n=1000) within the TCGA-LUAD training cohort was performed to assess the consistency of the C-index, which yielded a stable and tightly distributed C-index (**Supplementary Figure 8A**), indicating good internal stability of the model. Second, we examined the distribution of C-indices obtained from 500 repeated iterations with different random seeds for splitting the training and validation sets (**Supplementary Figure 8B**); the results showed minimal variance, confirming the robustness of our model selection against random data partitioning. Finally, calibration curves for 1-, 3-, and 5-year survival predictions were constructed. The Hosmer-Lemeshow test was applied to statistically evaluate the agreement between predicted and observed survival probabilities at each time point across cohorts. The curves demonstrated that the survival probabilities predicted by CMRS were consistently in close alignment with the actual observed outcomes (**Supplementary Figures 8C, D**), indicating excellent predictive calibration.

In summary, we constructed and validated the CMRS using the TCGA cohort and multiple independent validation sets. This model not only demonstrates performance superior to existing prognostic tools but also provides a robust instrument for prognostic stratification in LUAD, potentially offering a transformative approach for patient risk assessment and treatment planning.

### Animal experiments validate the anti-tumor role of CYP27A1^+^TAMs

To functionally validate the anti-tumor role of CYP27A1^+^TAMs, we performed *in vivo* experiments. To investigate the impact of macrophages with high CYP27A1 expression on tumor growth, we established a C57 mouse model. Briefly, We selected 6–8-week-old C57BL/6 mice and subcutaneously injected them with LLC cells to establish subcutaneous tumors. Two weeks later, RAW from different groups were injected.

**Figure 10:**
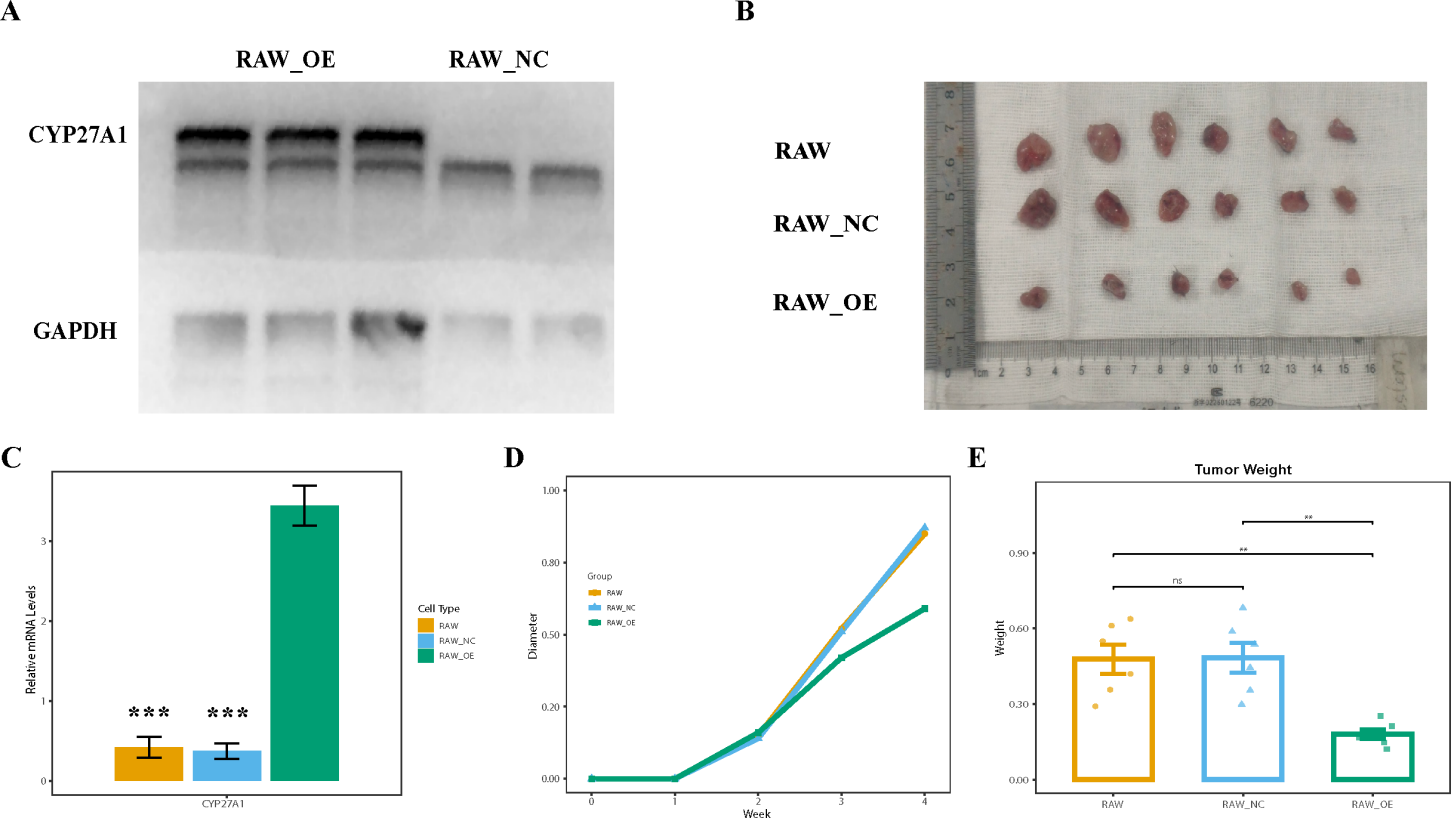
*In vivo* experiments demonstrate that macrophage-specific expression of CYP27A1 suppresses tumor growth **(A)**. Western blot analysis of GAPDH and CYP27A1 protein expression levels in RAW-OE (overexpression) and RAW-NC (negative control) groups. **(B)** Schematic diagram of the subcutaneous tumor formation experiment in C57 mice, modeling the inhibitory effect of CYP27A1 on tumor growth. **(C)** Bar graph showing the transcriptional levels of CYP27A1 in different mouse groups, as detected by qRT-PCR. ***P < 0.001. **(D)** Line chart depicting the change trends in average tumor diameter of mice from different groups at various weeks post-inoculation. **(E)** Box plot comparing the weight of ex vivo tumors from different mouse groups at 4 weeks. **P < 0.01.

First, we measured the transcriptional level of CYP27A1 via PCR. The lentiviral overexpression was successful, resulting in a significant increase in CYP27A1 expression in the RAW-OE group (**Figure 10C**). We then compared differences at the protein level using Western blot (WB), which confirmed that the CYP27A1 protein level in the RAW-OE group was also significantly higher than in the RAW-NC group (**Figure 10A**).

Subsequently, we intravenously administered these three types of macrophages via the tail vein into C57 mice that had borne subcutaneous tumors for two weeks. We then monitored tumor size over four weeks and measured tumor weight at the fourth week. The results demonstrated that RAW-OE significantly inhibited tumor cell growth, indicating that macrophages with high CYP27A1 expression within the TIME indeed exert an anti-tumor effect (**Figures 10B, D, E**).

**Figure 11:**
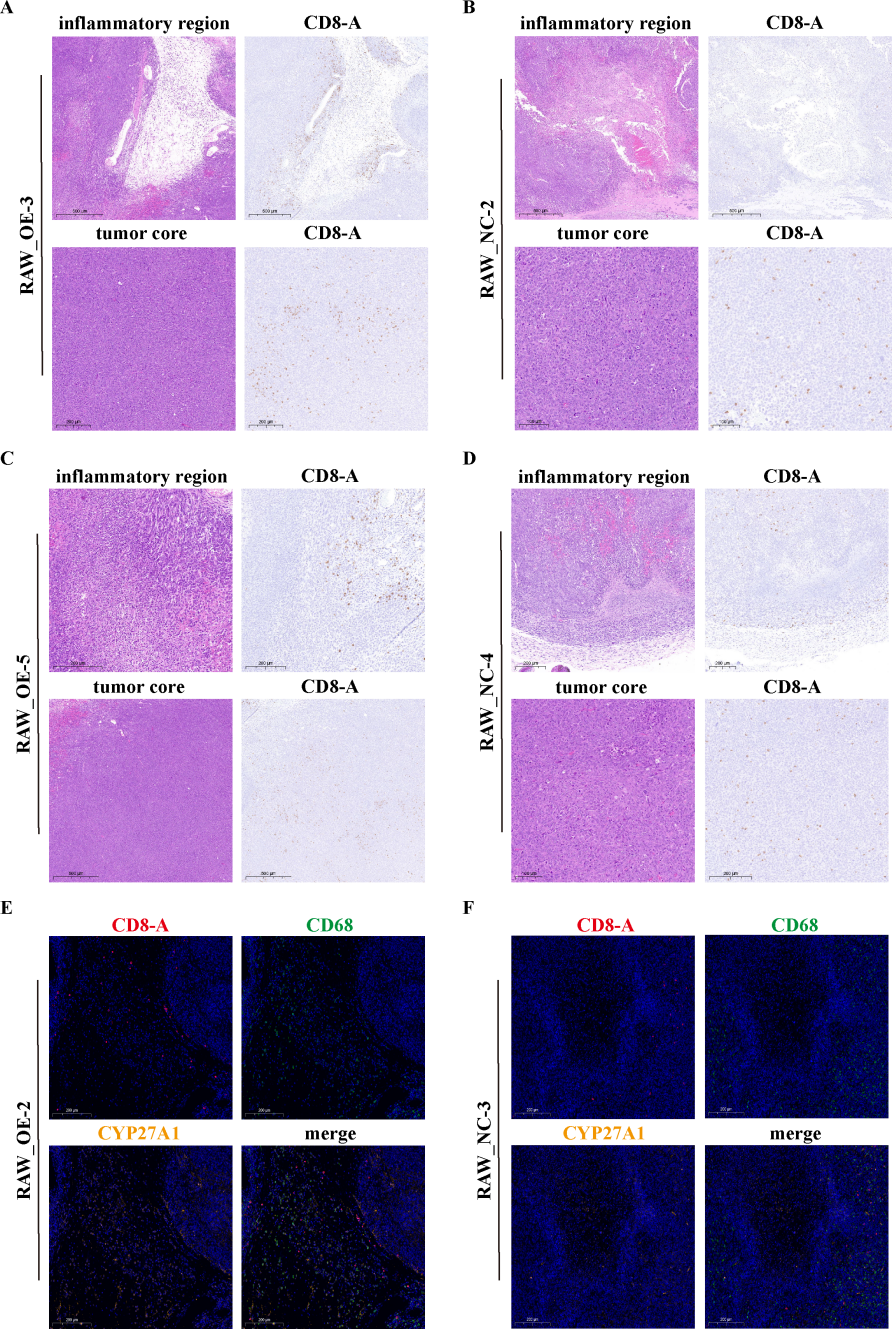
Morphological validation of CYP27A1^+^TAMs functions within the TIME **(A–D)**. H&E staining and immunohistochemistry (IHC) showing inflammatory regions and tumor parenchymal areas using anti-CD8 antibody. **(E, F)** Multiplex immunofluorescence of tumor sections showing CD8^+^T cells (red), CYP27A1 (orange), CD68 (green), and DAPI (blue).

To investigate whether this effect is mediated by CD8^+^T cells, we examined the differences from a morphological perspective. We performed serial sectioning of paraffin-embedded tumor tissues for subsequent Hematoxylin and Eosin (H&E) staining and immunohistochemistry (IHC) with an anti-CD8 antibody. In H&E-stained sections, potentially due to the nature of the subcutaneous tumor model, we did not observe significant differences in tertiary lymphoid structure formation. Furthermore, although the number of CD8^+^T cells in the tumor core of the RAW-OE group was somewhat higher than in the RAW-NC group, this difference was not statistically significant. The most notable distinction was observed in the inflammatory reaction zone at the tumor margin. This area was infiltrated by numerous neutrophils. In the RAW-OE group, a substantial number of CD8^+^T cells were clustered around the looser regions of this inflammatory zone. In contrast, very few T cells were visible inside or outside the inflammatory area in the RAW-NC group (**Figures 11A–D**).

To further explore whether CYP27A1^+^TAMs play a key role in recruiting CD8^+^T cells, we performed multiplex immunofluorescence (IF) on tumors from the RAW-OE and RAW-NC groups. The results revealed that the total number of CD68^+^macrophages was similar between the two groups. However, the RAW-OE group exhibited a significantly greater number of CYP27A1^+^, CD68^+^cells, alongside a markedly increased population of CD8^+^T cells. Spatial analysis confirmed the co-localization of these CYP27A1^+^TAMs and CD8^+^T cells (**Figures 11E, F**). This indicates that CYP27A1^+^TAMs play a crucial role in recruiting CD8^+^T cells within the TIME.

In conclusion, through our animal model, we have functionally and morphologically validated the discoveries from our single-cell and spatial transcriptomic analyses: macrophages with high CYP27A1 expression exert their anti-tumor function, at least in part, by recruiting more CD8^+^T cells to kill tumor cells.

## Discussion

The discovery of PD-L1 and the clinical application of immunotherapy have been a milestone in NSCLC treatment; however, to benefit more patients, a deeper understanding of the TIME remains imperative. Previous investigations into the TIME of NSCLC following immunotherapy have largely relied on dissecting individual molecular markers or cataloging cell types, providing essential yet fragmented insights that often overlook the higher-order spatial organization and multicellular interplay critical for therapeutic response (10). In contrast, this study introduces a systems-biology approach centered on spatial niches—locally organized cellular communities. By integrating single-cell and spatial transcriptomic data from multiple cohorts, we constructed a spatially resolved map of the TIME, bridging stromal and tumor compartments. This framework enabled a layered, multi-angle dissection of the post-ICB landscape. We systematically progressed from identifying key functional niches to pinpointing a pivotal cellular player, CYP27A1^+^TAMs, within the most anti-tumor niche. We further elucidated its spatial localization, functional role, and clinical relevance through a prognostic model, with validation from animal experiments and morphological analyses. This spatial niche-based paradigm shifts the focus from isolated components to the functional architecture of the TIME, offering a novel and more holistic conceptual lens to decipher therapy resistance and identify predictive biomarkers.

The complexity of TIME is three-dimensional: beyond the vastly variable gene expression, the spatial distribution of cells—especially their community tendencies—also warrants careful consideration. Although PD-L1 is highly detectable clinically, the response rate to therapy remains low (79), and the underlying biological truths are not yet fully understood (80). Spatial ecosystems have inspired new insights: Juhyun Oh et al. found that immunohistochemistry and the FAST platform’s CPS scoring have limited value in predicting responses of head and neck squamous cell carcinoma to anti-PD-1 therapy, but an ecosystem enriched with dendritic cells can effectively predict patient survival (81). Similarly, tertiary lymphoid structures (TLS) have been confirmed to hold prognostic value and are a hot topic in TIME research. As a specific spatial collection of immune cells, TLS itself is also a type of spatial ecosystem. In this study, we identified nine distinct ecosystems (CC1–CC9), which reveal both intra-sample heterogeneity and inter-sample similarities within NSCLC tumor microenvironments; understanding these similarities is crucial to deciphering TIME.

Among these, tumor-enriched ecosystems (CC2, CC4, CC6) display proliferative and immunosuppressive features. Particularly regarding tumor infiltration at the tumor margin, we explored cell interactions and spatial trajectories within these ecosystems, gaining a new perspective on tumor invasion behavior. Our data indicate that myCAFs and Tregs play key roles, involving neovascularization and immune suppression. We identified critical genes SDC1 and COMP involved in tumor infiltration; beyond their internal ecosystem interactions, their spatial distributions show high specificity. Previous studies have shown that POSTN plays an important role in forming fibrous immune barriers (8), and our data confirmed its high expression around tumors, validating this conclusion. Interestingly, COMP’s spatial expression pattern is even more specific, possibly making it a key gene in CAFs-mediated immune barrier formation. Combining spatial interaction data. Collectively, our spatial and interaction data nominate SDC1 and COMP as central players in facilitating tumor infiltration. To our knowledge, such combined analysis of spatial trajectories and ecosystems in NSCLC is novel. Notably, the key signals identified in infiltration show high overlap with differential proteins in cervical intraepithelial neoplasia(9/13) (82), suggesting that these tumor infiltration-related signals may also be pivotal in tumorigenesis.

In contrast, stroma-enriched immune ecosystems (CC1, CC3, CC9) activate inflammatory pathways such as NF-κB and TNFα. Notably, CC9 is significantly enriched in immunotherapy responders, which itself could serve as a predictive marker. TLS in our study was defined as CC8; these TLS are precisely captured within ecosystems. However, the number of TLS does not directly predict immunotherapy efficacy (9). We observed that both MPR-2 and NMPR-2 samples contain abundant TLS, but the stroma surrounding TLS differs (**Figure 2A**; **Supplementary Figure 2A**), which may be a key factor influencing treatment response. In analyzing CC9, we confirmed that it contains fewer immunosuppressive cells and more immune effector cells, which correlates with good prognosis. However, how to efficiently recruit and aggregate these immune cells remains a concern. Benefiting from the cell clustering tendencies of ecosystems, we identified CYP27A1^+^TAMs, which indeed plays a crucial role.

Macrophage classification in the tumor microenvironment has become increasingly detailed, especially regarding TAMs and lipid metabolism-related TAMs. We realized that CYP27A1^+^TAMs differs from M1 macrophages, functioning specifically as an anti-tumor cell mainly by recruiting T cells rather than exerting inflammatory cytotoxicity. It is similar to functionally specialized antigen-presenting macrophages (83), and highly expresses CYP27A1, which participates in cholesterol metabolism. Many macrophage subtypes studied previously—such as TREM2^+^TAMs, lipid-laden macrophages, and LPL^+^TAMs in our study—are strongly related to lipid metabolism and immune suppression (11, 12). Therefore, we cautiously sought validation through two single-cell datasets, two spatial transcriptomics datasets, and the Human Protein Atlas. Results confirmed that CYP27A1 is a lung macrophage-specific gene, consistently associated with pattern recognition receptor pathways. Additionally, our basic experimental results are consistent with these findings. The discovery of this functionally specialized macrophage is groundbreaking. Because this gene is broadly expressed in antigen-presenting macrophages within NSCLC, it can serve as a marker to identify these cells. In this study, CYP27A1^+^TAMs were extensively active in PR patients. Consequently, we developed a prognostic model based on it to assist clinical decision-making.

Many previous studies have shown that cholesterol metabolism influences NSCLC prognosis (84), and macrophages have a central role in TIME cholesterol metabolism (85). Our study identified that CYP27A1^+^TAMs, a specific macrophage subpopulation, are key mediators of this process, as they uniquely express the Liver X Receptor (LXR). Activation of LXR drives downstream molecules APOE, ABCA1 and ABCG1 to promote reverse cholesterol transport, leading to metabolic reprogramming within the TIME. Srustidhar Das et al. suggested that LXR activation can have anti-tumor effects by aiding immune cell recruitment and tertiary lymphoid structure formation (76). Sean B. Joseph et al. found that LXR deficiency affects cholesterol metabolism and increases bacterial susceptibility in mice (72), revealing a positive correlation between LXR and pattern recognition receptor pathways. Moreover, LXR activation upregulates SFTPA1 and SFTPA2, enhancing macrophage anti-apoptotic capacity (72), which may explain the high expression of LXR, SFTPA1, and SFTPA2 in macrophages after chemoradiotherapy in our study. Our data suggest that LXR activation is a pivotal process in the functional enhancement of CYP27A1^+^TAMs following therapy. Activation of LXR upregulates the expression of downstream target genes such as APOE, ABCG1, and ABCA1. These molecules, particularly APOE, are implicated in modulating the tumor immune microenvironment and may further enhance the ability of CYP27A1^+^TAMs to recruit CD8^+^T cells. However, the role of APOE in cancer immunity appears context-dependent. While some studies highlight APOE as a protective factor within the TIME (86), others associate it with poor responses to immunotherapy (87). A meta-analysis by Barakji et al. further suggests that the impact of APOE may vary with its different alleles in tumorigenesis and progression (88). These observations highlight the necessity for future research: subsequent work must build upon the current foundation to delve deeper into the specific impact of particular APOE genotypes on immunotherapy outcomes in NSCLC, investigate the functions of its different isoforms, and examine its role within specific spatial niches, ultimately to clarify its precise role in tumor immunity.

Regarding the translational potential of targeting the LXR pathway, the clinical accessibility is indeed promising. Endogenous oxysterols like 27-hydroxycholesterol can activate LXR and elevate APOE levels. However, systemic pharmacological LXR activation to promote cholesterol efflux from TAMs carries the potential risk of increasing circulating lipid levels, which may inadvertently elevate cardiovascular risks. Thus, while the pathway is tractable, its clinical translation requires careful consideration.

While our integrative analysis provides novel insights, several limitations should be noted. First, the spatial resolution of the VISIUM platform (55 µm spot diameter) limits the analysis of direct cell-cell contacts. Second, the sample size and heterogeneity of the discovery cohort may affect the generalizability of findings across NSCLC subtypes. Third, the precise molecular mechanisms by which LXR activation in CYP27A1^+^TAMs recruits T cells require further elucidation. Finally, the clinical translation of our findings, including validation of the CMRS prognostic model and exploration of LXR agonists as immunotherapeutic adjuvants, warrants future investigation.

In summary, this study delineates the diverse spatial ecosystems in NSCLC by integrating single−cell and spatial transcriptomics, revealing the cellular heterogeneity and dynamic interactions within tumor and stromal regions. It highlights the distinct roles of tumor−enriched and stroma−enriched immune ecosystems in tumor progression and immunotherapy response. Notably, we identified CYP27A1^+^TAMs as a novel lipid−metabolism−regulating and anti−tumor macrophage subset. Functionally, it is defined as a specialized antigen−presenting hub, characterized by a distinct metabolic profile centered on the LXR/CYP27A1 axis and a pro−survival phenotype. This combination of features clearly differentiates it from broader categories such as M1−like macrophages, TREM2^+^TAMs, lipid−laden macrophages, and LPL^+^TAMs. This subset promotes anti−tumor immunity by recruiting CD8^+^T cells, an effect that can be enhanced through activation of the LXR pathway. These findings position CYP27A1 and LXR as promising biomarkers and therapeutic targets, providing a rational framework for future spatially-informed, personalized combination therapies.

## Data Availability

The original contributions presented in the study are included in the article/**Supplementary Material**. Further inquiries can be directed to the corresponding author.

## Supplementary material

Supplementary Figure 1 Subpopulation clustering of single-cell samples**(A).** UMAP visualization after batch effect correction (by sequencing platform) using Harmony.**(B)** Correlation analysis between epithelial cells from patient P05 and the top 5% of cells with CNV variations; red cells classified as tumor epithelial, blue as normal epithelial.**(C)** Copy number variation (CNV) patterns across chromosomes in epithelial cells inferred by inferCNV, with endothelial cells as reference.**(D)** Dot plot displaying marker genes for T-cell subpopulations.**(E)** Dot plot displaying marker genes for myeloid cell subpopulations.

Supplementary Figure 2 Spatial Niche Composition of ST Samples**(A).** Spatial niche clustering (Composition Cluster, CC) visualization for additional samples. **(B)** Stacked bar plot showing the patient composition across different CC groups**(C)** Boxplot displaying the distribution of ssGSEA scores for plasma cells across CC groups. **(D)** Boxplot displaying the distribution of ssGSEA scores for B cells across CC groups.

Supplementary Figure 3 Cell type composition and cellular co-occurrence within tumor niches**(A).** Proportional composition of major cell types identified in the CC2, CC6, and CC4 niches. **(B)** Pairwise co-occurrence patterns among cell types within the CC4 and CC6 niches. Red indicates a positive correlation, blue indicates a negative correlation, and gray denotes no significant correlation. **(C)** Circle plot illustrating interaction strengths between different niches, highlighting key inter-niche communication networks. **(D)** Forest plot presenting the results of multivariate logistic regression in the IMvigor210 cohort.

Supplementary Figure 4 Correlation analysis of different cell types and TLS with CTLs activity in CC9.

Supplementary Figure 5 Cell communication and spatial localization reveal that CYP27A1^+^TAMs function through T cell recruitment **(A)**. Violin plot showing the expression of the CYP27A1 gene across all clusters. **(B)** Heatmap displaying the expression intensity of alveolar-associated genes in different macrophages. **(C, D)** Spatial interaction plot demonstrating CD86 signaling pathway interactions in S_MPR and S_NMPR samples. **(E–F)** Co-localization strength heatmap of Misty cell types in the juxta and para perspectives, with deeper colors indicating stronger co-localization signals. **(G–H)** Co-localization strength schematic for Misty cell types in the juxta and para perspectives, with a cutoff threshold set at 1.5.

Supplementary Figure 6 Visualization of Ripley’s K Function analysis for spatial transcriptomics samples.

Supplementary Figure 7 Correlation analysis of APOE, ABCG1, and ABCA1 with CD8A and CD8B expression in TCGA-LUAD.

Supplementary Figure 8 CMRS stability validation**(A).** Bar plot showing the distribution of C-indices from 1000 bootstrap resampling iterations of the CMRS. **(B)** Bar and box plot showing the distribution of C-indices for the CMRS across 500 different random seeds. **(C, D)** Calibration curves demonstrating the accuracy of survival prediction in different cohorts.

Supplementary Figure 9 Sample Processing for GSE131907 and E-MTAB-13530**(A).** Bar plots showing quality control metrics for GSE131907. **(B)** UMAP visualization after batch effect removal across sequencing samples using Harmony. **(C)** UMAP visualization of marker genes for major cell types in GSE131907. **(D)** Bubble plot illustrating the expression of CYP27A1 and LXR receptors across various cell types. **(E)** Stacked bar plot displaying the distribution of macrophages stratified by CYP27A1 expression level across different tissue types. **(F)** Stacked bar plot showing the distribution of macrophages stratified by CYP27A1 expression level within individual samples. **(G, H)** Spatial feature plots showing the co-localization of CD8A, CD8B, and CYP27A1 genes in an E-MTAB-13530 sample. Gene expression was normalized using SCTransform.

Supplementary Figure 10 Processing of GSE223203 and GSE267960 **(A).** UMAP visualization after batch effect removal across sequencing samples using Harmony. **(B)** Violin plots showing quality control metrics for the GSE223203 dataset. **(C)** Bubble plot displaying marker genes for major cell types in GSE223203. **(D)** Stacked bar plot illustrating the sample origin proportion of macrophages stratified by CYP27A1 expression level. **(E)** Spatial feature plots showing the co-localization of CD8A and CYP27A1 genes in an E-MTAB-13530 sample. Gene expression was normalized using SCTransform. **(F)** UMAP plot showing the expression profile of cluster 58 (associated with innate immune response) based on data from the Human Protein Atlas. **(G)** Radar chart displaying GSVA scores of different cell populations for Cluster 58. **(H)** Bar plot illustrating Jaccard similarity scores of different cell populations for Cluster 58.

## References

[1] Hendriks LEL Remon J Faivre-Finn C Garassino MC Heymach JV Kerr KM . Non-small-cell lung cancer. Nat Rev Dis Primer. (2024) 10:71. doi: 10.1038/s41572-024-00551-9, PMID: 39327441

[2] Lahiri A Maji A Potdar PD Singh N Parikh P Bisht B . Lung cancer immunotherapy: progress, pitfalls, and promises. Mol Cancer. (2023) 22:40. doi: 10.1186/s12943-023-01740-y, PMID: 36810079 PMC9942077

[3] Memon D Schoenfeld AJ Ye D Fromm G Rizvi H Zhang X . Clinical and molecular features of acquired resistance to immunotherapy in non-small cell lung cancer. Cancer Cell. (2024) 42:209–224.e9. doi: 10.1016/j.ccell.2023.12.013, PMID: 38215748 PMC11249385

[4] Caushi JX Zhang J Ji Z Vaghasia A Zhang B Hsiue EH-C . Transcriptional programs of neoantigen-specific TIL in anti-PD-1-treated lung cancers. Nature. (2021) 596:126–32. doi: 10.1038/s41586-021-03752-4, PMID: 34290408 PMC8338555

[5] Christodoulou M-I Zaravinos A . Single-cell analysis in immuno-oncology. Int J Mol Sci. (2023) 24:8422. doi: 10.3390/ijms24098422, PMID: 37176128 PMC10178969

[6] Chen H Deng C Gao J Wang J Fu F Wang Y . Integrative spatial analysis reveals tumor heterogeneity and immune colony niche related to clinical outcomes in small cell lung cancer. Cancer Cell. (2025) 43:519–536.e5. doi: 10.1016/j.ccell.2025.01.012, PMID: 39983726

[7] Chu X Tian Y Lv C . Decoding the spatiotemporal heterogeneity of tumor-associated macrophages. Mol Cancer. (2024) 23:150. doi: 10.1186/s12943-024-02064-1, PMID: 39068459 PMC11282869

[8] Chen C Guo Q Liu Y Hou Q Liao M Guo Y . Single-cell and spatial transcriptomics reveal *POSTN*^+^ cancer-associated fibroblasts correlated with immune suppression and tumour progression in non-small cell lung cancer. Clin Transl Med. (2023) 13:e1515. doi: 10.1002/ctm2.1515, PMID: 38115703 PMC10731139

[9] Yan Y Sun D Hu J Chen Y Sun L Yu H . Multi-omic profiling highlights factors associated with resistance to immuno-chemotherapy in non-small-cell lung cancer. Nat Genet. (2025) 57:126–39. doi: 10.1038/s41588-024-01998-y, PMID: 39658657

[10] Hume DA Millard SM Pettit AR . Macrophage heterogeneity in the single-cell era: facts and artifacts. Blood. (2023) 142:1339–47. doi: 10.1182/blood.2023020597, PMID: 37595274

[11] Tan J Fan W Liu T Zhu B Liu Y Wang S . TREM2+ macrophages suppress CD8+ T-cell infiltration after transarterial chemoembolisation in hepatocellular carcinoma. J Hepatol. (2023) 79:126–40. doi: 10.1016/j.jhep.2023.02.032, PMID: 36889359

[12] Kloosterman DJ Erbani J Boon M Farber M Handgraaf SM Ando-Kuri M . Macrophage-mediated myelin recycling fuels brain cancer Malignancy. Cell. (2024) 187:5336–5356.e30. doi: 10.1016/j.cell.2024.07.030, PMID: 39137777 PMC11429458

[13] De Zuani M Xue H Park JS Dentro SC Seferbekova Z Tessier J . Single-cell and spatial transcriptomics analysis of non-small cell lung cancer. Nat Commun. (2024) 15:4388. doi: 10.1038/s41467-024-48700-8, PMID: 38782901 PMC11116453

[14] Hao Y Hao S Andersen-Nissen E Mauck WM Zheng S Butler A . Integrated analysis of multimodal single-cell data. Cell. (2021) 184:3573–3587.e29. doi: 10.1016/j.cell.2021.04.048, PMID: 34062119 PMC8238499

[15] Cable DM Murray E Zou LS Goeva A Macosko EZ Chen F . Robust decomposition of cell type mixtures in spatial transcriptomics. Nat Biotechnol. (2022) 40:517–26. doi: 10.1038/s41587-021-00830-w, PMID: 33603203 PMC8606190

[16] Miller BF Huang F Atta L Sahoo A Fan J . Reference-free cell type deconvolution of multi-cellular pixel-resolution spatially resolved transcriptomics data. Nat Commun. (2022) 13:2339. doi: 10.1038/s41467-022-30033-z, PMID: 35487922 PMC9055051

[17] Lafzi A Borrelli C Baghai Sain S Bach K Kretz JA Handler K . Identifying spatial Co-occurrence in healthy and InflAmed tissues (ISCHIA). Mol Syst Biol. (2024) 20:98–119. doi: 10.1038/s44320-023-00006-5, PMID: 38225383 PMC10897385

[18] Schubert M Klinger B Klünemann M Sieber A Uhlitz F Sauer S . Perturbation-response genes reveal signaling footprints in cancer gene expression. Nat Commun. (2018) 9:20. doi: 10.1038/s41467-017-02391-6, PMID: 29295995 PMC5750219

[19] Borcherding N Vishwakarma A Voigt AP Bellizzi A Kaplan J Nepple K . Mapping the immune environment in clear cell renal carcinoma by single-cell genomics. Commun Biol. (2021) 4:122. doi: 10.1038/s42003-020-01625-6, PMID: 33504936 PMC7840906

[20] Kueckelhaus J Frerich S Kada-Benotmane J Koupourtidou C Ninkovic J Dichgans M . Inferring histology-associated gene expression gradients in spatial transcriptomic studies. Nat Commun. (2024) 15:7280. doi: 10.1038/s41467-024-50904-x, PMID: 39179527 PMC11343836

[21] Matchett KP Wilson-Kanamori JR Portman JR Kapourani CA Fercoq F May S . Multimodal decoding of human liver regeneration. Nature. (2024) 630:158–65. doi: 10.1038/s41586-024-07376-2, PMID: 38693268 PMC11153152

[22] Hänzelmann S Castelo R Guinney J . GSVA: gene set variation analysis for microarray and RNA-seq data. BMC Bioinf. (2013) 14:7. doi: 10.1186/1471-2105-14-7, PMID: 23323831 PMC3618321

[23] Jin S Guerrero-Juarez CF Zhang L Chang I Ramos R Kuan C-H . Inference and analysis of cell-cell communication using CellChat. Nat Commun. (2021) 12:1088. doi: 10.1038/s41467-021-21246-9, PMID: 33597522 PMC7889871

[24] Tanevski J Flores ROR Gabor A Schapiro D Saez-Rodriguez J . Explainable multiview framework for dissecting spatial relationships from highly multiplexed data. Genome Biol. (2022) 23:97. doi: 10.1186/s13059-022-02663-5, PMID: 35422018 PMC9011939

[25] Becker T Rousseau A-J Geubbelmans M Burzykowski T Valkenborg D . Decision trees and random forests. Am J Orthod Dentofacial Orthop. (2023) 164:894–7. doi: 10.1016/j.ajodo.2023.09.011, PMID: 38008491

[26] Binder H Allignol A Schumacher M Beyersmann J . Boosting for high-dimensional time-to-event data with competing risks. Bioinformatics. (2009) 25:890–6. doi: 10.1093/bioinformatics/btp088, PMID: 19244389

[27] Candia J Tsang JS . eNetXplorer: an R package for the quantitative exploration of elastic net families for generalized linear models. BMC Bioinf. (2019) 20:189–200. doi: 10.1186/s12859-019-2778-5, PMID: 30991955 PMC6469092

[28] Atkinson EJ Therneau TM Melton LJ Camp JJ Achenbach SJ Amin S . Assessing fracture risk using gradient boosting machine (GBM) models. J Bone Miner Res. (2012) 27:1397–404. doi: 10.1002/jbmr.1577, PMID: 22367889 PMC3408850

[29] Nestler S Humberg S . A lasso and a regression tree mixed-effect model with random effects for the level, the residual variance, and the autocorrelation. Psychometrika. (2022) 87:506–32. doi: 10.1007/s11336-021-09787-w, PMID: 34390456 PMC9166855

[30] Zhang S Ta N Zhang S Li S Zhu X Kong L . Unraveling pancreatic ductal adenocarcinoma immune prognostic signature through a naive B cell gene set. Cancer Lett. (2024) 594:216981. doi: 10.1016/j.canlet.2024.216981, PMID: 38795761

[31] Friedman J Hastie T Tibshirani R . Regularization paths for generalized linear models via coordinate descent. J Stat Software. (2010) 33:0–20. doi: 10.18637/jss.v033.i01 PMC2929880 20808728

[32] Shi M Zhang B . Semi-supervised learning improves gene expression-based prediction of cancer recurrence. Bioinformatics. (2011) 27:3017–23. doi: 10.1093/bioinformatics/btr502, PMID: 21893520 PMC3198572

[33] Van Belle V Pelckmans K Van Huffel S Suykens JAK . Improved performance on high-dimensional survival data by application of survival-SVM. Bioinformatics. (2011) 27:87–94. doi: 10.1093/bioinformatics/btq617, PMID: 21062763

[34] Sun X Liu W Sun L Mo H Feng Y Wu X . Maturation and abundance of tertiary lymphoid structures are associated with the efficacy of neoadjuvant chemoimmunotherapy in resectable non-small cell lung cancer. J Immunother Cancer. (2022) 10:e005531. doi: 10.1136/jitc-2022-005531, PMID: 37011953 PMC9644367

[35] Yang C You J Wang Y Chen S Tang Y Chen H . TLS and immune cell profiling: immunomodulatory effects of immunochemotherapy on tumor microenvironment in resectable stage III NSCLC. Front Immunol. (2024) 15. doi: 10.3389/fimmu.2024.1499731, PMID: 39726591 PMC11670196

[36] Chen Z Wang X Jin Z Li B Jiang D Wang Y . Deep learning on tertiary lymphoid structures in hematoxylin-eosin predicts cancer prognosis and immunotherapy response. NPJ Precis Oncol. (2024) 8:73. doi: 10.1038/s41698-024-00579-w, PMID: 38519580 PMC10959936

[37] Ullah R Yin Q Snell AH Wan L . RAF-MEK-ERK pathway in cancer evolution and treatment. Semin Cancer Biol. (2022) 85:123–54. doi: 10.1016/j.semcancer.2021.05.010, PMID: 33992782

[38] Napolitano S Martini G Ciardiello D Del Tufo S Martinelli E Troiani T . Targeting the EGFR signalling pathway in metastatic colorectal cancer. Lancet Gastroenterol Hepatol. (2024) 9:664–76. doi: 10.1016/S2468-1253(23)00479-X, PMID: 38697174

[39] Glaviano A Foo ASC Lam HY Yap KCH Jacot W Jones RH . PI3K/AKT/mTOR signaling transduction pathway and targeted therapies in cancer. Mol Cancer. (2023) 22:138. doi: 10.1186/s12943-023-01827-6, PMID: 37596643 PMC10436543

[40] Wu W Sarhadi M Song X Xue J Dai Y Gustafsson J-A . Liver X receptors and estrogen receptor β, two players in a rare subtype of NSCLC. Int J Biol Sci. (2023) 19:2848–59. doi: 10.7150/ijbs.85164, PMID: 37324952 PMC10266082

[41] Vokes NI Chambers E Nguyen T Coolidge A Lydon CA Le X . Concurrent TP53 mutations facilitate resistance evolution in EGFR-mutant lung adenocarcinoma. J Thorac Oncol Off Publ Int Assoc Study Lung Cancer. (2022) 17:779–92. doi: 10.1016/j.jtho.2022.02.011, PMID: 35331964 PMC10478031

[42] Favaro F Luciano-Mateo F Moreno-Caceres J Hernández-Madrigal M Both D Montironi C . TRAIL receptors promote constitutive and inducible IL-8 secretion in non-small cell lung carcinoma. Cell Death Dis. (2022) 13:1046. doi: 10.1038/s41419-022-05495-0, PMID: 36522309 PMC9755151

[43] Tanaka K Sugisaka J Shiraishi Y Watanabe Y Daga H Azuma K . Serum VEGF-a as a biomarker for the addition of bevacizumab to chemo-immunotherapy in metastatic NSCLC. Nat Commun. (2025) 16:2825. doi: 10.1038/s41467-025-58186-7, PMID: 40121197 PMC11929838

[44] Abdel-Fattah MM Mohamed WR Hassanein EHM Arab HA Arafa EA . Role of NF-κB/ICAM-1, JAK/STAT-3, and apoptosis signaling in the anticancer effect of tangeretin against urethane-induced lung cancer in BALB/c mice. Life Sci. (2023) 325:121749. doi: 10.1016/j.lfs.2023.121749, PMID: 37142089

[45] Zhang L Ludden CM Cullen AJ Tew KD Branco de Barros AL Townsend DM . Nuclear factor kappa B expression in non-small cell lung cancer. BioMed Pharmacother. (2023) 167:115459. doi: 10.1016/j.biopha.2023.115459, PMID: 37716117 PMC10591792

[46] Lin A Zhang H Meng H Deng Z Gu T Luo P . TNF-alpha pathway alternation predicts survival of immune checkpoint inhibitors in non-small cell lung cancer. Front Immunol. (2021) 12. doi: 10.3389/fimmu.2021.667875, PMID: 34603277 PMC8481577

[47] Khaliq AM Rajamohan M Saeed O Mansouri K Adil A Zhang C . Spatial transcriptomic analysis of primary and metastatic pancreatic cancers highlights tumor microenvironmental heterogeneity. Nat Genet. (2024) 56:2455–65. doi: 10.1038/s41588-024-01914-4, PMID: 39294496

[48] Wang H Liang Y Liu Z Zhang R Chao J Wang M . POSTN^+^ cancer-associated fibroblasts determine the efficacy of immunotherapy in hepatocellular carcinoma. J Immunother Cancer. (2024) 12:e008721. doi: 10.1136/jitc-2023-008721, PMID: 39067872 PMC11284881

[49] Gao H Shen K Chen L Huang C Ji J Zhu X . SPP1 + macrophage and treg interaction mediates immunosuppression and adverse survival in HER2 + breast cancer. Sci Rep. (2025) 15:45403. doi: 10.1038/s41598-025-29530-0, PMID: 41298703 PMC12748558

[50] Blom AM Gialeli C Hagerling C Berntsson J Jirström K Papadakos KS . Expression of cartilage oligomeric matrix protein in colorectal cancer is an adverse prognostic factor and correlates negatively with infiltrating immune cells and PD-L1 expression. Front Immunol. (2023) 14. doi: 10.3389/fimmu.2023.1167659, PMID: 37207219 PMC10188999

[51] Sugiyanto RN Metzger C Inal A Truckenmueller F Gür K Eiteneuer E . Proteomic profiling reveals CEACAM6 function in driving gallbladder cancer aggressiveness through integrin receptor, PRKCD and AKT/ERK signaling. Cell Death Dis. (2024) 15:1–15. doi: 10.1038/s41419-024-07171-x, PMID: 39468006 PMC11519453

[52] Tai Y Chow A Han S Coker C Ma W Gu Y . FLT1 activation in cancer cells promotes PARP-inhibitor resistance in breast cancer. EMBO Mol Med. (2024) 16:1957–80. doi: 10.1038/s44321-024-00094-2, PMID: 38956205 PMC11319505

[53] Kadomatsu T Hara C Kurahashi R Horiguchi H Morinaga J Miyata K . ANGPTL2-mediated epigenetic repression of MHC-I in tumor cells accelerates tumor immune evasion. Mol Oncol. (2023) 17:2637–58. doi: 10.1002/1878-0261.13490, PMID: 37452654 PMC10701769

[54] Chen K Wang Q Li M Guo H Liu W Wang F . Single-cell RNA-seq reveals dynamic change in tumor microenvironment during pancreatic ductal adenocarcinoma Malignant progression. Ebiomedicine. (2021) 66:103315. doi: 10.1016/j.ebiom.2021.103315, PMID: 33819739 PMC8047497

[55] Zeltz C Kusche-Gullberg M Heljasvaara R Gullberg D . Novel roles for cooperating collagen receptor families in fibrotic niches. Curr Opin Cell Biol. (2023) 85:102273. doi: 10.1016/j.ceb.2023.102273, PMID: 37918273

[56] Wang T Jin H Hu J Li X Ruan H Xu H . COL4A1 promotes the growth and metastasis of hepatocellular carcinoma cells by activating FAK-src signaling. J Exp Clin Cancer Res. (2020) 39:148. doi: 10.1186/s13046-020-01650-7, PMID: 32746865 PMC7398077

[57] Guo J Ma X Liu D Wang F Xia J Zhang B . A distinct subset of urothelial cells with enhanced EMT features promotes chemotherapy resistance and cancer recurrence by increasing COL4A1-ITGB1 mediated angiogenesis. Drug Resist Updat. (2024) 76:101116. doi: 10.1016/j.drup.2024.101116, PMID: 38968684

[58] HM M Se S Ma M Syahir A . CD44: a multifunctional mediator of cancer progression. Biomolecules. (2021) 11:1850. doi: 10.3390/biom11121850, PMID: 34944493 PMC8699317

[59] Ding D Wang L Zhang Y Shi K Shen Y . Machine learning developed a programmed cell death signature for predicting prognosis and immunotherapy benefits in lung adenocarcinoma. Transl Oncol. (2023) 38:101784. doi: 10.1016/j.tranon.2023.101784, PMID: 37722290 PMC10511492

[60] Yang Z Chen S Ying H Yao W . Targeting syndecan-1: new opportunities in cancer therapy. Am J Physiol Cell Physiol. (2022) 323:C29–45. doi: 10.1152/ajpcell.00024.2022, PMID: 35584326 PMC9236862

[61] Guo B Wen X Yu S Yang J . Single-cell sequencing reveals PHLDA1-positive smooth muscle cells promote local invasion in head and neck squamous cell carcinoma. Transl Oncol. (2025) 55:102301. doi: 10.1016/j.tranon.2025.102301, PMID: 40132389 PMC11985064

[62] Wei J Yu W Wu L Chen Z Huang G Hu M . Intercellular molecular crosstalk networks within invasive and immunosuppressive tumor microenvironment subtypes associated with clinical outcomes in four cancer types. Biomedicines. (2023) 11:3057. doi: 10.3390/biomedicines11113057, PMID: 38002057 PMC10669098

[63] Nahmgoong H Jeon YG Park ES Choi YH Han SM Park J . Distinct properties of adipose stem cell subpopulations determine fat depot-specific characteristics. Cell Metab. (2022) 34:458–472.e6. doi: 10.1016/j.cmet.2021.11.014, PMID: 35021043

[64] Lai H Cheng X Liu Q Luo W Liu M Zhang M . Single-cell RNA sequencing reveals the epithelial cell heterogeneity and invasive subpopulation in human bladder cancer. Int J Cancer. (2021) 149:2099–115. doi: 10.1002/ijc.33794, PMID: 34480339

[65] Chen Z Chen W Lin K Chen X Lin G Li Y . Cancer-associated fibroblasts promote the proliferation and metastasis of colon cancer by mediating the RLIM/PML axis through paracrine COMP. J Gastroenterol Hepatol. (2024) 39:2677–89. doi: 10.1111/jgh.16713, PMID: 39162054

[66] Li K Huang Z Xie G Huang B Song L Zhang Y . Transcriptomic insights into UTUC: role of inflammatory fibrosis and potential for personalized treatment. J Transl Med. (2024) 22:24. doi: 10.1186/s12967-023-04815-y, PMID: 38183115 PMC10768331

[67] Di Modugno F Di Carlo A Spada S Palermo B D’Ambrosio L D’Andrea D . Tumoral and stromal hMENA isoforms impact tertiary lymphoid structure localization in lung cancer and predict immune checkpoint blockade response in patients with cancer. Ebiomedicine. (2024) 101:105003. doi: 10.1016/j.ebiom.2024.105003, PMID: 38340557 PMC10869748

[68] Qi J Sun H Zhang Y Wang Z Xun Z Li Z . Single-cell and spatial analysis reveal interaction of FAP+ fibroblasts and SPP1+ macrophages in colorectal cancer. Nat Commun. (2022) 13:1742. doi: 10.1038/s41467-022-29366-6, PMID: 35365629 PMC8976074

[69] Wang J Zhu N Su X Gao Y Yang R . Novel tumor-associated macrophage populations and subpopulations by single cell RNA sequencing. Front Immunol. (2023) 14:1264774. doi: 10.3389/fimmu.2023.1264774, PMID: 38347955 PMC10859433

[70] Marcovecchio PM Thomas G Salek-Ardakani S . CXCL9-expressing tumor-associated macrophages: new players in the fight against cancer. J Immunother Cancer. (2021) 9:e002045. doi: 10.1136/jitc-2020-002045, PMID: 33637602 PMC7919587

[71] Ramalingam PS Elangovan S Mekala JR Arumugam S . Liver X receptors (LXRs) in cancer-an eagle’s view on molecular insights and therapeutic opportunities. Front Cell Dev Biol. (2024) 12. doi: 10.3389/fcell.2024.1386102, PMID: 38550382 PMC10972936

[72] Joseph SB Bradley MN Castrillo A Bruhn KW Mak PA Pei L . LXR-dependent gene expression is important for macrophage survival and the innate immune response. Cell. (2004) 119:299–309. doi: 10.1016/j.cell.2004.09.032, PMID: 15479645

[73] Peng Y Wang Y Zhou C Mei W Zeng C . PI3K/akt/mTOR pathway and its role in cancer therapeutics: are we making headway? Front Oncol. (2022) 12:819128. doi: 10.3389/fonc.2022.819128, PMID: 35402264 PMC8987494

[74] Sun X Ou Z Chen R Niu X Chen D Kang R . Activation of the p62-Keap1-NRF2 pathway protects against ferroptosis in hepatocellular carcinoma cells. Hepatol Baltim Md. (2016) 63:173–84. doi: 10.1002/hep.28251, PMID: 26403645 PMC4688087

[75] Glaría E Letelier NA Valledor AF . Integrating the roles of liver X receptors in inflammation and infection: mechanisms and outcomes. Curr Opin Pharmacol. (2020) 53:55–65. doi: 10.1016/j.coph.2020.05.001, PMID: 32599447

[76] Das S Parigi SM Luo X Fransson J Kern BC Okhovat A . Liver X receptor unlinks intestinal regeneration and tumorigenesis. Nature. (2025) 637:1198–206. doi: 10.1038/s41586-024-08247-6, PMID: 39567700 PMC11779645

[77] Litvinchuk A Suh JH Guo JL Lin K Davis SS Bien-Ly N . Amelioration of tau and ApoE4-linked glial lipid accumulation and neurodegeneration with an LXR agonist. Neuron. (2024) 112:384–403.e8. doi: 10.1016/j.neuron.2023.10.023, PMID: 37995685 PMC10922706

[78] Wang X Shi J Huang M Chen J Dan J Tang Y . TUBB2B facilitates progression of hepatocellular carcinoma by regulating cholesterol metabolism through targeting HNF4A/CYP27A1. Cell Death Dis. (2023) 14:179. doi: 10.1038/s41419-023-05687-2, PMID: 36872411 PMC9986231

[79] Murata D Azuma K Murotani K Kawahara A Nishii Y Tokito T . Characterization of pre- and on-treatment soluble immune mediators and the tumor microenvironment in NSCLC patients receiving PD-1/L1 inhibitor monotherapy. Cancer Immunol Immunother CII. (2024) 73:214. doi: 10.1007/s00262-024-03781-8, PMID: 39235457 PMC11377373

[80] Genova C Dellepiane C Carrega P Sommariva S Ferlazzo G Pronzato P . Therapeutic implications of tumor microenvironment in lung cancer: focus on immune checkpoint blockade. Front Immunol. (2021) 12:799455. doi: 10.3389/fimmu.2021.799455, PMID: 35069581 PMC8777268

[81] Oh J Hoelzl J Carlson JCT Bill R Peterson HM Faquin WC . Spatial analysis identifies DC niches as predictors of pembrolizumab therapy in head and neck squamous cell cancer. Cell Rep Med. (2025) 6:102100. doi: 10.1016/j.xcrm.2025.102100, PMID: 40311615 PMC12147904

[82] Bober P Tkáčiková S Talian I Urdzík P Toporcerová S Sabo J . Differential urinary proteomic analysis of high-risk cervical intraepithelial neoplasia. Int J Mol Sci. (2023) 24:2531. doi: 10.3390/ijms24032531, PMID: 36768853 PMC9916937

[83] Appios A Davies J Sirvent S Henderson S Trzebanski S Schroth J . Convergent evolution of monocyte differentiation in adult skin instructs langerhans cell identity. Sci Immunol. (2024) 9:eadp0344. doi: 10.1126/sciimmunol.adp0344, PMID: 39241057 PMC7616733

[84] Feng S Zhang L Sun T Xu L Quan X Zhao G . OVOL1 promotes proliferation and metastasis of non-small cell lung cancer by regulating APOE-mediated cholesterol metabolism. J Cell Mol Med. (2025) 29:e70634. doi: 10.1111/jcmm.70634, PMID: 40437660 PMC12119240

[85] Vassiliou E Farias-Pereira R . Impact of lipid metabolism on macrophage polarization: implications for inflammation and tumor immunity. Int J Mol Sci. (2023) 24:12032. doi: 10.3390/ijms241512032, PMID: 37569407 PMC10418847

[86] Liu X Chang Y Long M Huang K Wang B Gong D . ApoE inhibits the progression of glioma by activating immune function. J Cell Mol Med. (2025) 29:e70697. doi: 10.1111/jcmm.70697, PMID: 40662483 PMC12261038

[87] Fan G Xie T Tang L Li L Han X Shi Y . The co-location of CD14+APOE+ cells and MMP7+ tumour cells contributed to worse immunotherapy response in non-small cell lung cancer. Clin Transl Med. (2024) 14:e70009. doi: 10.1002/ctm2.70009, PMID: 39187937 PMC11347392

[88] Barakji YA Hupfeld NB Bertelsen CA Frikke-Schmidt R Nielsen SF Rasmussen KL . Common genetic variation in the APOE gene and risk of cancer: a systematic review and meta-analysis. Atherosclerosis. (2025) 411:120568. doi: 10.1016/j.atherosclerosis.2025.120568, PMID: 41197273

