# Correction: Integrative multi-omics and machine learning reveals the spatial niche distribution and role of CYP27A1+TAMs in immunotherapy response in non-small cell lung cancer

**DOI:** 10.3389/fimmu.2026.1822612

**Published:** 2026-03-16

**Authors:** Qingsheng Liu, Xufeng Liu, Han Zhang, Yuhang Jiang, Ying Shi, Qiuqiao Mu, Yuhao Jing, Daqiang Sun

**Affiliations:** 1 Clinical School of Thoracic, Tianjin Medical University, Tianjin, China; 2 Tianjin Binhai New Area Haibin People’s Hospital, Tianjin, China; 3 Tianjin Chest Hospital, Tianjin University, Tianjin, China

**Keywords:** CD8+T cells, CYP27A1+TAMs, non-small cell lung cancer, spatial niche, tumor-associated macrophages

Affiliation “Tianjin Binhai New Area Haibin People’s Hospital, Tianjin, China” was omitted for author “Xufeng Liu”. This affiliation has now been added for author “Xufeng Liu”.

Author “Xufeng Liu” was erroneously assigned to affiliation “Department of Thoracic Surgery, Tianjin Chest Hospital, Tianjin, China”. This affiliation has now been removed for author “Xufeng Liu”.

The original version of this article has been updated.

