# Supplementary figures and images for: Integrative multi-omics and machine learning reveals the spatial niche distribution and role of CYP27A1+TAMs in immunotherapy response in non-small cell lung cancer
Source: Front Immunol. 2026 Feb 26;17:1782545. doi: 10.3389/fimmu.2026.1782545 (PMC12979556; doi:10.3389/fimmu.2026.1782545)

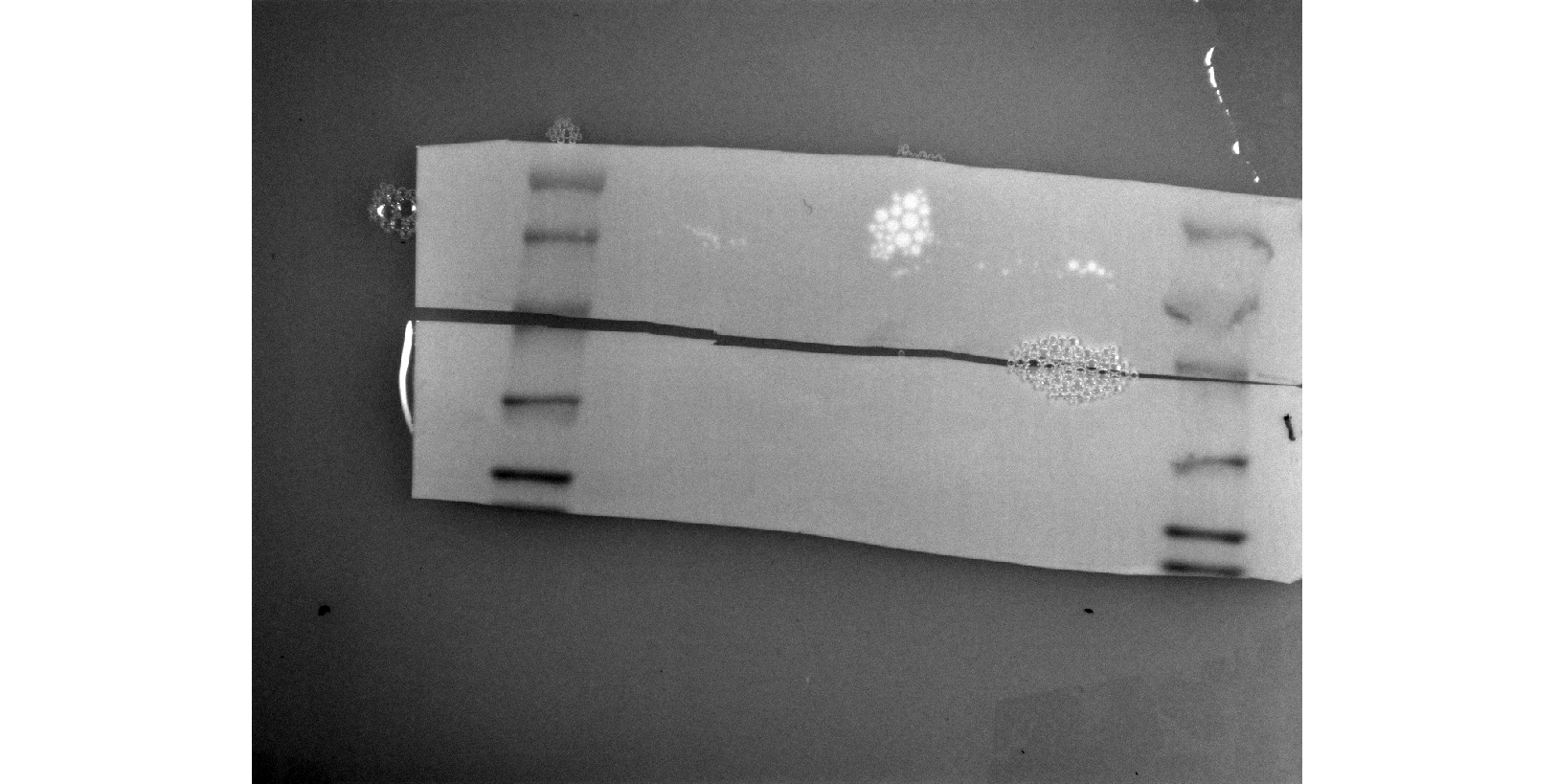

Supplement: Supplementary Figure 1 — Subpopulation clustering of single-cell samples(A). UMAP visualization after batch effect correction (by sequencing platform) using Harmony.(B) Correlation analysis between epithelial cells from patient P05 and the top 5% of cells with CNV variations; red cells classified as tumor epithelial, blue as normal epithelial.(C) Copy number variation (CNV) patterns across chromosomes in epithelial cells inferred by inferCNV, with endothelial cells as reference.(D) Dot plot displaying marker genes for T-cell subpopulations.(E) Dot plot displaying marker genes for myeloid cell subpopulations. [file Image1.tif]

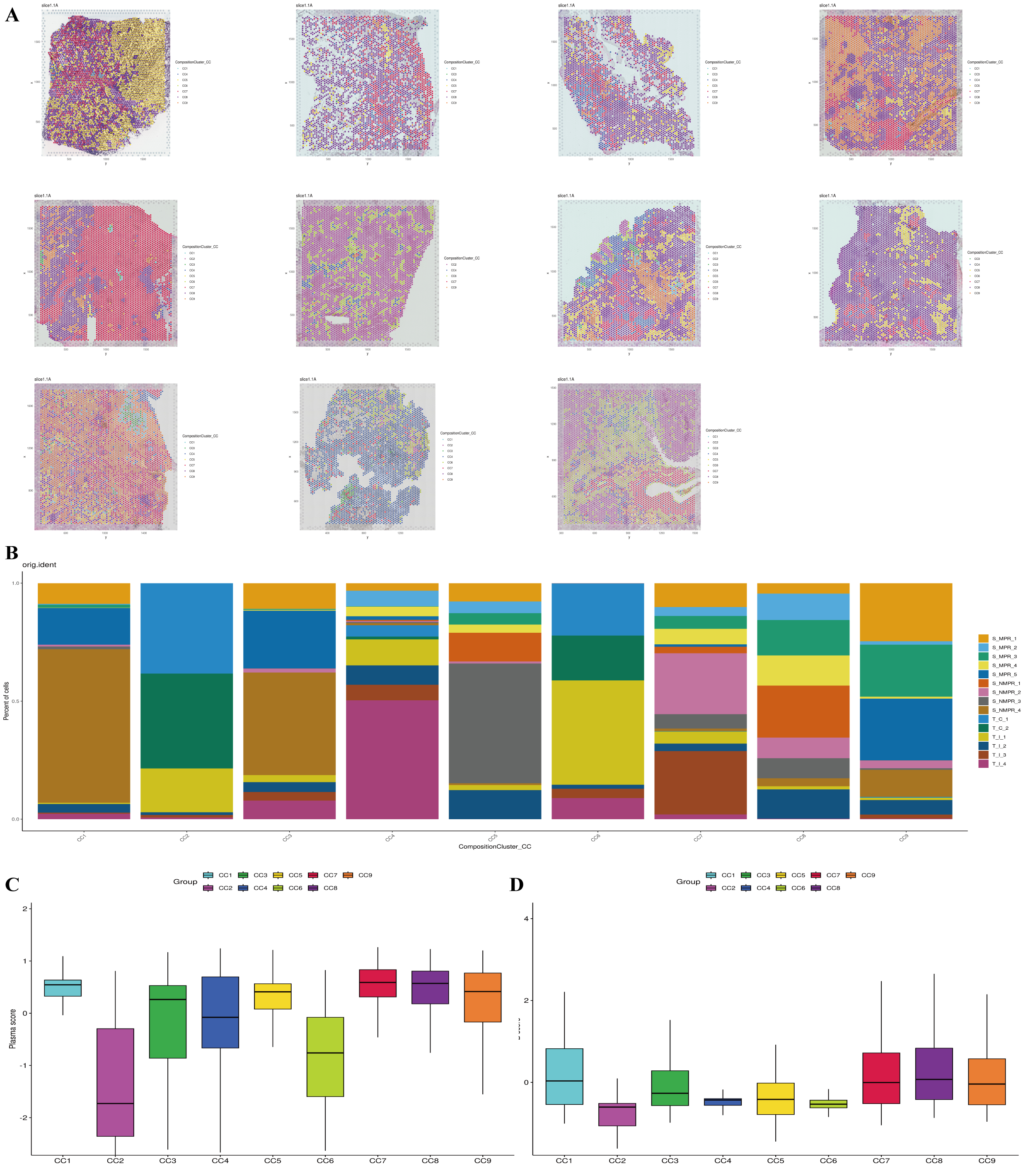

Supplement: Supplementary Figure 2 — Spatial Niche Composition of ST Samples(A). Spatial niche clustering (Composition Cluster, CC) visualization for additional samples. (B) Stacked bar plot showing the patient composition across different CC groups(C) Boxplot displaying the distribution of ssGSEA scores for plasma cells across CC groups. (D) Boxplot displaying the distribution of ssGSEA scores for B cells across CC groups. [file Image2.png]

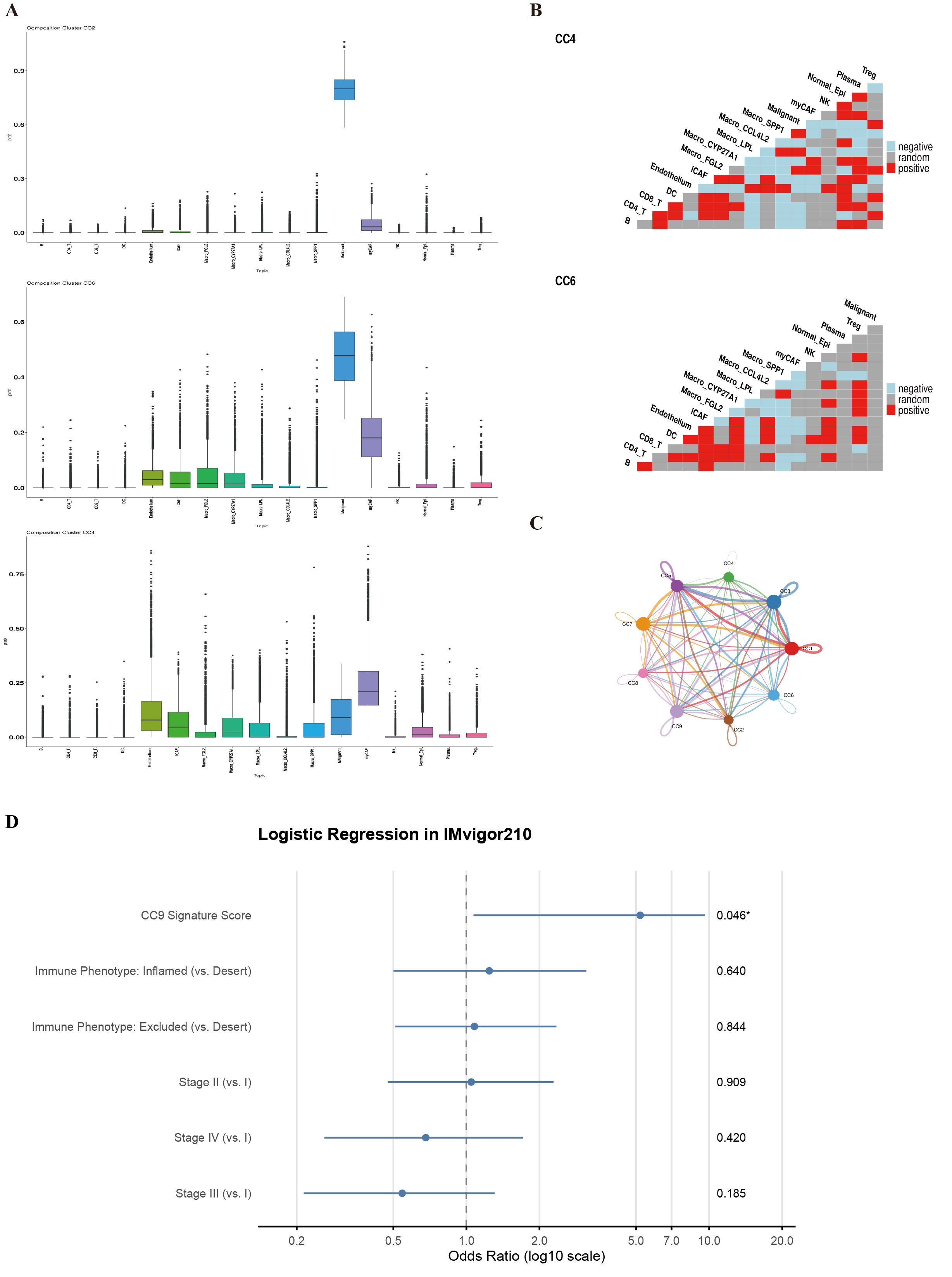

Supplement: Supplementary Figure 3 — Cell type composition and cellular co-occurrence within tumor niches(A). Proportional composition of major cell types identified in the CC2, CC6, and CC4 niches. (B) Pairwise co-occurrence patterns among cell types within the CC4 and CC6 niches. Red indicates a positive correlation, blue indicates a negative correlation, and gray denotes no significant correlation. (C) Circle plot illustrating interaction strengths between different niches, highlighting key inter-niche communication networks. (D) Forest plot presenting the results of multivariate logistic regression in the IMvigor210 cohort. [file Image3.png]

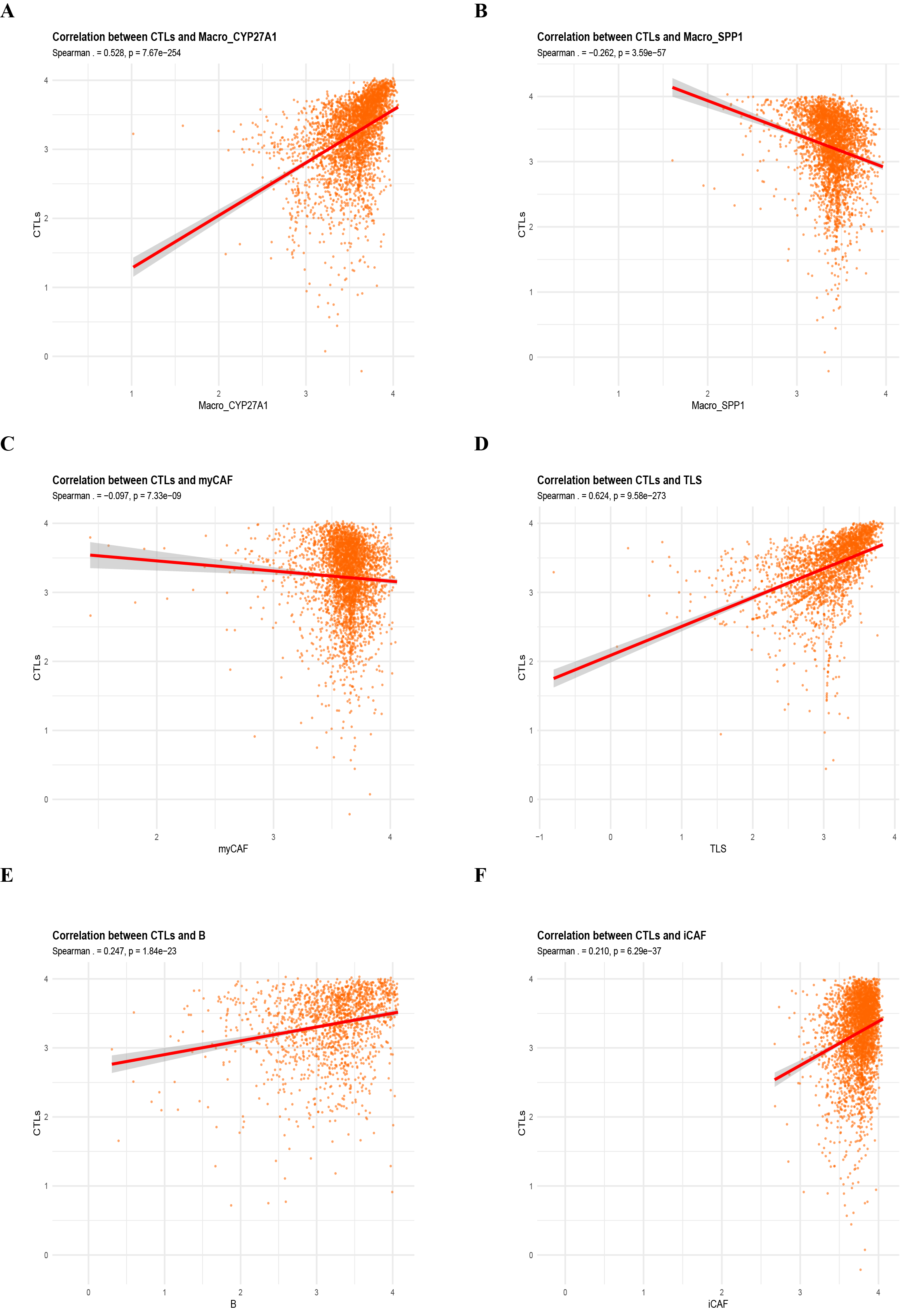

Supplement: Supplementary Figure 4 — Correlation analysis of different cell types and TLS with CTLs activity in CC9. [file Image4.png]

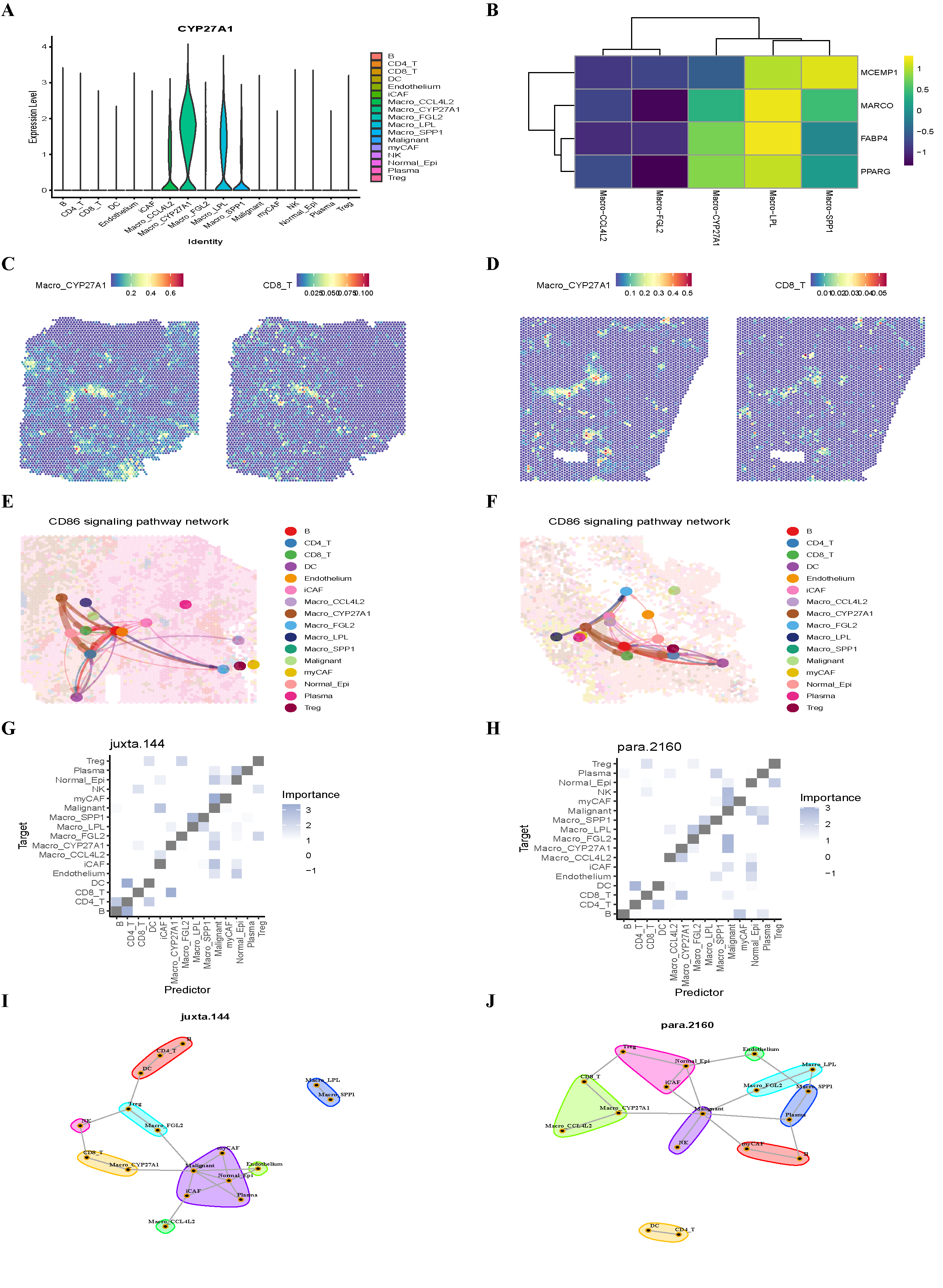

Supplement: Supplementary Figure 5 — Cell communication and spatial localization reveal that CYP27A1+TAMs function through T cell recruitment (A). Violin plot showing the expression of the CYP27A1 gene across all clusters. (B) Heatmap displaying the expression intensity of alveolar-associated genes in different macrophages. (C, D) Spatial interaction plot demonstrating CD86 signaling pathway interactions in S_MPR and S_NMPR samples. (E–F) Co-localization strength heatmap of Misty cell types in the juxta and para perspectives, with deeper colors indicating stronger co-localization signals. (G–H) Co-localization strength schematic for Misty cell types in the juxta and para perspectives, with a cutoff threshold set at 1.5. [file Image5.tif]

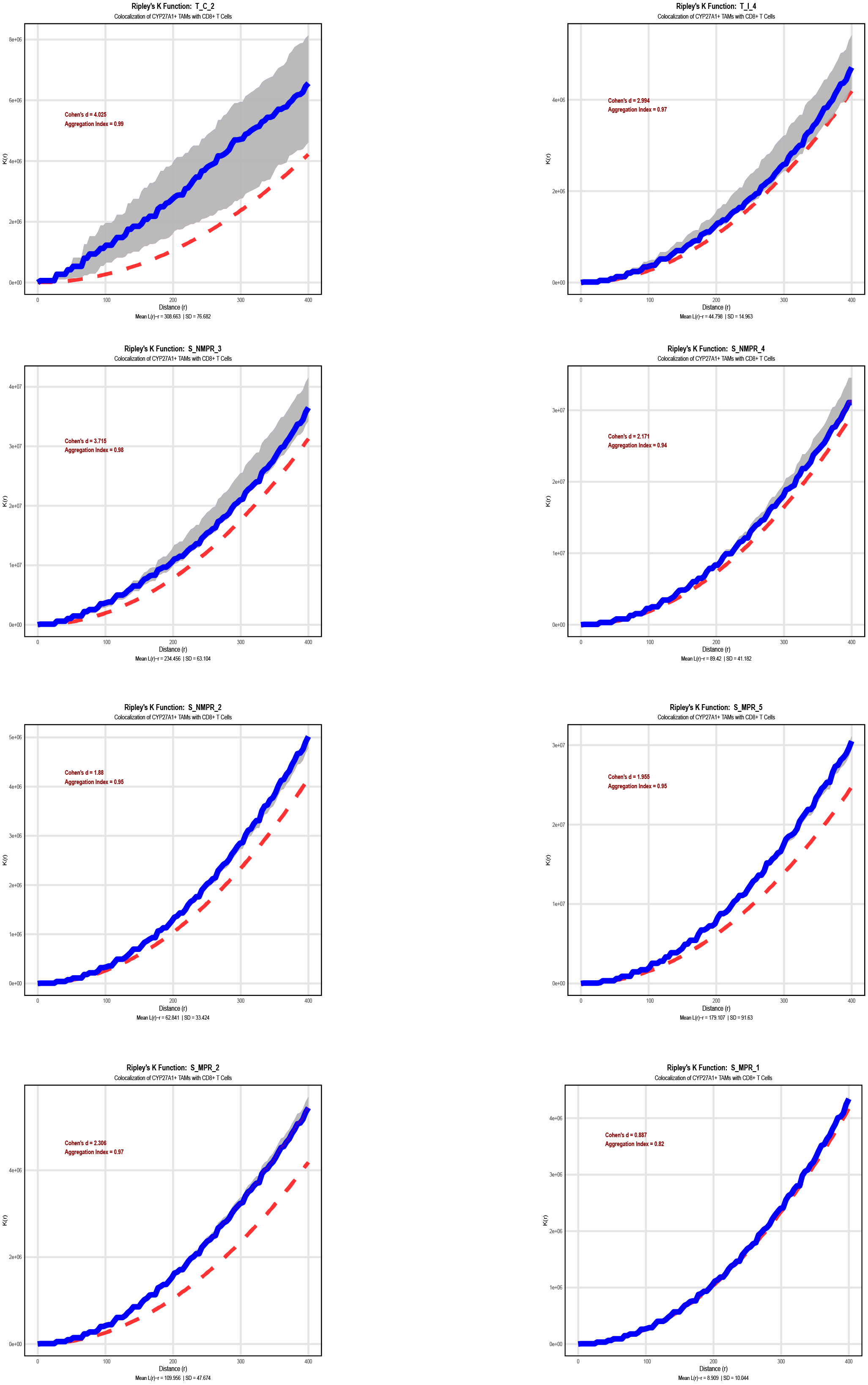

Supplement: Supplementary Figure 6 — Visualization of Ripley’s K Function analysis for spatial transcriptomics samples. [file Image6.png]

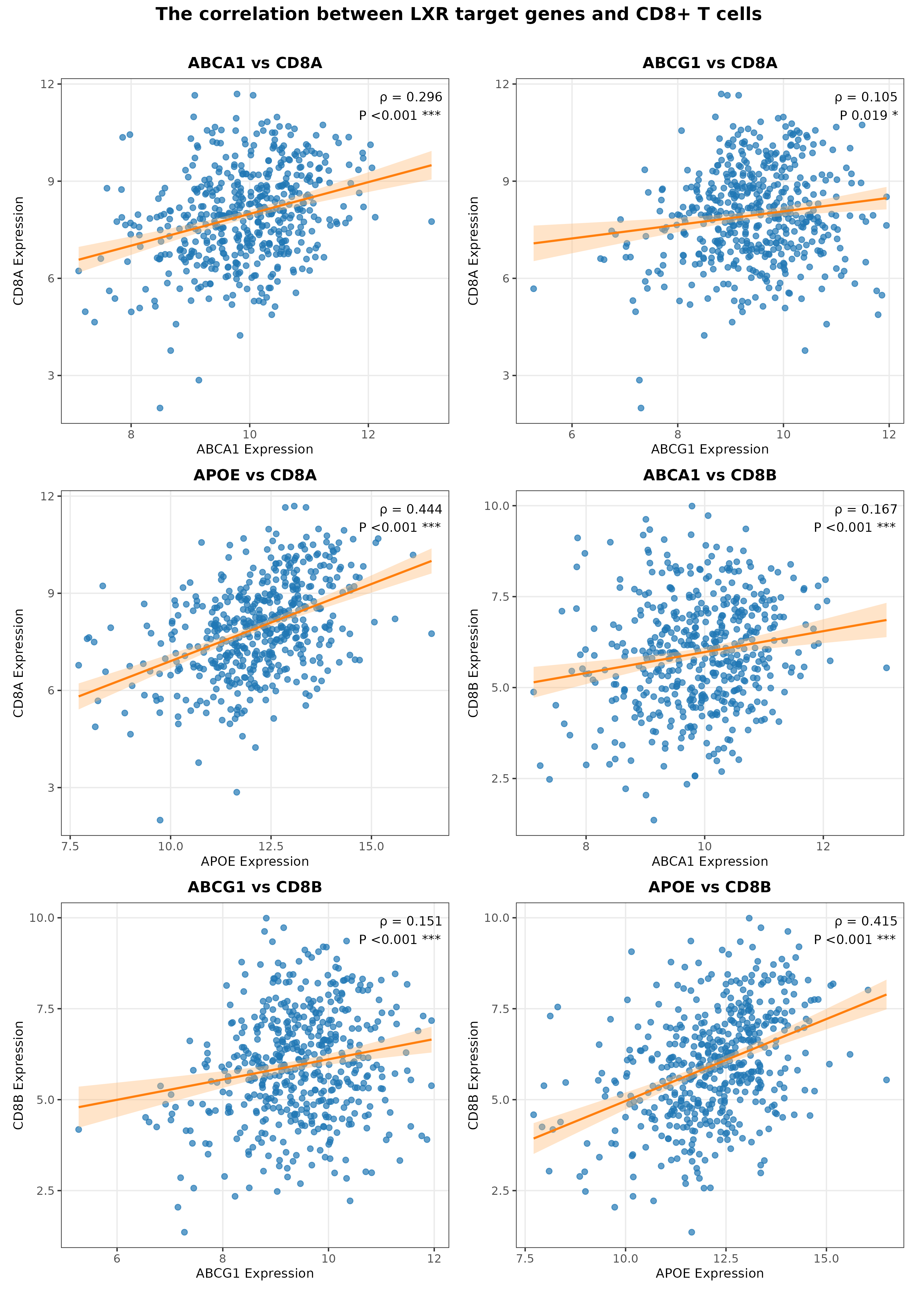

Supplement: Supplementary Figure 7 — Correlation analysis of APOE, ABCG1, and ABCA1 with CD8A and CD8B expression in TCGA-LUAD. [file Image7.png]

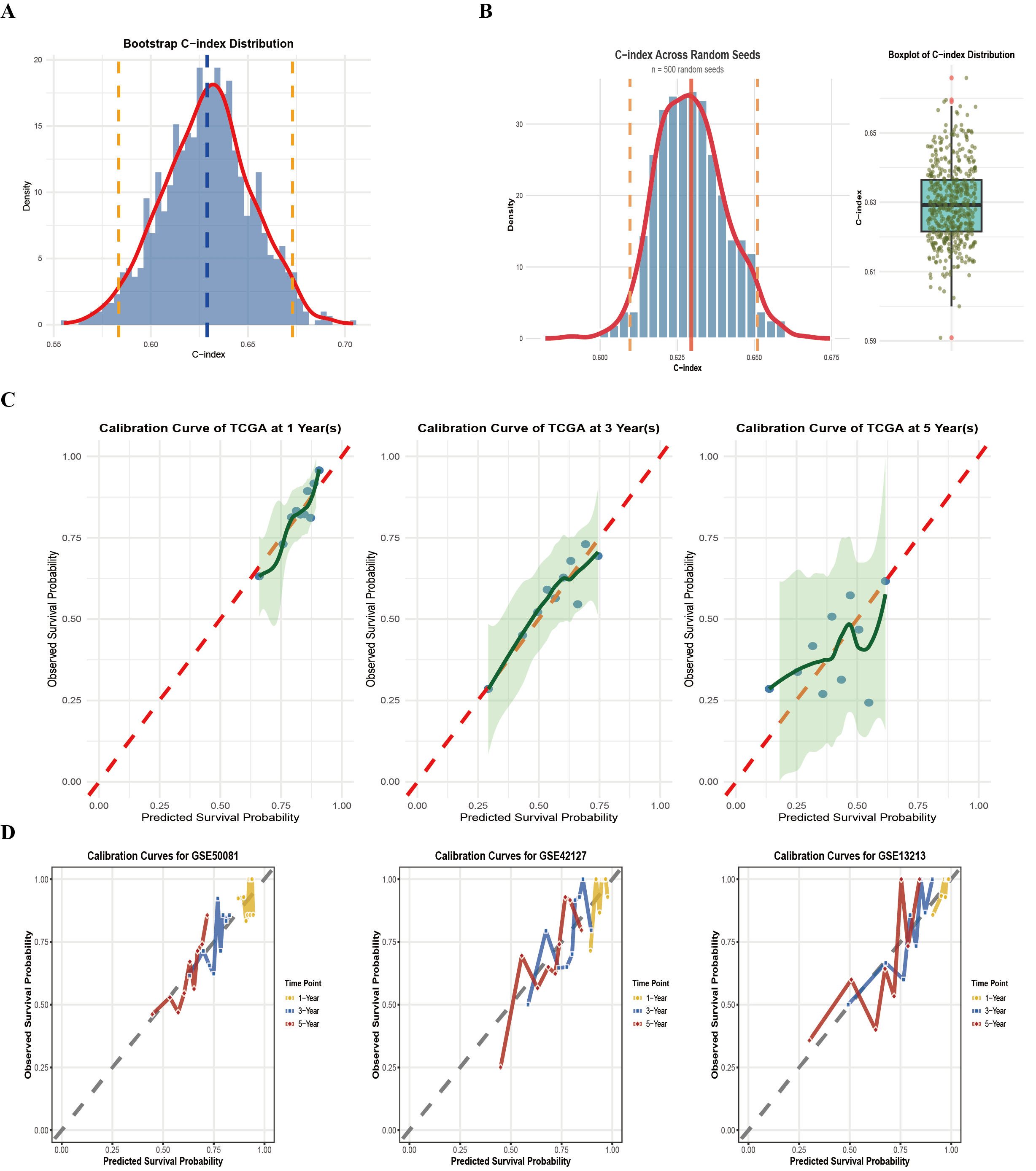

Supplement: Supplementary Figure 8 — CMRS stability validation(A). Bar plot showing the distribution of C-indices from 1000 bootstrap resampling iterations of the CMRS. (B) Bar and box plot showing the distribution of C-indices for the CMRS across 500 different random seeds. (C, D) Calibration curves demonstrating the accuracy of survival prediction in different cohorts. [file Image8.png]

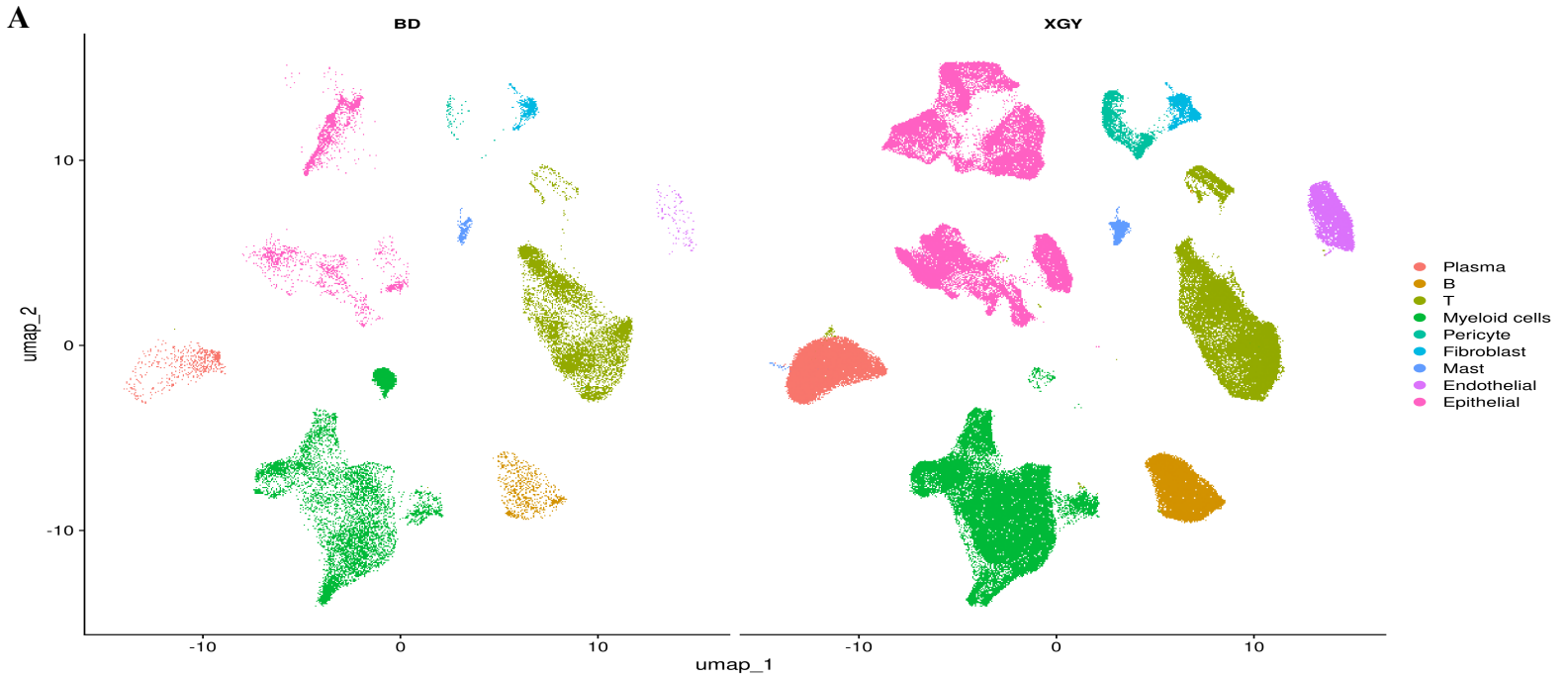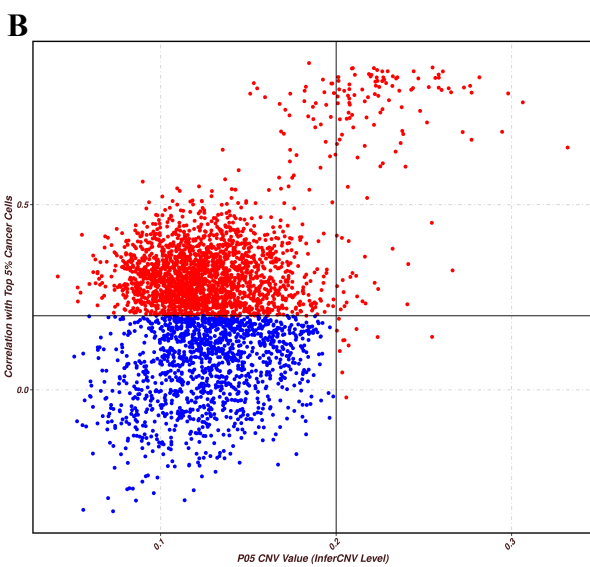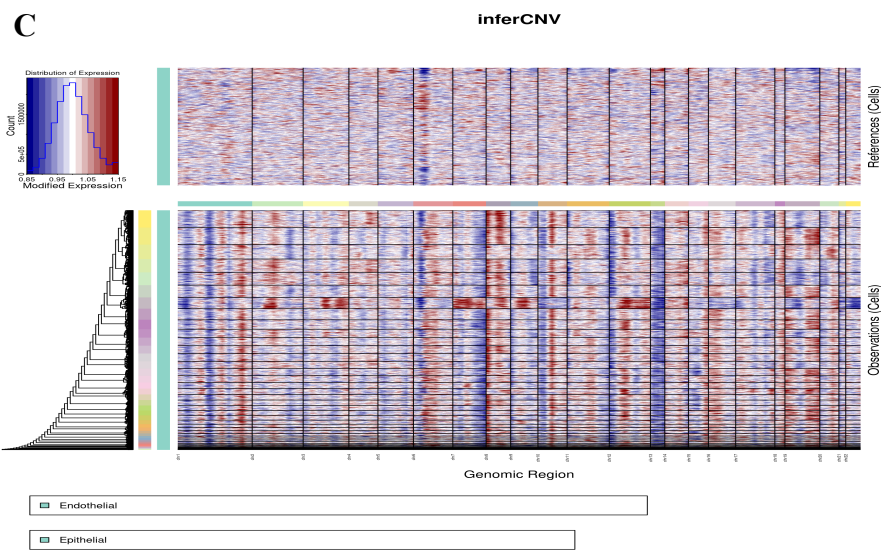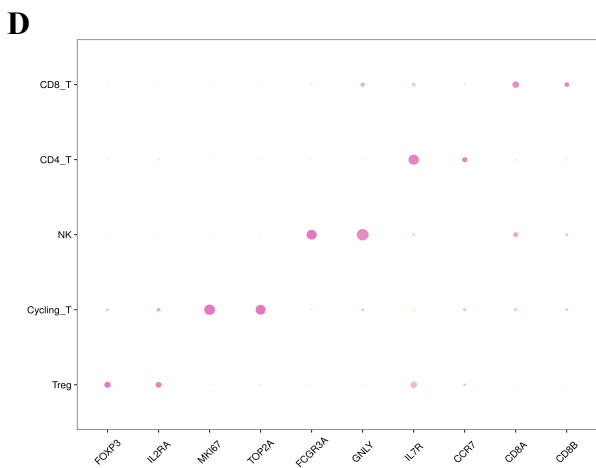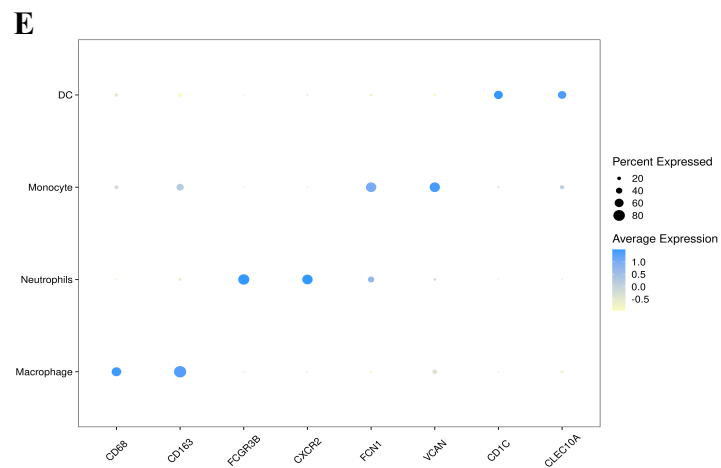

Supplement: Supplementary file 9 [file DataSheet1.pdf]

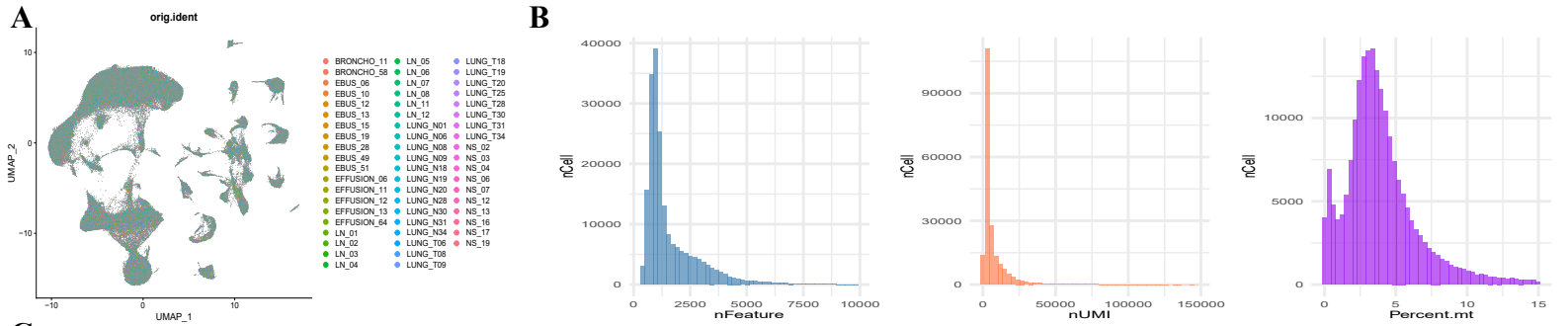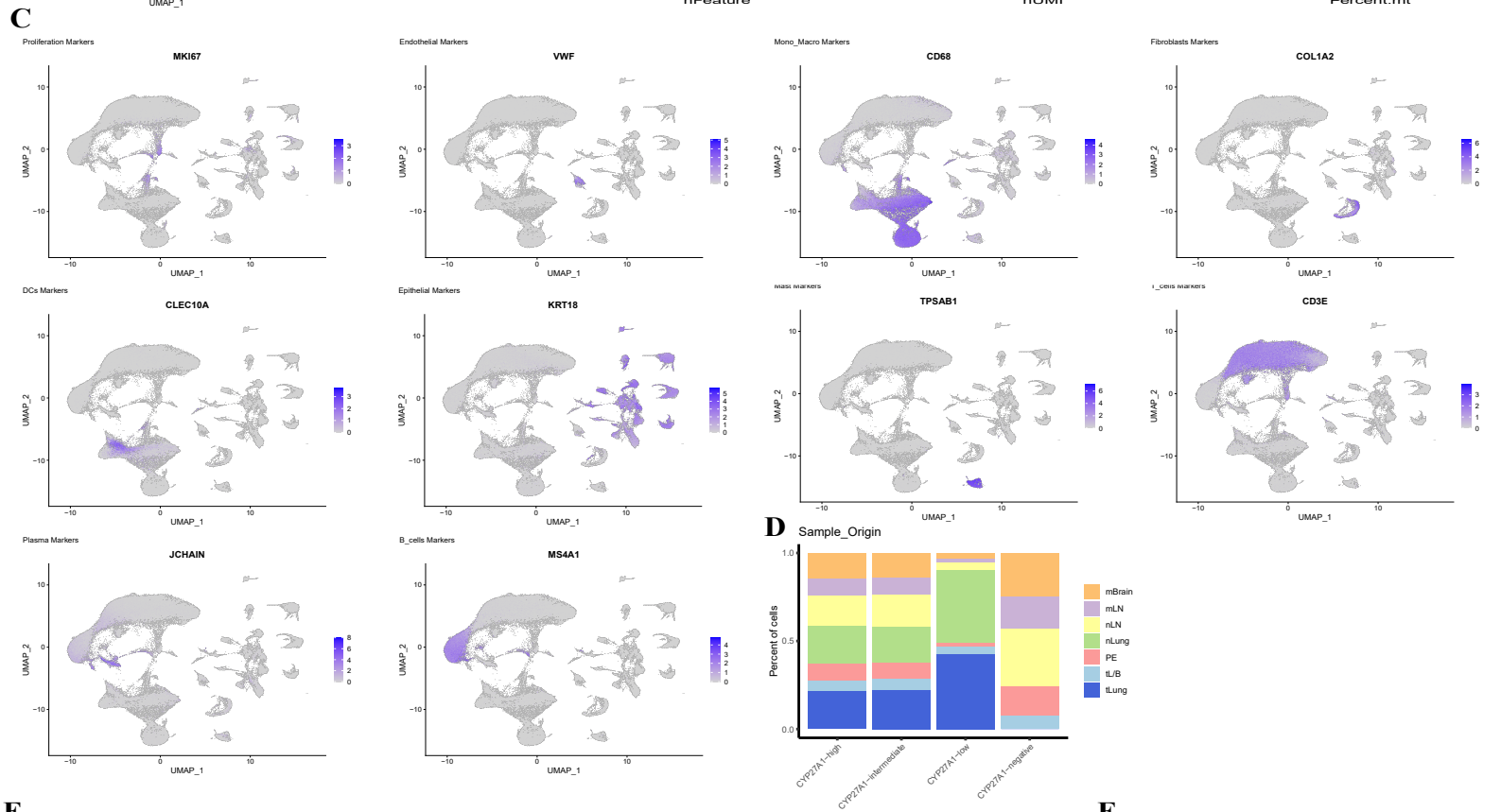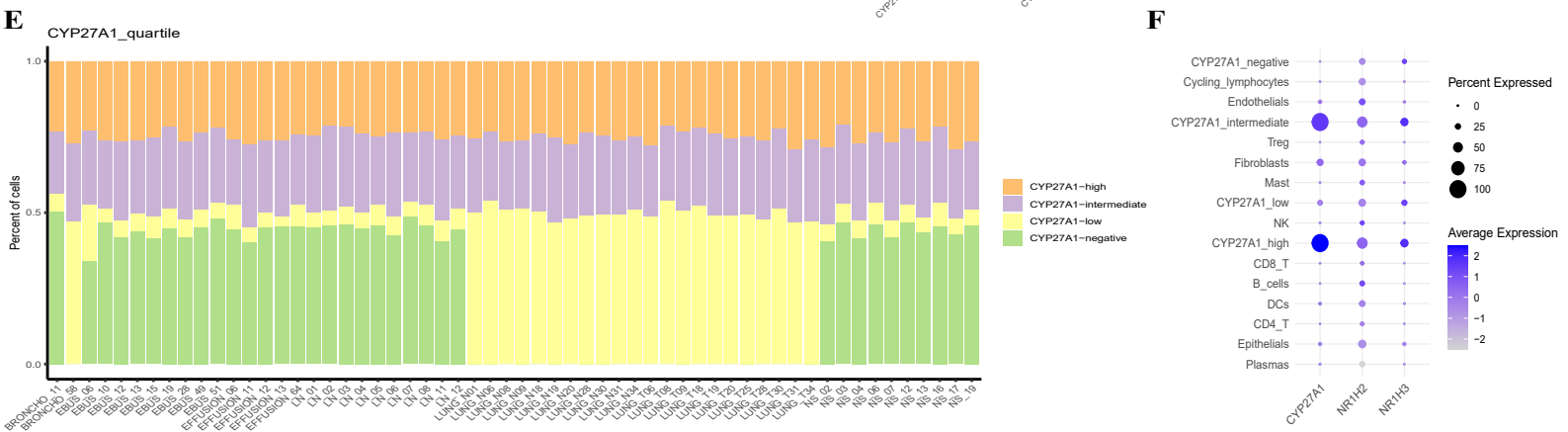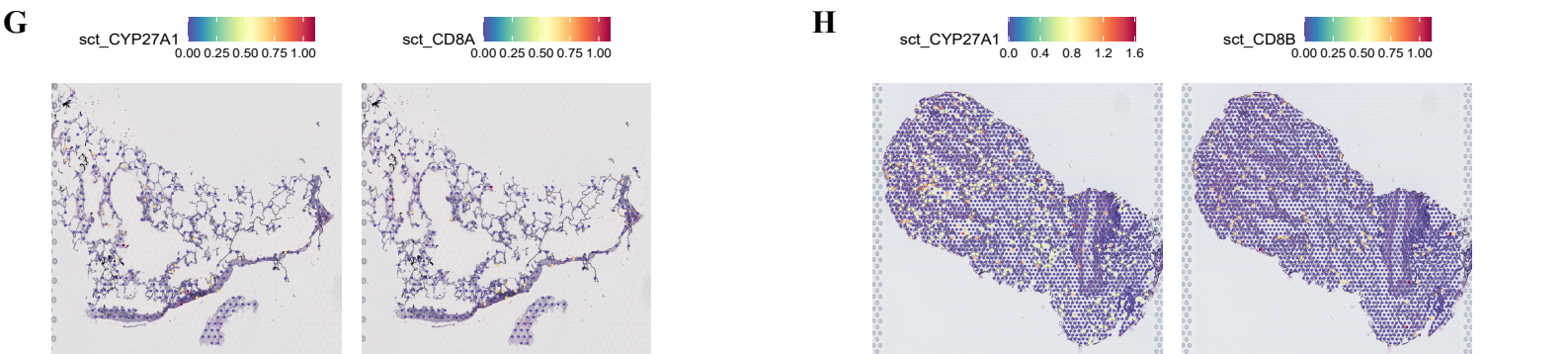

Supplement: Supplementary file 10 [file DataSheet2.pdf]

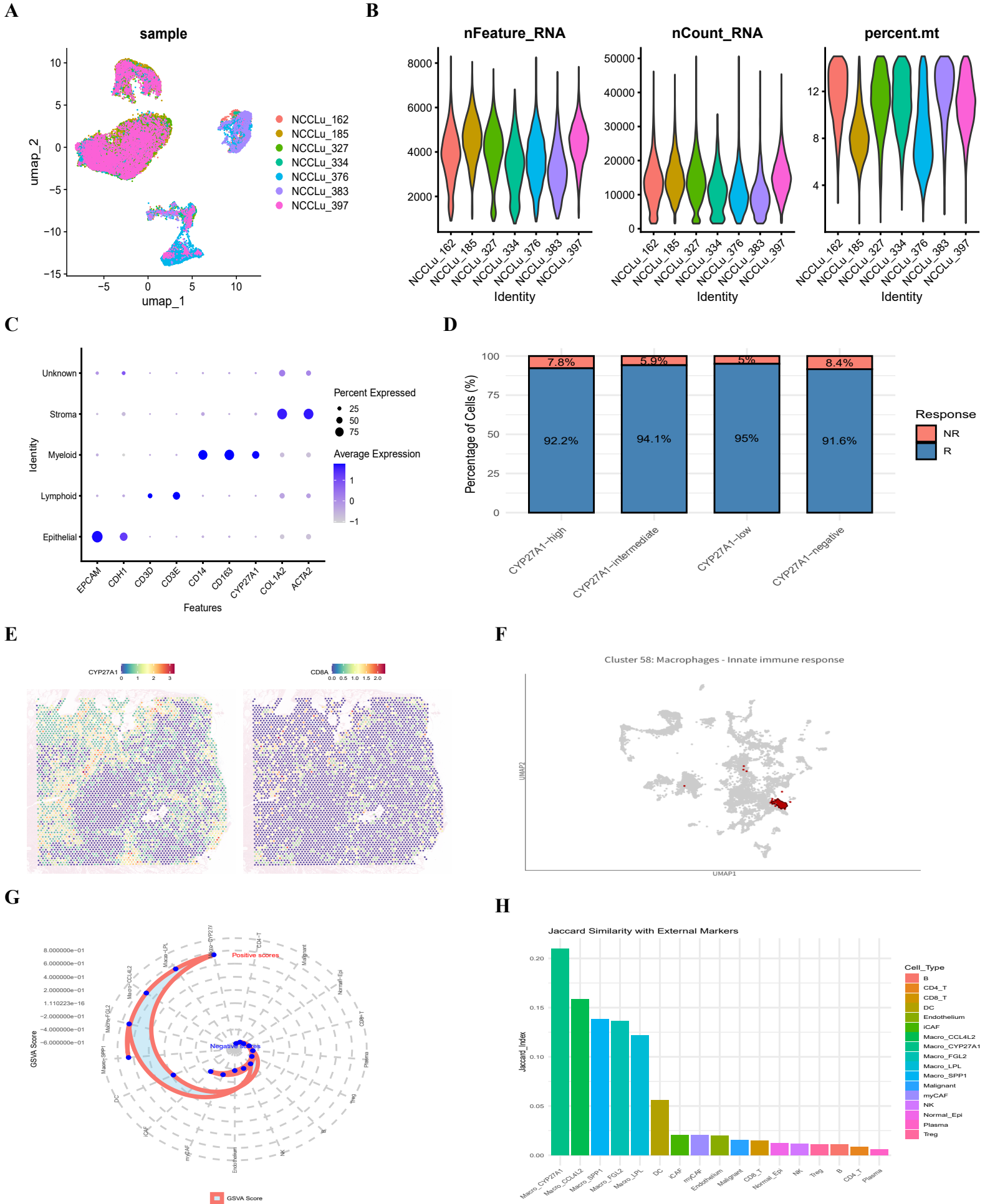

Supplement: Supplementary file 11 [file DataSheet3.pdf]
