# Supplementary material for: Integrative multi-omics and machine learning reveals the spatial niche distribution and role of CYP27A1+TAMs in immunotherapy response in non-small cell lung cancer
Source: Front Immunol. 2026 Feb 26;17:1782545. doi: 10.3389/fimmu.2026.1782545 (PMC12979556; doi:10.3389/fimmu.2026.1782545)
Supplement: Supplementary file 12 [file Table1.docx]

**Supplementary material.1 The coefficients (COEF) of the genes in the CMRS**

| **1** | BTK | -0.00555820682788055 |
| --- | --- | --- |
| **2** | CAT | -0.00596225443842648 |
| **3** | CYP27A1 | -0.0209840560523291 |
| **4** | DOCK4 | -0.0567961756846647 |
| **5** | DPYSL2 | -0.074055958164867 |
| **6** | FBP1 | -0.107018408072489 |
| **7** | METTL7A | -0.132913248919636 |

**Supplementary material.2 The primer sequences used in the paper**

GAPDH:Sense:TGTGTCCGTCGTGGATCTGA,Antisense:TTGCTGTTGAAGTCGCAGGAG.

CYP27A1:Sense:TACACCAATGTGAATCTGGC,Antisense:TAACCTCGTTTAAGGCATC.

**Supplementary material.3 Cluster 58, Macrophages - Innate immune response, specifically highly expresses 420 genes**

**Gene**

**ABCA1**

**ABCG1**

**ACER3**

**ACOT4**

**ACP2**

**ACP5**

**ADAM17**

**ADAMDEC1**

**ADAMTS2**

**ADAMTSL4**

**ADAP2**

**ADAR**

**ADCK2**

**ADGRE3**

**ADORA3**

**ADPGK**

**AGRP**

**AKAP10**

**ALDH2**

**ALOX5**

**ALOX5AP**

**ANKFY1**

**ANXA5**

**AP1B1**

**AP2A2**

**AP2S1**

**AP5B1**

**AP5Z1**

**APOE**

**ARAP1**

**ARHGAP4**

**ARPC3**

**ARRB1**

**ARRDC4**

**ATP10A**

**ATP5MGL**

**ATP6AP1**

**ATP6AP2**

**ATP6V0D1**

**ATP6V0D2**

**ATP6V1B2**

**ATP6V1F**

**ATP8B4**

**AWAT2**

**BAG4**

**BCAP31**

**BCAT1**

**BHLHE41**

**BLTP3A**

**BLVRA**

**BRI3**

**C1orf112**

**C1QA**

**C1QB**

**C1QC**

**C3AR1**

**C6orf62**

**CAPG**

**CCDC22**

**CCL13**

**CCL18**

**CCL24**

**CCL3**

**CCL3L1**

**CCL4L2**

**CCL7**

**CCR1**

**CD101**

**CD14**

**CD163**

**CD164**

**CD200R1**

**CD209**

**CD300LF**

**CD5L**

**CD68**

**CD84**

**CEACAM21**

**CEBPB**

**CHIT1**

**CIAO1**

**CLCN7**

**CLEC19A**

**CLEC5A**

**CLEC6A**

**CLTC**

**CMKLR1**

**COA6**

**COLEC12**

**COLGALT1**

**COMMD9**

**CPM**

**CREBL2**

**CREG1**

**CRYBB1**

**CSF1R**

**CTNS**

**CTSB**

**CTSC**

**CTSD**

**CTSL**

**CTSZ**

**CXCL5**

**CYBA**

**CYFIP1**

**CYP27A1**

**DAGLB**

**DCSTAMP**

**DDO**

**DDX60L**

**DENND4C**

**DNAJC13**

**DNAJC14**

**DNASE2**

**DOK1**

**DPEP2**

**DPH3**

**DSPP**

**DTX3L**

**DTX4**

**EGR2**

**EIF2AK2**

**ENSG00000250644**

**ENSG00000281613**

**F13A1**

**FAM50A**

**FAM89A**

**FAM91A1**

**FBP1**

**FBXO30**

**FCGR1A**

**FCGR2A**

**FCGR2C**

**FCGRT**

**FCHO2**

**FEZ2**

**FGF20**

**FHOD1**

**FIG4**

**FKBP15**

**FLVCR2**

**FMNL3**

**FOLR2**

**FOXRED2**

**FPGT**

**FPR3**

**FTL**

**FUCA1**

**FZD1**

**GAA**

**GAL3ST4**

**GAS2L3**

**GBGT1**

**GBP1**

**GCHFR**

**GLB1**

**GLIPR2**

**GLMP**

**GM2A**

**GNB2**

**GNB4**

**GNPDA1**

**GPATCH11**

**GPNMB**

**GPR34**

**GPR82**

**GRN**

**GSDMA**

**GSTO1**

**GUSB**

**HCLS1**

**HEATR3**

**HEXA**

**HEXB**

**HFE**

**HMOX1**

**HNMT**

**HPS6**

**HS1BP3**

**HUS1**

**IBSP**

**IFIT3**

**IFIT5**

**IFNA14**

**IGSF6**

**IL18**

**INHBA**

**ITGAM**

**ITPK1**

**ITSN1**

**JAML**

**KCNE1**

**KCTD12**

**KCTD5**

**KPTN**

**KRT79**

**KRTAP19-4**

**LACC1**

**LACTB**

**LAIR1**

**LAMP1**

**LAMP2**

**LGALS9**

**LGMN**

**LHFPL2**

**LILRB4**

**LILRB5**

**LIMK1**

**LIPA**

**LONRF3**

**LTA4H**

**LY96**

**LYVE1**

**M6PR**

**MAFB**

**MAMDC2**

**MAN1A1**

**MAN2B2**

**MARCO**

**MCEMP1**

**MCOLN1**

**MDM2**

**ME2**

**MEF2A**

**MERTK**

**MFSD1**

**MICAL1**

**MIIP**

**MILR1**

**MINK1**

**MKNK1**

**MMP19**

**MMP9**

**MOSPD2**

**MPND**

**MR1**

**MRC1**

**MS4A4A**

**MS4A4E**

**MS4A6A**

**MS4A7**

**MSR1**

**MTPN**

**MYO9B**

**NAGA**

**NAGLU**

**NAGPA**

**NAIP**

**NCEH1**

**NCF2**

**NCKAP1L**

**NCSTN**

**NEK6**

**NISCH**

**NLRC4**

**NOP10**

**NPC2**

**NPL**

**NR1H3**

**NUS1**

**OAS1**

**OAS2**

**OGFR**

**OGFRL1**

**OLR1**

**OR4B1**

**OR5AN1**

**OR6N1**

**OSBPL11**

**OSCAR**

**OSTM1**

**OTOA**

**P2RY12**

**P2RY13**

**PABPC4**

**PARP12**

**PARP9**

**PCYT1A**

**PDCL**

**PHLDA3**

**PI4K2A**

**PIK3R5**

**PKD2L1**

**PLA2G15**

**PLA2G7**

**PLBD1**

**PLD3**

**PLEKHB2**

**PLEKHM2**

**PLEKHO2**

**PLIN2**

**PLTP**

**PLXDC2**

**PNPLA6**

**PPARG**

**PPP1R9B**

**PPP3R1**

**PTAFR**

**RAB13**

**RAB20**

**RAB39A**

**RAB42**

**RETN**

**RGL1**

**RMDN3**

**RNASE1**

**RNASEL**

**RNF13**

**RNF135**

**RNPEP**

**RRAGC**

**SAMD9L**

**SCAMP2**

**SCARB2**

**SCN9A**

**SDCCAG8**

**SEC11A**

**SETDB2**

**SFMBT2**

**SGK3**

**SGPP1**

**SGSH**

**SH2D4B**

**SH3BGRL**

**SIDT2**

**SIGLEC1**

**SIGLEC10**

**SIGLEC15**

**SIRPA**

**SLC11A1**

**SLC15A3**

**SLC18B1**

**SLC25A24**

**SLC26A11**

**SLC31A1**

**SLC35F6**

**SLC37A2**

**SLC43A3**

**SLC46A1**

**SLC49A3**

**SLC8B1**

**SLCO2B1**

**SMCR8**

**SNTB1**

**SNX10**

**SNX6**

**SOAT1**

**SOCS6**

**SPI1**

**SPOCD1**

**SPP1**

**STAC**

**STX4**

**STX6**

**SUMF1**

**SUSD1**

**SYS1**

**TALDO1**

**TAS2R60**

**TBC1D16**

**TBC1D2**

**TBC1D9B**

**TBXAS1**

**TCF7L2**

**TCHH**

**TDRD3**

**TFEC**

**TFPT**

**TFRC**

**TGOLN2**

**TK2**

**TLR5**

**TLR7**

**TM4SF19**

**TM6SF1**

**TMC6**

**TMED5**

**TMEM106A**

**TMEM127**

**TMEM251**

**TMEM273**

**TMEM30A**

**TMEM63A**

**TMEM86A**

**TNFAIP8L2**

**TNFSF12**

**TNFSF12-TNFSF13**

**TNFSF18**

**TNS3**

**TPP1**

**TRA2B**

**TREM1**

**TREM2**

**TRIM14**

**TRIM21**

**TRIM25**

**TRIM65**

**TRPV2**

**TSPO**

**TTC39B**

**TTL**

**TTYH3**

**UAP1L1**

**UNC93B1**

**UPP1**

**UPRT**

**USF2**

**USP4**

**VASP**

**VAT1**

**VPS11**

**VPS18**

**VPS37C**

**VSIG4**

**WDR81**

**ZBTB8OS**

**ZDHHC24**

**ZNF316**

**ZNF687**

**ZNFX1**
